# Chemical Stabilization and Transformation of Schiff Base Scaffold: A Path toward Superior Platinum-Based Anti-Cancer Stem Cell Drugs

**DOI:** 10.1021/acs.jmedchem.6c01871

**Published:** 2026-08-14

**Authors:** Cynthia Sinaí Novoa-Ramirez, Jorge Melones Herrero, Susana Priego Gutierrez, Leticia Cubo Martín, Adoración Gómez Quiroga, Isabel Sanchez Pérez

**Affiliations:** † Universidad Autónoma de Madrid (UAM), Department of Inorganic Chemistry, Madrid 28049, Spain; ‡ Department of Biochemistry, School of Medicine, 16722 UAM, Madrid 28029, Spain; § Instituto de Investigaciones Biomédicas “Sols-Morreale” IIBM-CSIC-UAM, Madrid 28029, Spain; ∥ BIOPAC-IRYCIS, Madrid 28034, Spain; ⊥ Institute of Advanced Chemical Sciences (IAdChem), UAM, Madrid 28039, Spain; # Centro de Investigación Biomédica En Red de Enfermedades Raras (CIBERER) ISCIII, Madrid 28029, Spain

## Abstract

The clinical application of platinum-based anticancer agents is limited by poor aqueous stability, low solubility, and dose-limiting toxicities. Here, we report two Pt­(II) complexes based on a Schiff base ligand and its reduced imine derivative, designed to overcome these limitations. We show that ligand stabilization through imine reduction markedly improves solution stability, which emerges as an important contributor to interaction, cellular responses, and antiproliferative activity. ICP-MS analysis demonstrated efficient intracellular platinum accumulation with preferential nuclear localization. The lead compound also displayed potent activity against gastric cancer stem-like cells by impairing self-renewal and pluripotency. Transcriptomic and functional analyses associated this effect with suppression of stemness and epithelial-to-mesenchymal transition-related transcriptional programs, reduced OCT3/4 expression, and activation of DNA damage, apoptosis, and oxidative stress pathways. These findings identify a promising Pt­(II) scaffold for targeting cancer stem cell-associated phenotypes and establish ligand stabilization as an effective strategy for developing next-generation platinum anticancer agents.

## Graphical abstract

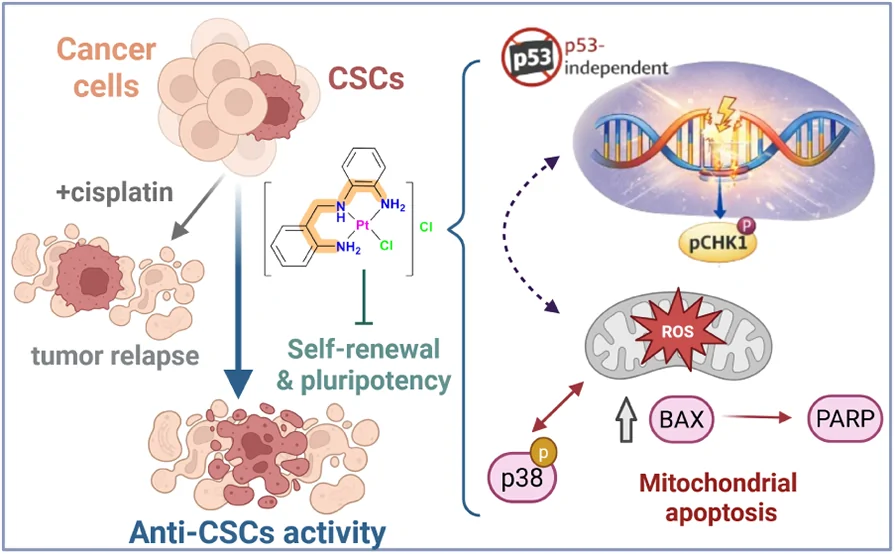

## Introduction

Gastric cancer (GC) remains one of the leading causes of cancer-related mortality worldwide, and its incidence is projected to continue to rise in the coming years.^1–3^ The clinical management of GC frequently relies on tumor resection, followed by FOLFOX-based chemotherapy (5-fluorouracil, leucovorin, and oxaliplatin). However, therapeutic efficacy is often limited by systemic toxicity, drug resistance, and disease recurrence.^4^ These limitations underscore the need for novel strategies to improve drug delivery and therapeutic outcomes in GCs. Tumor relapse and chemoresistance have been strongly associated with both intra- and intertumoral heterogeneity,^5,6^ a phenomenon driven in part by cancer stem cells (CSCs), defined by their ability to self-renew and differentiate into heterogeneous tumor cell populations.^7–9^ The maintenance of CSC properties is supported by aberrant activation of signaling pathways such as Notch, JAK/STAT, and epithelial-to-mesenchymal transition (EMT), among others,^6^ together with the regulation of pluripotency-related genes, including OCT4, SOX2, and NANOG. These processes collectively contribute to self-renewal, phenotypic plasticity, and therapeutic resistance, making these pathways relevant functional readouts for evaluating anticancer strategies.^6,10^

Platinum-based drugs are used in clinical practice. Cisplatin remains one of the most established and extensively studied agents, continuing to be a cornerstone in the treatment of several solid malignancies. Currently, it is employed in combination regimens for cervical cancer, including platinum–taxane or gemcitabine-based therapies, as well as in combination with targeted agents such as PARP inhibitors or bevacizumab.^11^ Despite its broad clinical utility, the therapeutic application of cisplatin is severely limited by dose-limiting toxicities, including nephrotoxicity, hepatotoxicity, hematotoxicity, and ototoxicity, as well as by the emergence of intrinsic and acquired resistance mechanisms.^12^ In addition, its limited aqueous solubility and unfavorable pharmacokinetic profile pose challenges for formulation and bioavailability.^13,14^ These drawbacks have driven sustained efforts toward the development of new platinum-based compounds with improved stability, reduced toxicity, and enhanced antitumor selectivity.^15,16^

Rationally designing the ligand environment is a key strategy for tuning the biological activity of platinum complexes, as ligands critically influence the stability, solubility, reactivity, and activation under physiological conditions. The incorporation of specific structural features, for example, aromatic moieties, may further enhance interactions with biomolecular targets,^17^ thereby contributing to improved antitumor activity and selectivity against CSC-associated phenotypes.^18^ Careful selection of both the metal center and the coordinating ligands therefore enables the development of metallodrugs with tailored chemical and biological properties. This concept is well illustrated by the historical evolution of the clinically used platinum compounds. Oxaliplatin, a third-generation platinum compound, was developed to retain antitumor activity while reducing certain toxicities associated with earlier drugs, such as cisplatin. Its distinct ligand structure contributes to different DNA adduct formations, improved stability, and a more favorable toxicity profile, making it particularly suitable for combination with other drugs. One such example is the perioperative FLOT regimen (5-fluorouracil, leucovorin, oxaliplatin, and docetaxel),^19–21^ which has become a standard treatment option in Europe for locally advanced GC, associated with superior overall survival outcomes.^22,23^

Within this context, Schiff base ligands have attracted considerable attention in coordination chemistry and medicinal inorganic chemistry. The electrophilic character of the imine carbon and the nucleophilic nature of the imine nitrogen confer a high degree of reactivity, enabling Schiff bases to interact with a wide range of biological species.^24,25^ Salen-type ligands, in particular, are tetradentate N_2_O_2_ donors that form stable chelates with transition metals and have been extensively explored in catalysis, with growing interest in their potential anticancer applications.^26,27^ Their ability to coordinate a wide variety of transition metals has led to the development of numerous Schiff base metal complexes with potent antiproliferative activity against different tumor types.^28^ These complexes act through multiple mechanisms, including DNA binding and damage, reactive oxygen species (ROS) generation, mitochondrial dysfunction, endoplasmic reticulum stress,^29^ inhibition of thioredoxin reductase,^30^ cell cycle arrest, apoptosis, and modulation of cancer stem cell-related pathways, highlighting Schiff bases as privileged scaffolds for the development of new metallodrugs. However, many metal complexes containing Schiff base ligands suffer from poor water solubility and limited stability in aqueous media, which represent major obstacles to biological studies. To address these limitations, ligands incorporating ionic functional groups, such as quaternary ammonium substituents^31,32^ or sulfonated aromatic rings,^33^ have been developed to enhance water solubility.

Another critical drawback of Schiff-base ligands is their susceptibility to hydrolysis under aqueous conditions. Studies on metal complexes bearing sulfonated salen ligands have shown that multiple species can form depending on the pH and solvent composition.^33^ Since biological assays are typically conducted in aqueous environments, hydrolytic instability poses a significant challenge. Consequently, strategies aimed at stabilizing the Schiff-base framework while preserving its favorable pharmacological properties have become an important focus in the design of next-generation metal-based anticancer agents.^28,29^

One effective strategy for overcoming their hydrolytic lability is the chemical reduction of the imine functionality to yield the corresponding amine derivatives. Reduced Schiff bases (salan-type ligands) generally exhibit enhanced hydrolytic stability and improved aqueous solubility, which makes them more suitable for biological applications. Despite these challenges, Schiff base ligands offer significant advantages in the design of platinum-based anticancer agents.^34^ Their electronic properties can be readily tuned through structural modification, allowing precise control over the electronic environment of the platinum center and, consequently, its reactivity toward biological targets. Moreover, aromatic Schiff base frameworks may promote stronger interactions with DNA, potentially enhancing the antiproliferative activity.^35,36^

Despite the growing interest in Pt­(II)-Schiff base complexes as anticancer agents, most studies remain focused on cytotoxicity, apoptosis, DNA interaction, ROS production, or the regulation of selected genes and proteins. Comprehensive systems-level approaches, particularly transcriptome-wide analyses based on RNA sequencing, remain largely unexplored in this family of compounds. This lack of integrative “omics” studies limits the understanding of the molecular programs underlying their antitumor and anti-CSC activity.

In this work, we report the synthesis, structural characterization, and solution behavior of two novel Pt­(II) complexes based on a Schiff base ligand (**L** ^**1**^) and its reduced amine derivative (**L** ^**1**^ **H**). Their stability under biologically relevant conditions was investigated, and their interaction with DNA was evaluated using electrophoresis and viscosity assays. In addition, the interaction of these complexes with the fluorescent protein bovine serum albumin (BSA) was analyzed by direct fluorescence titration assays. Furthermore, we examined the antiproliferative activity in GC cell lines, analyzed their effects on cell cycle profile, the activation of DNA damage response, apoptosis induction, mitochondrial dysfunction, and their impact on cancer stem-like cell viability and self-renewal. Although bearing Schiff base ligands, such as Cu­(II)-Schiff base complexes,^37,38^ has previously demonstrated activity against CSCs, to the best of our knowledge, no studies have reported Pt­(II)-Schiff base complexes with anti-CSC activity. Finally, RNA sequencing was performed and allowed the identification of key pathways involved in cell death (ROS, apoptosis, DNA damage) and, specifically, a significant downregulation in the epithelial-to-mesenchymal transition. Functional studies reinforced this transcriptomic profiling, highlighting the potential of the cationic complex to impair cell migration and invasion, together with the CSC signaling network, opening a new avenue in platinum-based anticancer research.

## Results and Discussion

### Synthesis, Transformation, and Characterization of Platinum Complexes

First, we explored the potential of tridentate scaffolds to enhance the stability of platinum complexes while maintaining a reactive chloride site and balancing structural integrity with potential biological activity. To achieve this goal, we designed N,N,N-donor systems as ligands with different linker lengths (Figure 1). Multiple coordination attempts were carried out to obtain a family of Pt­(II) complexes with **L** ^**1**^ **H** to **L** ^**4**^ **H** (–17).

**Figure 1:**
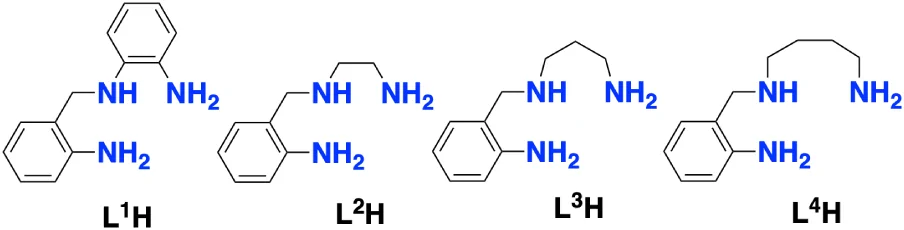
Structure of the synthesized ligands.

Different platinum precursors, including K_2_[PtCl_4_] and *cis*-[PtCl_2_(DMSO)_2_], were explored under a range of reaction conditions: from room temperature to 70 °C, varying reaction times and solvents (methanol, ethanol, or chloroform), and even running in anaerobic conditions. The reactions with **L** ^**1**^ **H** allowed the isolation of well-defined platinum complexes, whereas the ligands **L** ^**2**^ **H** to **L** ^**4**^ **H** always afforded complicated mixtures with too many species or decomposition (typically forming black solids). These mixtures were also treated with ionic sources such as KCl or NH_4_PF_6_ to promote precipitation of the possible complexes, where no solid products were isolated. These experiments indicate that, although several ligands were achieved, any reaction condition explored to access a broader series of complexes was unsuccessful. The coordination chemistry of the tridentate systems bearing aliphatic linkers with Pt­(II) proved to be highly sensitive to the reaction conditions, leading to competing species impossible to isolate. Consequently, this approach was discarded, and we focused on the phenyl-based system **L** ^**1**^ **H**, whose synthetic routes are shown in Scheme 1. The **L** ^**1**^ synthesis was performed in ethanol by the condensation reaction of 2-aminobenzaldehyde with 1,2-phenylenediamine. To achieve controlled monocondensation, the addition of 2-aminobenzaldehyde must be dropwise. **L** ^**1**^ **H** was achieved by reduction of **L** ^**1**^ using up to 20 equiv of NaBH_4_ to reach a good yield. Detailed characterization data for **L** ^**1**^ and **L** ^**1**^ **H** in DMSO-*d*_6_ and MeOH-*d*_4_, respectively, are provided in the Experimental Section and included in the Supporting Information (Figure S1–S11). For **L** ^**1**^, the ^1^H and ^13^C NMR spectra displayed the expected imine proton and carbon at characteristic downfield shifts (*δ*_H(7)_ = 8.60 ppm and *δ*_C(7)_= 161.21 ppm), along with aromatic signals consistent with the proposed structure; the IR spectrum also showed the diagnostic C=N stretching band at 1610 cm^–1^. After the reduction of **L** ^**1**^, **L** ^**1**^ **H** showed clear spectral changes: the imine signal disappeared, and a new benzylic −CH_2_–NH– appeared significantly upfield (*δ*_H(7)_ = 4.22 ppm and *δ*_C(7)_= 57.77 ppm), confirming the reaction. The loss of the imine IR band, together with mass spectrometry and elemental analysis consistent with the expected formula, verified that **L** ^**1**^ **H** was successfully obtained.

**Scheme 1:**
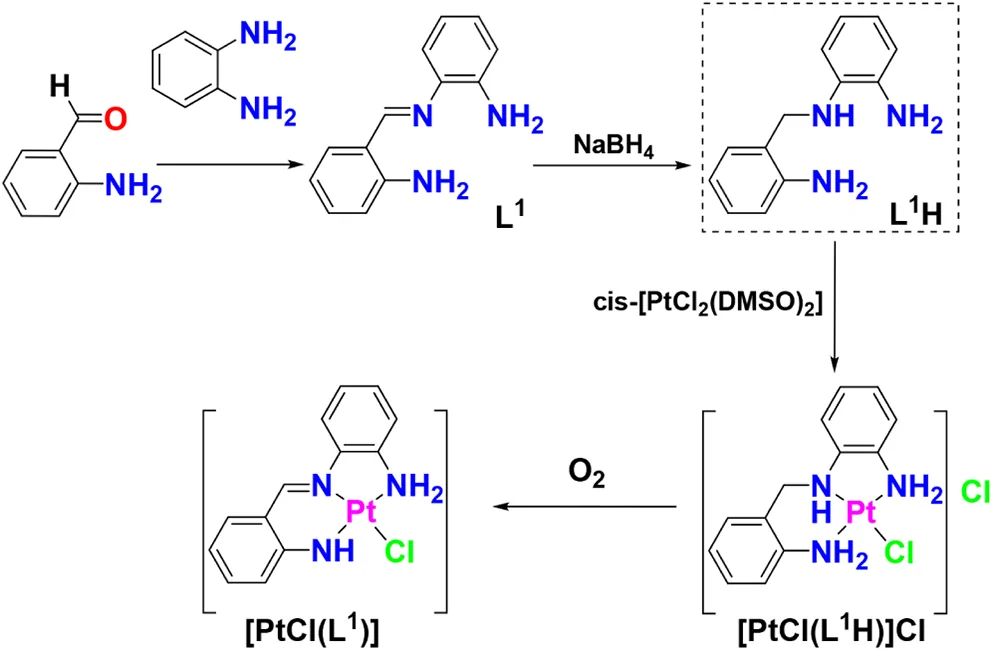
Synthesis of Platinum Complexes with the Phenyl-Based System **L** ^**1**^ **H**

*cis*-[PtCl_2_(DMSO)_2_] reacts with a small excess of **L** ^**1**^ **H** in CHCl_3_ at 60 °C to afford the complex **[PtCl­(L** ^**1**^ **H)]­Cl.** Elemental analysis and mass spectrometry data agreed (Figure S18: 443 *m*/*z*) with the proposed formula [PtC_13_H_15_N_3_Cl]^+^. The ^1^H NMR spectra of **[PtCl­(L** ^**1**^ **H)]­Cl** were recorded in D_2_O due to the water solubility of the complex, facilitating a discussion on its stability in solution (see Stability in Aqueous Solution section ). The spectra showed eight aromatic protons and an additional signal for the two aliphatic protons, which proves the complex’s stability (Figure S19). Besides, the ^13^C NMR spectra contained the 12 aromatic and one aliphatic carbon signals expected (Figure S20) and the assignment is supported by the 2D NMR spectra (Figures S21–S24). The coordination of the ligand to platinum is corroborated with ^195^Pt NMR which showed a signal at −2618 ppm (Figure S25).^39^

In addition, the characterization was performed by NMR in MeOH-*d*_4_ which allows comparison with the free ligand. The detected signals in ^1^H NMR (Figure 2) exhibited a downfield shift relative to the free ligand, confirming its coordination to the platinum center. A significant change was observed for the CH_2_–signal, which was a sharp singlet at 4.22 ppm in the spectra of the free ligand and shifted to 4.95 ppm in the platinum complex spectra. In the latter, the signal was as a doublet of doublets consistent with the presence of a diastereotopic protons resulting from platinum coordination and the square-planar geometry, which restricts free rotation of the −CH_2_ group.

**Figure 2:**
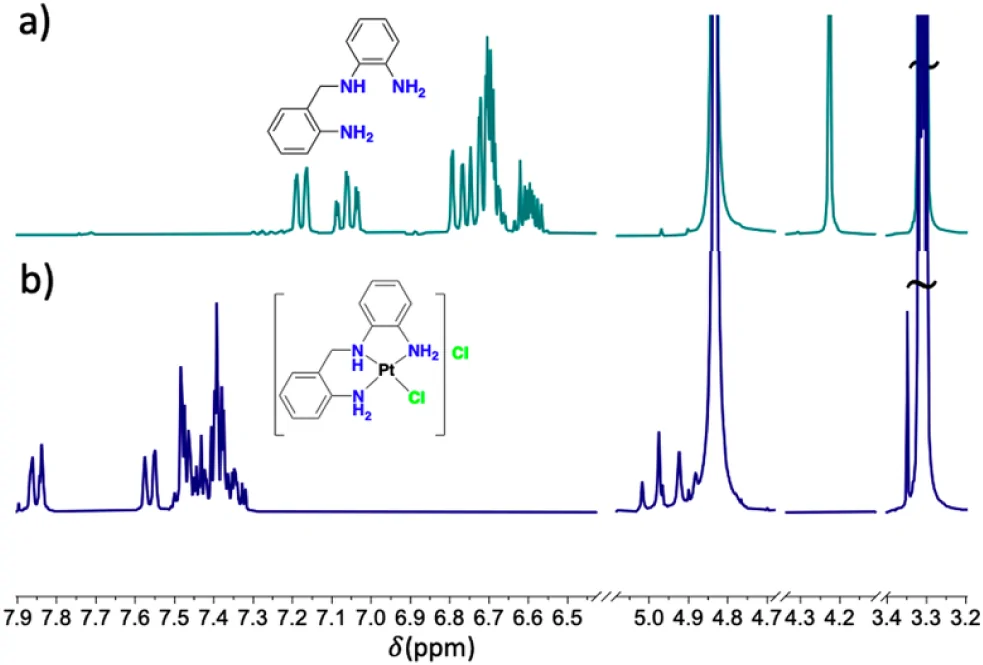
^1^H NMR spectra of: **a)** ligand **L^1^H** and **b)** complex **[PtCl­(L^1^H)]­Cl** in MeOH-*d*_4_.

[**PtCl­(L** ^**1**^ **H)]­Cl** exhibited solution stability in MeOH-*d*_4_ for up to 48 h, as evidenced by the ^1^H NMR spectra (Figure S26). However, under aerobic conditions, a secondary product was detected after 3 days. The new set of signals was assigned to the complex **[PtCl­(L** ^**1**^)] coordinated with the oxidized and deprotonated ligand (**L** ^**1**^). This reactivity has been previously reported for planar complexes containing salen-type ligands upon exposure to air or peroxides.^40^ The observed transformation corresponds to an oxidative dehydrogenation, converting the aliphatic (−CH_2_–NH−) fragment into a conjugated imine group (−CH=N−). Single crystals of **[PtCl­(L** ^**1**^)] from a methanol solution of **[PtCl­(L** ^**1**^ **H)]­Cl** through slow evaporation were analyzed by X-ray diffraction and confirmed the transformation. As far as we know, we are reporting for the first time an oxidative dehydrogenation to produce the tridentate NNN Schiff base complex **[PtCl­(L** ^**1**^)]. This reaction proved crucial, as it enabled the isolation of **[PtCl­(L** ^**1**^)], whereas direct reactions of **L** ^**1**^ with various platinum precursors under different conditions yielded unworkable purification mixtures. An optimized method to isolate **[PtCl­(L** ^**1**^)] was thus established by a stream of air to obtain a scaled and good yield reaction, as described in the Experimental section. During this optimization of the synthesis procedure, we detected an intermediate species, **[PtCl­(L** ^**1**^ **)]­Cl** (Figure S27). The characterization of this intermediate was performed by ^1^H NMR and X-ray diffraction; however, the transformation of this complex takes place in solution to [**PtCl­(L** ^**1**^)]. Due to this reactivity, we discarded this compound for further biological studies.

[**PtCl­(L** ^**1**^)] general formula was corroborated by elemental and thermogravimetry analysis, TGA (Figure S28). The MALDI mass spectra (Figure S29) showed a main peak [M + H] at 442 *m*/*z* corresponding to the formula PtC_13_H_13_N_3_Cl. The characterization by^1^H NMR was performed in DMSO-*d*_6_ because of its low solubility in water (Figure S30), and most of the signal chemical shifts were downfielded compared to those of the free ligand (Figure 3), except for a triplet at δ 6.33, attributed to H(4). The ^13^C NMR spectrum showed the 12 expected aromatic and one imine carbon signals, corroborated with the 2D spectra (Figures S31–S35). Additionally, the ^195^Pt NMR signal at −2037 ppm proved the ligand coordination (Figure S36).^39^

**Figure 3:**
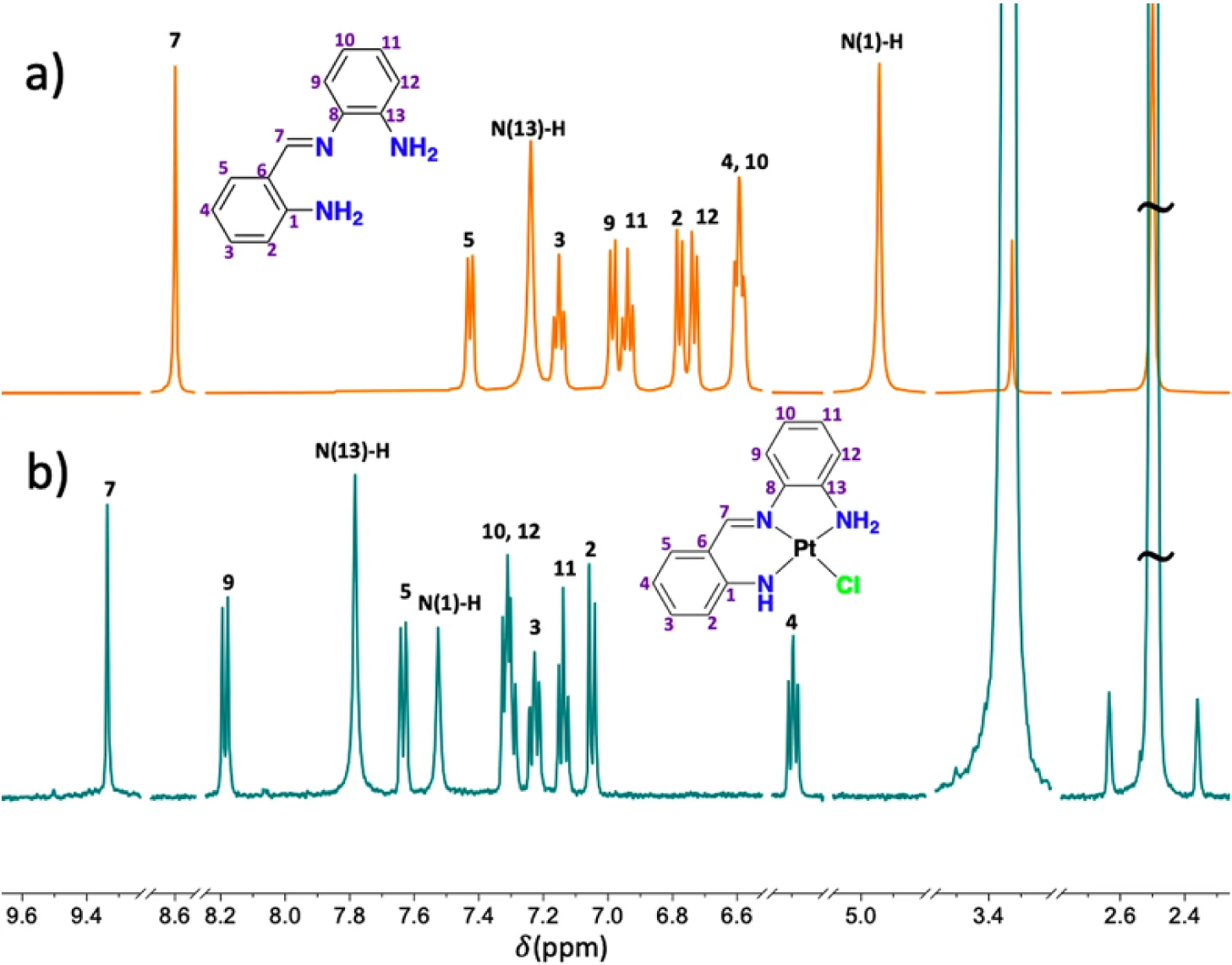
^1^H NMR spectra of: **a)** ligand **L^1^** and **b)** complex **[PtCl­(L^1^**)] in DMSO-*d*_6_.

### Single-Crystal X-Ray Diffraction Analysis

Single-crystal X-ray diffraction analysis confirmed the molecular structures of both the neutral **[PtCl­(L** ^**1**^ **)]·(H**_**2**_ **O)**_**0.25**_ (Figure 4a) and the ionic **[PtCl­(L** ^**1**^ **)]­Cl·H**_**2**_ **O** (Figure 4b) complexes, revealing similar primary coordination geometries but distinct packing arrangements driven by their lattice compositions. The water molecules are lattice solvent molecules and are not coordinated to the Pt­(II) center. In both structures, the Pt­(II) center adopts a square-planar geometry coordinated by the tridentate ligand **L** ^**1**^, which binds through two amine nitrogen atoms (N(1), N(3)) and one imine nitrogen (N(2)). A terminal chloride completes the coordination sphere. Selected bond lengths and angles are summarized in Table 1. The Pt–N and Pt–Cl bond distances are comparable between both structures: for **[PtCl­(L** ^**1**^ **)]·(H**_**2**_ **O)**_**0.25**_, Pt–N ranges from 1.964(7) to 2.054(7) Å and Pt–Cl = 2.331(2) Å; while for **[PtCl­(L** ^**1**^ **)]­Cl·H**_**2**_ **O**, Pt–N distances are between 1.992(8) and 2.029(5) Å, and Pt–Cl = 2.2961(9) Å. Comparison of the two structures reveals that the Pt–N(3) bond is shorter in the neutral species (1.964(7) Å) than in the cationic salt (2.028(3) Å). This difference in bond length could be related to the increase in the donor strength of the former ligand when it is present in the cationic salt. These values are consistent with square-planar Pt­(II) complexes and reflect a stable, undistorted coordination environment. The calculated τ_4_ parameters are low in both cases (0.051 and 0.088–0.14), indicating only minor deviations from ideal square-planarity.

**Figure 4:**
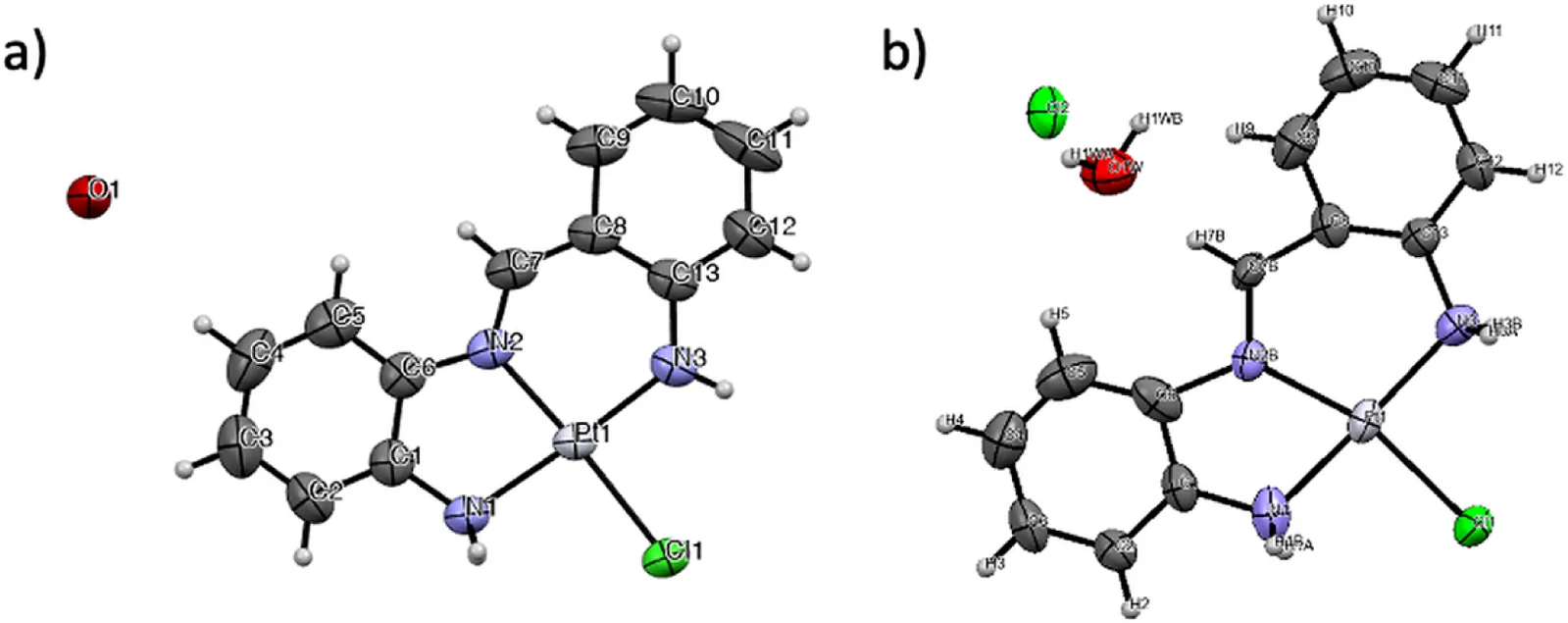
X-ray ORTEP figure of complex: **a) [PtCl­(L^1^)]·(H_2_O)_0.25_** and **b) [PtCl­(L^1^)]­Cl·H_2_O** within conformation B (CCDC ID: **2517080** and **2517078**). The lattice water molecule and chloride counterion are shown as ball-and-stick representations.

**Table 1:** Selected Bond Lengths [Å] and Angles [°] **[PtCl­(L^1^**)] and (B)-**[PtCl­(L^1^)]­Cl** ^a^

[PtCl(L^1^)]·(H_2_O)_0.25_				[PtCl(L^1^)]Cl·H_2_O			
Bond lengths (Å)							
C(1)–N(1)	1.442(11)	C(13)–N(3)	1.358(10)	C(1)–N(1)	1.450(5)	C(13)–N(3)	1.454(5)
C(1)–C(6)	1.398(12)			C(1)–C(6)	1.388(6)		
C(6)–N(2)	1.441(11)	Pt(1)–N(1)	2.054(7)	C(6)-N(2B)	1.529(9)	Pt(1)–N(1)	2.020(4)
C(7)–C(8)	1.398(13)	Pt(1)–N(2)	1.976(7)	C(7B)-C(8)	1.421(10)	Pt(1)-N(2B)	1.992(8)
N(7)–C(2)	1.304(11)	Pt(1)–N(3)	1.964(7)	C(7B)-N(2B)	1.358(12)	Pt(1)–N(3)	2.028(3)
C(8)–C(13)	1.426(13)	Pt(1)–Cl(1)	2.331(2)	C(8)–C(13)	1.387(5)	Pt(1)–Cl(1)	2.296(9)

Bond angles (°)			
N(1)–Pt(1)–N(2)	82.7(3)	N(1)–Pt(1)–N(2)	72.7(3)
N(1)–Pt(1)–N(3)	176.6(3)	N(1)–Pt(1)–N(3)	177.4(2)
N(1)–Pt(1)–Cl(1)	93.3(2)	N(1)–Pt(1)–Cl(1)	90.7(11)
N(2)–Pt(1)–N(3)	93.9(3)	N(2B)-Pt(1)–N(3)	105.9(3)
N(2)–Pt(1)–Cl(1)	176.1(2)	N(2B)-Pt(1)–Cl(1)	163.2(3)
N(3)–Pt(1)–Cl(1)	90.0(2)	N(3)–Pt(1)–Cl(1)	90.8(9)

a Crystal data and structure refinement for both complexes are collected in .

Crystallographic disorder is observed only in **[PtCl­(L** ^**1**^ **)]­Cl·H**_**2**_ **O**, where the chelate ring displays two alternative orientations at the imine site (C­(7A/B)/N­(2A/B)), refined with occupancies of 0.658(5) and 0.342(5). This localized disorder accounts for small variations in the associated C–N and C–C bond metrics and the modest angular spread at the metal center.

While both complexes share a nearly identical inner coordination sphere, their supramolecular interactions differ significantly. In the neutral complex **[PtCl­(L** ^**1**^)], molecules are linked via intermolecular N–H···Cl hydrogen bonds, forming one-dimensional chains that propagate along the crystallographic *b*-axis (Figure 5a). Additional stabilization is provided by C–H···π interactions, and a short Pt···Pt contact of 3.353 Å suggests the presence of weak metallophilic interactions, often observed in d^8^ closed-shell Pt­(II) systems.

**Figure 5:**
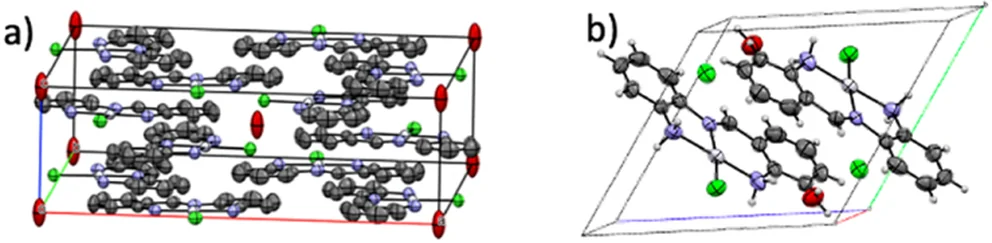
Crystal array packing of complexes: **a) [PtCl­(L^1^)]·(H_2_O)_0.25_** and **b) [PtCl­(L^1^)]­Cl·H_2_O**.

In contrast, the salt **[PtCl­(L** ^**1**^ **)]­Cl·H**_**2**_ **O** forms an extended three-dimensional hydrogen-bonding network, mediated by interactions between the coordinated cation, the lattice chloride ion, and a water molecule. Notably, N–H···Cl and O–H···Cl contacts dominate the packing, with the crystallization water acting both as a donor and an acceptor in several key interactions. A bifurcated N–H···(Cl, O) motif further stabilizes the assembly, as shown in Figure 5b.

The differences between both structures illustrate the impact of counterions and solvation on crystal packing. While both complexes preserve a robust and chemically equivalent Pt­(II) coordination core, the presence of the lattice chloride and water molecules in the salt modifies the hydrogen-bonding landscape, preventing close Pt···Pt approaches and enabling greater packing flexibility through solvent-mediated contacts.

### Stability of the Compounds in Aqueous Solution

Stability studies are essential for coordination-based drug candidates, given their susceptibility to ligand exchange or decomposition under different solution conditions. For example, cisplatin is prepared in an aqueous solution with a high concentration of chlorides to ensure its identity in formulations. In addition, solvents such as water and DMSO induce speciation. DMSO is a coordinating solvent, which may produce deactivation,^41^ activation,^42^ or a mediated effect.^43^ Therefore, stability studies in biological conditions with analytical techniques are required to identify the active species.

### Ligands L^1^ and L^1^H

The stability of ligands **L** ^**1**^ and **L** ^**1**^ **H** was monitored by UV–vis spectroscopy in a water/1% (v/v) DMSO mixture at 37 °C for 24 h. **L** ^**1**^ UV spectra revealed signals at 230, 257, and 290 nm (π–π* transitions), and one at 362 nm with a lower molar absorptivity (n−π* transition). For **L** ^**1**^ **H**, a maximum at 288 nm and a shoulder at 240 nm, corresponding to π–π* transitions, were observed.^44^ After 24 h, both ligands’ UV spectra profiles showed no significant changes (Figures S37 and S38) confirming their solution stability.

### Complexes [PtCl­(L^1^H)]Cl and [PtCl­(L^1^)]

The structural stability of the complex **[PtCl­(L** ^**1**^ **H)]­Cl** in water was evaluated by the viscosity of CT-DNA, expressed through ^1^H and^195^ Pt NMR spectroscopy over 24 h. The aromatic protons remained unchanged throughout the experiment, indicating that the complex did not degrade (Figure 6). The H(7) proton (−CH_2_−) appeared as a broad singlet at t:0 and gradually broadened until it was resolved as a doublet at 5 h in concentrated samples (2.78 mM), suggesting the conversion of H(7) into diastereotopic protons (Figure 6a). In diluted samples (0.46 mM), this singlet remained unchanged over time, confirming that the observed phenomenon is associated with concentration-dependent intermolecular interactions (Figure 6b). The^195^ Pt NMR spectra displayed a single signal at −2618 ppm at both t:0 and 24 h, confirming that the coordination environment of the metal center was preserved throughout the experiment (Figure S39).

**Figure 6:**
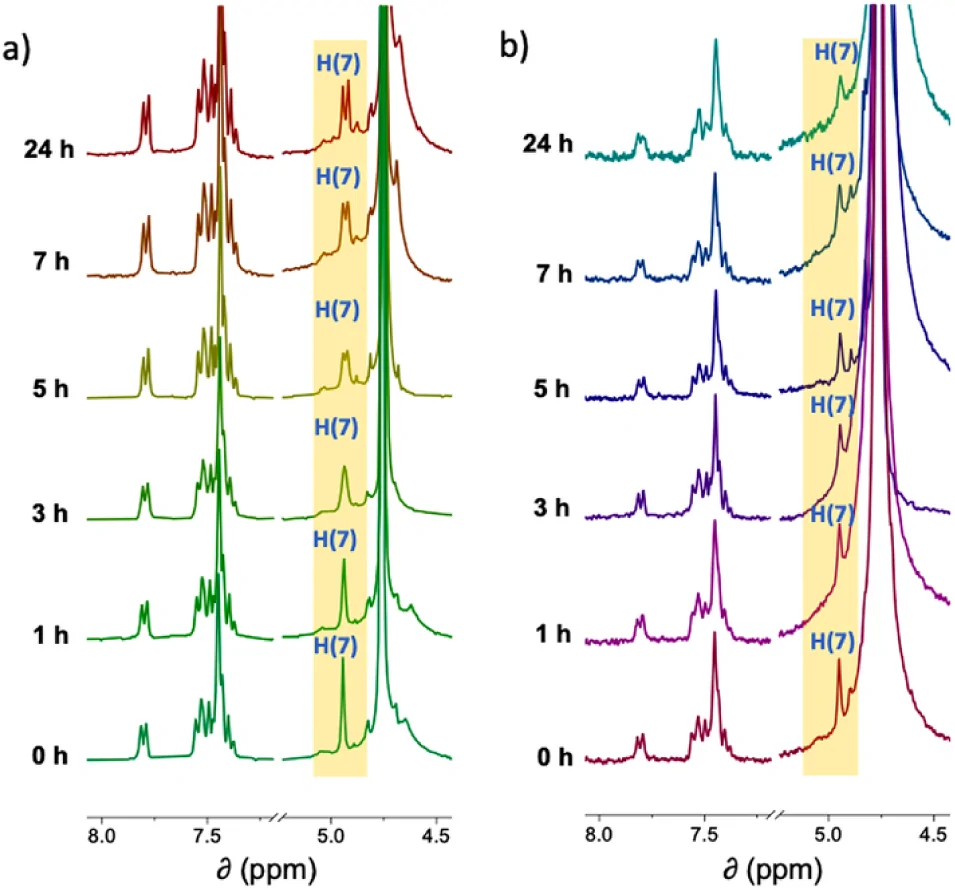
^1^H NMR stability test of **[PtCl­(L^1^H)]­Cl** at: **a)** 2.78 mM and **b)** 0.46 mM in D_2_O and TSP as reference.

ESI-MS analysis in water reveals exclusively a molecular ion at *m*/*z* 443, corresponding to **[PtCl­(L** ^**1**^ **H)]** ^**+**^, both at t_0_ and 4 h. No signals attributable to aquation, chloride substitution, or fragmentation were detected, which confirms the chemical stability of the complex during the first hours in solution (Figure S40).

The **[PtCl­(L** ^**1**^ **H)]­Cl** complex in aqueous and biological buffer solutions was monitored by UV–Vis spectroscopy over 24 h at concentrations ranging from 10 to 80 μM (Figures S41–S45). The spectra recorded in Tris-HCl and NaClO_4_ were analyzed together. In both cases, the same spectral profile was observed from t = 0 to 24 h. The UV–vis bands were at 260 nm, 322 nm, and a low-energy band at 503 nm, which might be assigned to spin-forbidden d–d transitions for square planar complexes, according to Gray and Mason^45^ A decrease in absorbance was detected in the high-concentration samples, accompanied by simultaneous precipitation. Analysis of the residue confirmed the chemical identity and high purity of the complex. The molar absorptivity coefficient of the spectra in water (Figure S41) does not increase linearly with the increasing concentration, indicating a deviation from the Beer–Lambert law. During the first 5 h, the absorptivity increases; however, after 24 h, the absorptivity slightly decreases. These phenomena are consistent with an aggregation/reorganization process, which was further analyzed by DLS.

Phosphate buffer has been reported as a protective medium for biological molecules and provides a model of physiologically relevant conditions.^46^ Additionally, platinum drugs are sometimes markedly affected by the presence of phosphate ions, which can coordinate with *trans* complexes or even with cisplatin.^47^ Thus, the behavior of **[PtCl­(L** ^**1**^ **H)]­Cl** in 20 mM phosphate buffer (pH 7.4) was analyzed by UV–vis spectroscopy, revealing spectral profiles similar to those observed using Tris-HCl and NaClO_4_ buffers. These results indicate that this complex appears to be stable under physiological-like conditions.

The hydrodynamic particle size and zeta potential data for the aqueous complex solution at 0, 5, and 24 h are summarized in Table 2. At t_0_, the complex formed aggregates of varying sizes (647 and 106 nm; ζ = −0.5 mV), which supports our hypothesis regarding the deviation from the Beer–Lambert law observed via UV–Vis. After 5 h, the large aggregates persisted (595 nm), and the zeta potential increased to +12.8 mV, indicating a possible aggregate reorganization. After 24 h, the histogram presented a broadened peak, from 251 to 712 nm, which was difficult to interpret. Since the zeta potential reached +27.2 mV, we think this was probably caused by a possible polydispersity of the sample.^48^

**Table 2:** Particle Size and ζ-Potential of 40 μM Solution of **[PtCl­(L^1^H)]­Cl** in Water at Different Times

Time	t_0_	5 h	24 h
Size (nm)	647 ± 56	595 ± 56	251–712
	106 ± 16	153 ± 16	
ζ-potential (mV)	-0.50 ± 0.37	12.8 ±1.6	27.2 ±1.9

In 0.9% (w/v) NaCl, the complex initially showed the same profile; the absorbance decreased by half after 20 min and continued slowing down with simultaneous precipitation. Overall, in Tris–HCl, NaCl, and NaClO_4_, the profile of the spectra remained constant, suggesting no chemical changes in the complex.

**[PtCl­(L** ^**1**^)] complex is not soluble in water, so an ^1^H NMR stability test was performed in DMSO-*d*_6_. The complex spectra remained stable over time (Figure 7) meaning no DMSO coordination occurred. Then, two drops of D_2_O were added, since DMSO is known to be more coordinated in the presence of water.^49^ After D_2_O addition, the N–H signals interchanged, and the aromatic protons H(7), H(9), and H(2) broadened, with H(2) changing from a doublet to a broad triplet. From this point up to 24 h, the spectra remained unchanged. After 24 h, 2D HSQC showed no additional cross-peaks in the DMSO solvent signal, confirming the absence of DMSO coordination to Pt­(II) (Figure S46).

**Figure 7:**
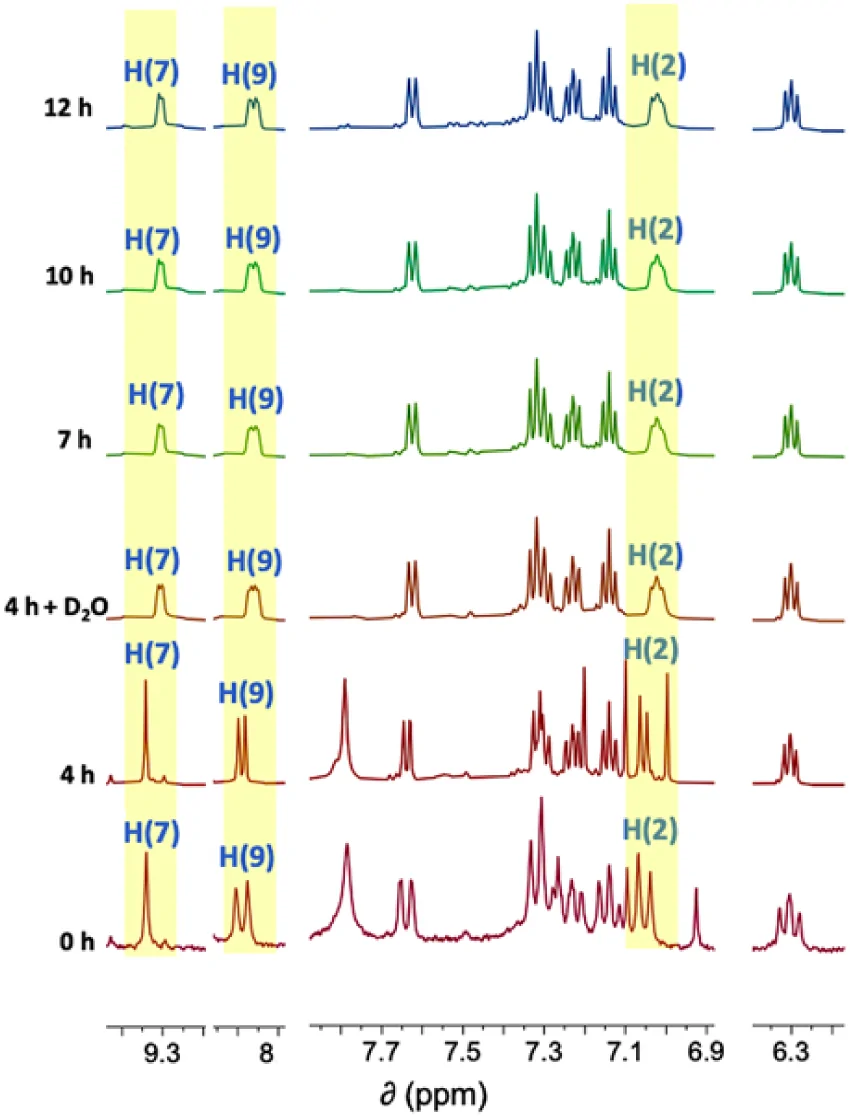
^1^H NMR stability test of **[PtCl­(L^1^**)] in DMSO-*d*_6_.

Following our methodology, we evaluated the stability of **[PtCl­(L** ^**1**^)] by UV–Vis spectroscopy in aqueous and biological buffer solutions containing 1% (v/v) DMSO, using the same range of concentrations as before (Figures S47–S51). The UV–Vis spectra recorded in Tris–HCl buffer at 20 μM displayed the two expected spin-forbidden transitions,^46^ and the band at 480 nm exhibited a hypsochromic shift. This observation suggests that a ligand-exchange process may be occurring at the metal center. In contrast, when the experiment was conducted in 0.9% (w/v) NaCl solution with 1% (v/v) DMSO, the spectral profile remained unchanged; however, a gradual decrease in absorbance was detected (Figure S50) as solids formed in the solution, ultimately resulting in complete precipitation of the complex. These findings indicate that high chloride concentrations may hinder chloride substitution, thereby preventing the formation of aqua species. The behavior of **[PtCl­(L** ^**1**^)] in 20 mM phosphate buffer (pH 7.4) could not be assessed, as all samples showed progressive precipitation.

To corroborate these hypotheses, a conductivity test was conducted (Figure S52). The molar conductivity of a 0.001 mol L^–1^ solution of **[PtCl­(L** ^**1**^)] in DMF and DMSO remained in the range of 12–13 S cm^2^ mol^–1^ over 24 h, which is indicative of neutral species.^50,51^ When using 1% (v/v) of DMSO–water solution with the same compound’s concentration, the molar conductivity was 130 S cm^2^ mol^–1^ and remained constant over the same period. While conductivity could not be measured in pure water, the values obtained in a 1% (v/v) DMSO/aqueous solution were consistent with a 1:1 electrolyte (within reported values in aqueous media).^52^ These results confirmed our hypotheses that **[PtCl­(L** ^**1**^ **)]** undergoes aquation under the experimental conditions.

### Cytotoxicity and Cellular Uptake

Given the marked differences observed in aqueous stability in both platinum complexes, we next investigated whether these chemical features translated into distinct cellular responses in the GC models. We first assayed the cytotoxicity of the complexes in GC cell lines AGS, MKN28, and MKN45 (). We included GES-1 cells to compare the toxicity in a nontumoral cell line. The free ligands, **L** ^**1**^ and **L** ^**1**^ **H**, did not show significant toxicity, while the conjugation with the Pt­(II) exhibited cytotoxicity regardless of the cell line, being more active with **[PtCl­(L** ^**1**^ **H)]­Cl** than **[PtCl­(L** ^**1**^)] (Table 3 and ). Furthermore, **[PtCl­(L** ^**1**^ **H)]­Cl** elicited more antiproliferative effects in comparison to cisplatin and oxaliplatin, making it the most promising candidate among all of them. Notably, despite the higher intrinsic chemical reactivity traditionally associated with Schiff base ligands, the reduced complex **[PtCl­(L** ^**1**^ **H)]­Cl** consistently exhibited superior antiproliferative activity across all tested GC cell lines. This apparent paradox can be rationalized by considering that biological activity depends not only on reactivity but also on chemical stability, solubility, and persistence of the active species under physiological conditions.

**Table 3:** IC_50_ Concentration (Mean Value ± SD) μM at 48 h. N = 3

	AGS	MKN28	MKN45	GES-1
**Cisplatin**	27.78 ± 1.53	20.37 ± 5.43	9.09 ± 1.34	2.00 ± 0.47
**Oxaliplatin**	2.57 ± 0.97	5.50 ± 2.93	3.39 ± 0.19	0.64 ± 0.19
**L** ^**1**^	>50	>50	>50	>50
**[PtCl(L** ^**1**^)]	21.77 ± 1.22	28.92 ± 4.65	20.69 ± 4.43	11.80 ± 1.27
**L** ^**1**^ **H**	>50	>50	>50	>50
**[PtCl(L** ^**1**^ **H)]Cl**	4.04 ± 1.09	3.59 ± 0.12	3.84 ± 1.19	3.35 ± 0.64

Following the cellular viability assays, the compounds were selected for further mechanistic evaluation to determine whether their biological effects were associated with differences in intracellular drug accumulation. Therefore, quantitative ICP-MS analysis was performed to assess the cellular uptake and subcellular distribution of the compounds. Consistent with previous reports showing that platinum intracellular distribution may vary depending on compound structure and experimental conditions, cisplatin in our experimental setting predominantly accumulated in the cytoplasmic fraction after 3 h of treatment,^53^ whereas both **[PtCl­(L** ^**1**^)] and **[PtCl­(L** ^**1**^ **H)]­Cl** exhibited higher accumulation in the nuclear compartment. Furthermore, calculation of the nuclear-to-cytoplasmic platinum ratio further highlighted these differences, showing a markedly higher nuclear enrichment for **[PtCl­(L** ^**1**^ **H)]­Cl** (14.33) compared to **[PtCl­(L** ^**1**^)] (2.65) and cisplatin (0.49) (Table 4). This preferential nuclear accumulation supports a distinct intracellular distribution pattern and may contribute to the enhanced biological activity observed for **[PtCl­(L** ^**1**^ **H)]­Cl**. Nonetheless, both complexes showed substantially higher platinum levels in both cellular fractions compared to cisplatin, suggesting more efficient cellular internalization in GC cells. Interestingly, intracellular platinum accumulation appeared to parallel the biological activity observed for the compounds, as **[PtCl­(L** ^**1**^ **H)]­Cl**, which displayed the strongest antiproliferative effects, also exhibited the highest degree of cellular uptake. Notably, this effect was particularly evident within the nuclear fraction, suggesting that the intracellular distribution may contribute to the enhanced biological activity of the compound. Previous studies on metallodrugs have shown that intracellular accumulation and subcellular localization are important determinants of biological activity, influencing target accessibility and cellular responses.^54–56^

**Table 4:** Subcellular Distribution of Platinum in MKN45 Cells after 3 h of 20 μM Treatment with the Complexes^a^

	Cytoplasm	Nucleus	Ratio Nucleus/Cytoplasm
**Control**	0.0056 ± 0.0015	0.0012 ± 0.0004	0.21
**Cisplatin**	0.8016 ± 0.0091	0.3940 ± 0.0021	0.49
**[PtCl(L** ^**1**^)]	2.9718 ± 0.0206	7.8773 ± 0.0638	2.65
**[PtCl(L** ^**1**^ **H)]Cl**	3.9810 ± 0.0178	57.0313 ± 0.2659	14.33

a The data are expressed as the mean values (±SD) of the metal (ppb) content in the nucleus or cytoplasm. N = 3

### Evaluation of DNA-Binding Potential of the Platinum Compounds

DNA is a critical molecular target for candidate antitumor metallodrugs.^57,58^ From a medicinal chemistry perspective, probing the interactions between these platinum complexes and DNA is essential for elucidating the mechanism of action underlying the target accumulation already demonstrated in this study.

pBR322 electrophoresis assay incubation of complexes is a useful tool to assess potential interactions with DNA. UV–vis interaction studies with CT-DNA were not possible due to the strong absorbance of our complexes, which overlaps with the main DNA band (260 nm). Free ligands **L** ^**1**^ and **L** ^**1**^ **H** were examined (Figure 8a, lanes 3–8), and their electrophoretic profiles remained unchanged relative to the DNA control (lane 2), indicating they do not induce alterations in plasmid topology. Cisplatin was employed as a positive control for covalent binding (Figure 8b, lanes 3–6). As reported, cisplatin caused a significant decrease in the rate of migration of the CC band of the pBR322 plasmid DNA and affected the mobilization rate of the OC form until comigration (r_i_ = 0.1).^59,60^

**Figure 8:**
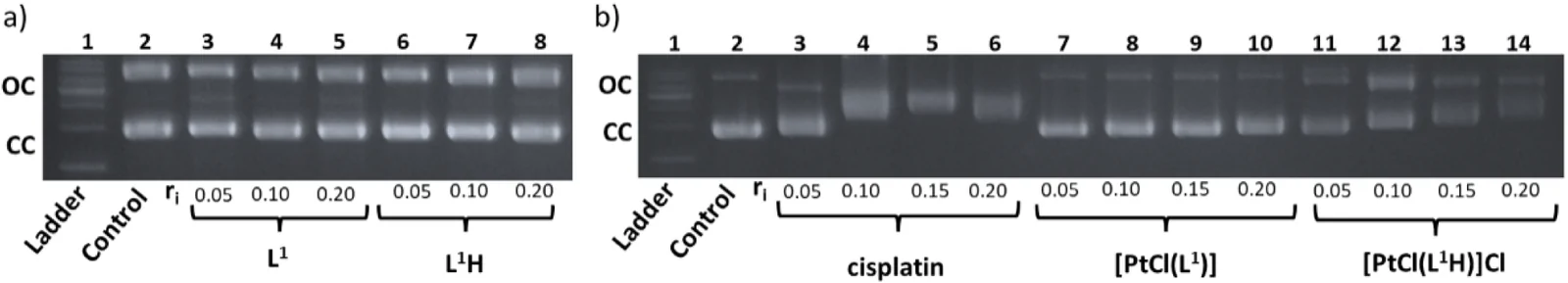
Gel electrophoresis assay with pBR322 at 0.0625 μg μL^–1^, incubated for 24 h with increasing concentrations of: **a) L^1^** (lanes 3–5) and **L^1^H** (lanes 6–8) and **b) cisplatin** (lanes 3–6), **[PtCl­(L^1^**)] (lanes 7–10), and **[PtCl­(L^1^H)]­Cl** (lanes 11–14). Lane 1: Ladder 1 kb; Lane 2: pBR322; 0.0625 μg μL^–1^ with water. Incubations at 37 °C with r_0_ values of 0.05, 0.10, 0.15, and 0.20 (r_0_ = [complex]/[DNA]).

**[PtCl­(L** ^**1**^)] produced almost no effect at r_i_ = 0.15, as the electrophoretic pattern was as the untreated control (Figure 8b, lanes 7–9). Only at a higher ratio (r_i_ = 0.20), the migration of the OC form was slightly affected (lane 10). On the other hand, **[PtCl­(L** ^**1**^ **H)]­Cl** (Figure 8b, lanes 11–14) exhibited a completely different profile. Although no comigration is observed (lanes 11–14), the CC is very affected, almost converging to the OC at r_i_ = 0.20, underlying a different DNA interaction in comparison to cisplatin.

As we can find variability in the literature regarding DNA binding, the determination of the mode of interaction between compounds and DNA requires additional studies.^61,62^ We decided to perform viscosity measurements as a complementary assay. The relative viscosity (η/η0) ^1/3^ was analyzed as a function of the 1/R ratio ([Compound]/[DNA]). Ethidium bromide (EtBr) was used as a positive intercalator of DNA, and cisplatin was used as the covalent control.

As expected, EtBr produced a progressive increase in viscosity, a characteristic response of intercalators, which insert between adjacent base pairs and elongate the DNA helix, resulting in measurable viscosity enhancement.^42^ Cisplatin, by contrast, caused a decrease in viscosity, consistent with its documented mechanism of forming cross-links.^63–65^ **[PtCl­(L** ^**1**^)] generated a constant curve, with no significant increases in viscosity even at the highest ratios. In contrast, **[PtCl­(L** ^**1**^ **H)]­Cl** produced a moderate decrease in the relative viscosity as its concentration increased. These results suggest a noncovalent and covalent interaction for **[PtCl­(L** ^**1**^)] and **[PtCl­(L** ^**1**^ **H)]­Cl**, respectively, since the effect observed for **[PtCl­(L** ^**1**^ **H)]­Cl** is parallel and like that of cisplatin, while **[PtCl­(L** ^**1**^)] maintains practically the same value as DNA alone (Figure 9).

**Figure 9:**
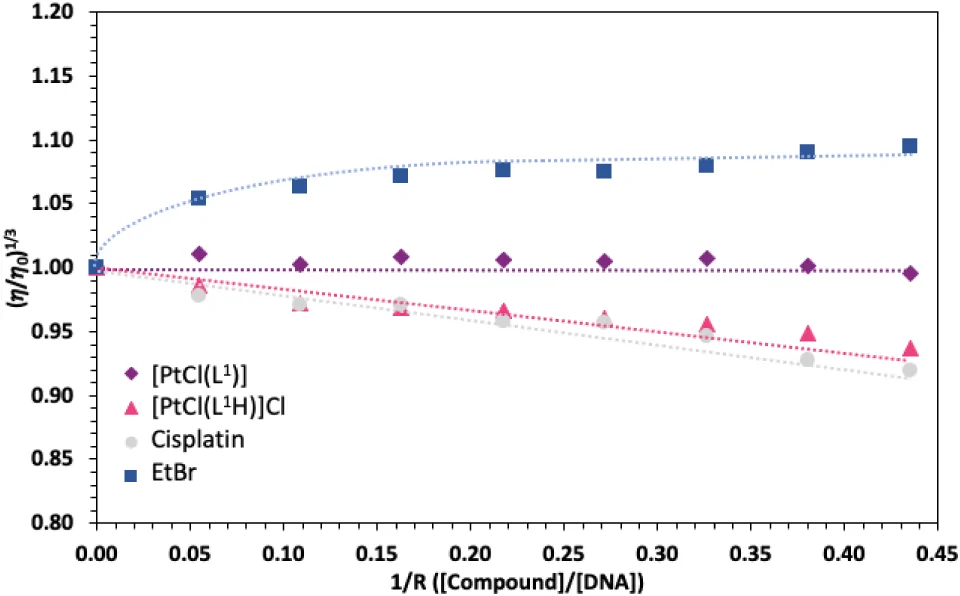
Effects of increasing amounts of EtBr (filled square), cisplatin (filled circle), **[PtCl­(L^1^**)] (filled diamond), and **[PtCl­(L^1^H)]­Cl** (filled triangle) on the viscosity of CT-DNA; [DNA] = 46 μM; [complexes] = 0, 2.5, 5.0, 7.5, 10.0, 12.5, 15.0, 17.5, and 20.0 μM, respectively.

### BSA Interaction Fluorescence Assays

The study of the interaction between antitumor compounds and serum albumin is of paramount importance, as albumin is the most abundant protein in blood and plays a key role in the transport, distribution, and bioavailability of drugs.^66,67^ Accordingly, the interaction of [Pt**Cl­(L** ^**1**^)] and [Pt**Cl­(L** ^**1**^ **H)]­Cl** with bovine serum albumin (BSA) was investigated by fluorescence spectroscopy.

BSA is commonly used as a model protein due to its intrinsic fluorescence, which mainly arises from tryptophan (Trp) residues, with minor contributions from tyrosine (Tyr) and phenylalanine (Phe).^67,68^ Following previously reported optimized conditions, excitation at λexc = 280 nm was employed, where the emission is dominated by Trp and Tyr residues. Emission spectra of BSA (Figure S54) were recorded upon the addition of increasing concentrations of the platinum complexes (up to 5 μM). The progressive addition of both platinum complexes resulted in a decrease in the fluorescence intensity of BSA, indicating interaction with the protein. Notably, **[PtCl­(L** ^**1**^)] induced a significantly stronger quenching effect compared to **[PtCl­(L** ^**1**^ **H)]­Cl**, suggesting a higher affinity of the neutral complex for the protein. Importantly, no significant shift in the emission maximum was observed, indicating that the local microenvironment of the tryptophan residues remains largely unchanged upon binding. This suggests that the interaction does not induce major conformational changes in the protein structure. To quantify the interaction, the fluorescence data were analyzed using the Stern–Volmer and double-logarithmic equations.^67^ The corresponding parameters are summarized in Table 5 and correspond to the average of three independent experiments.

**Table 5:** Binding Parameters Obtained from Stern–Volmer and Double-Logarithmic Analyses for the Interaction of Platinum Complexes with BSA

Complex	K_SV_ (M^–1^)	K_BIN_ (M^–1^)	n
**[PtCl(L** ^**1**^ **H)]Cl**	9.5 ×10^4^ ± 9.1×10^3^	1.2 ×10^6^ ± 8.6×10^3^	1.02
**[PtCl(L** ^**1**^)]	7.2 ×10^5^ ± 2.3 × 10^5^	5.4 ×10^9^ ± 4.7×10^9^	1.7

The higher K_SV_ and K_BIN_ values for **[PtCl­(L** ^**1**^)] indicate a more efficient quenching process compared to the cationic complex. In particular, the binding constant (K_BIN_) for **[PtCl­(L** ^**1**^)] is significantly larger than that of **[PtCl­(L** ^**1**^ **H)]­Cl**, suggesting a stronger interaction with BSA under the studied conditions. The values obtained for **[PtCl­(L** ^**1**^ **H)]**Cl fall within the range commonly reported for small molecule–albumin interactions, while the higher value observed for **[PtCl­(L** ^**1**^)] indicates a comparatively stronger affinity. The number of binding sites (n ≈ 1–2) suggests that both complexes interact with approximately one main binding site on BSA. Overall, these results indicate that both platinum compounds are capable of interacting with serum albumin, with **[PtCl­(L** ^**1**^)] showing a higher apparent affinity than its cationic analogue.

### Cell Cycle, DNA Damage Response, and Apoptosis

Cell cycle profiles were assessed in MKN45 cells following treatment with the complexes and their respective ligands, following treatment with the complexes and the ligands in MKN45 cells. While cisplatin produced a strong arrest in the S phase, **[PtCl­(L** ^**1**^)] and **[PtCl­(L** ^**1**^ **H)]­Cl** exhibited an accumulation of cells in G2/M, correlating with DNA structural perturbations. None of the ligands affected the cell cycle distribution at the concentrations studied (Figure 10, Figure S55 and Table S2).

**Figure 10:**
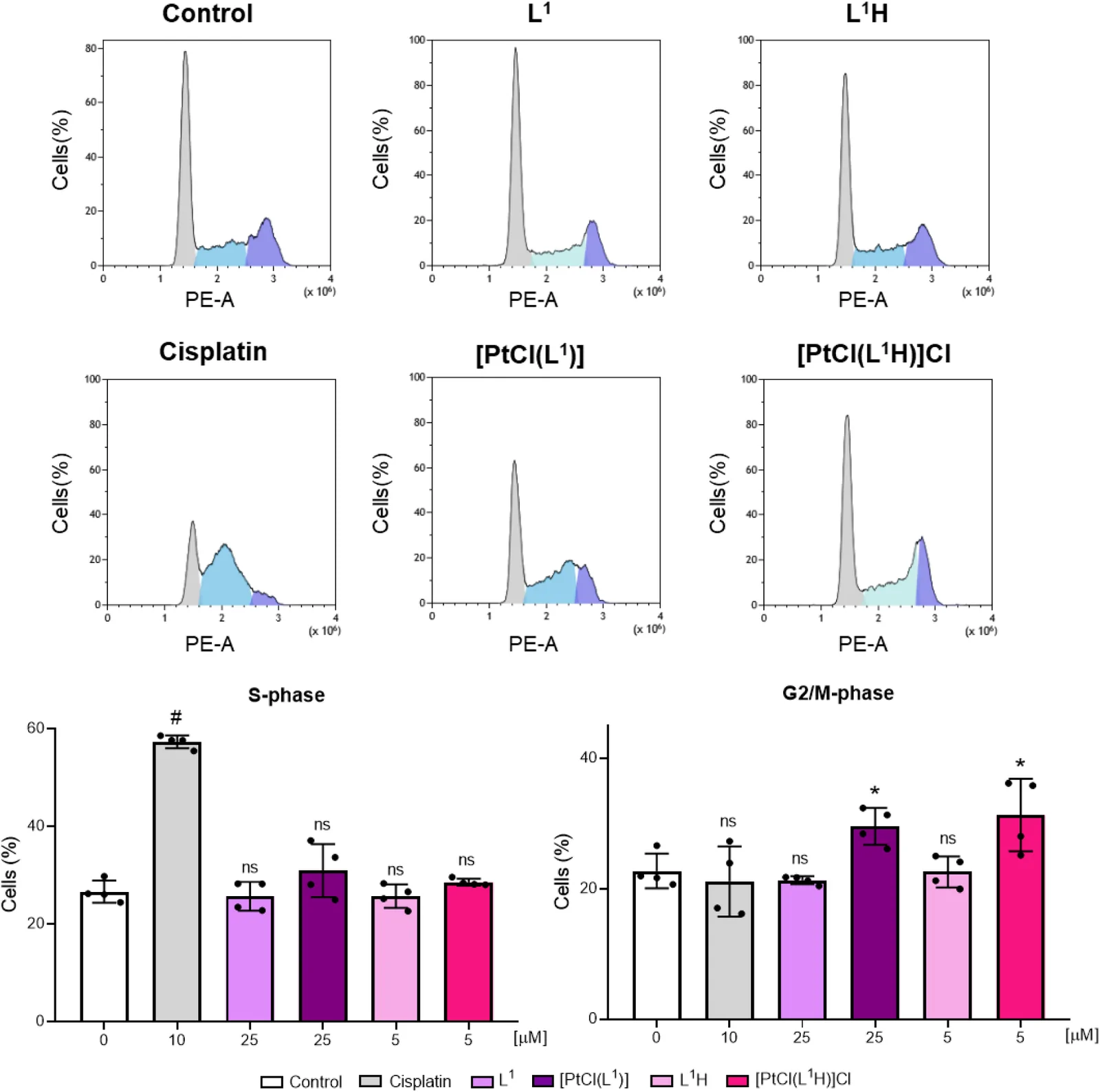
**[PtCl­(L^1^**)] and **[PtCl­(L^1^H)]­Cl** modulate the cell cycle. MKN45 cells were treated with **L^1^**, **[PtCl­(L^1^**)] (25 μM), and **L^1^H** and **[PtCl­(L^1^H)]­Cl** (5 μM). Cisplatin was included as a standard drug (10 μM). Twenty-four hours after treatment, cells were fixed and stained with propidium iodide. Graphs show representative cell cycle profiles after treatments (upper panel) and the percentage of cells in the S and G2/M phases (lower panel). Statistical significance was evaluated by an unpaired Student’s 2-tailed *t*-test (ns: not significant, *p < 0.05, #*p* < 0.0001) compared to untreated cells (Control). *N* = 4.

Replication stress caused by DNA lesions typically activates the ATR–CHK1 axis, leading to checkpoint activation and cell cycle delay. Accordingly, we examined the activation status of key DNA damage response (DDR) components following treatment with the platinum complexes. We studied the phosphorylation status of CHK1 and p53 in MKN45 cells. Notably, while cisplatin strongly activated p53, both platinum complexes activated CHK1 without significantly inducing p53 phosphorylation, suggesting a p53-independent DDR, increasing our interest in these drugs because nearly 50% of solid tumors have p53 mutated^69^ (Figure 11).

**Figure 11:**
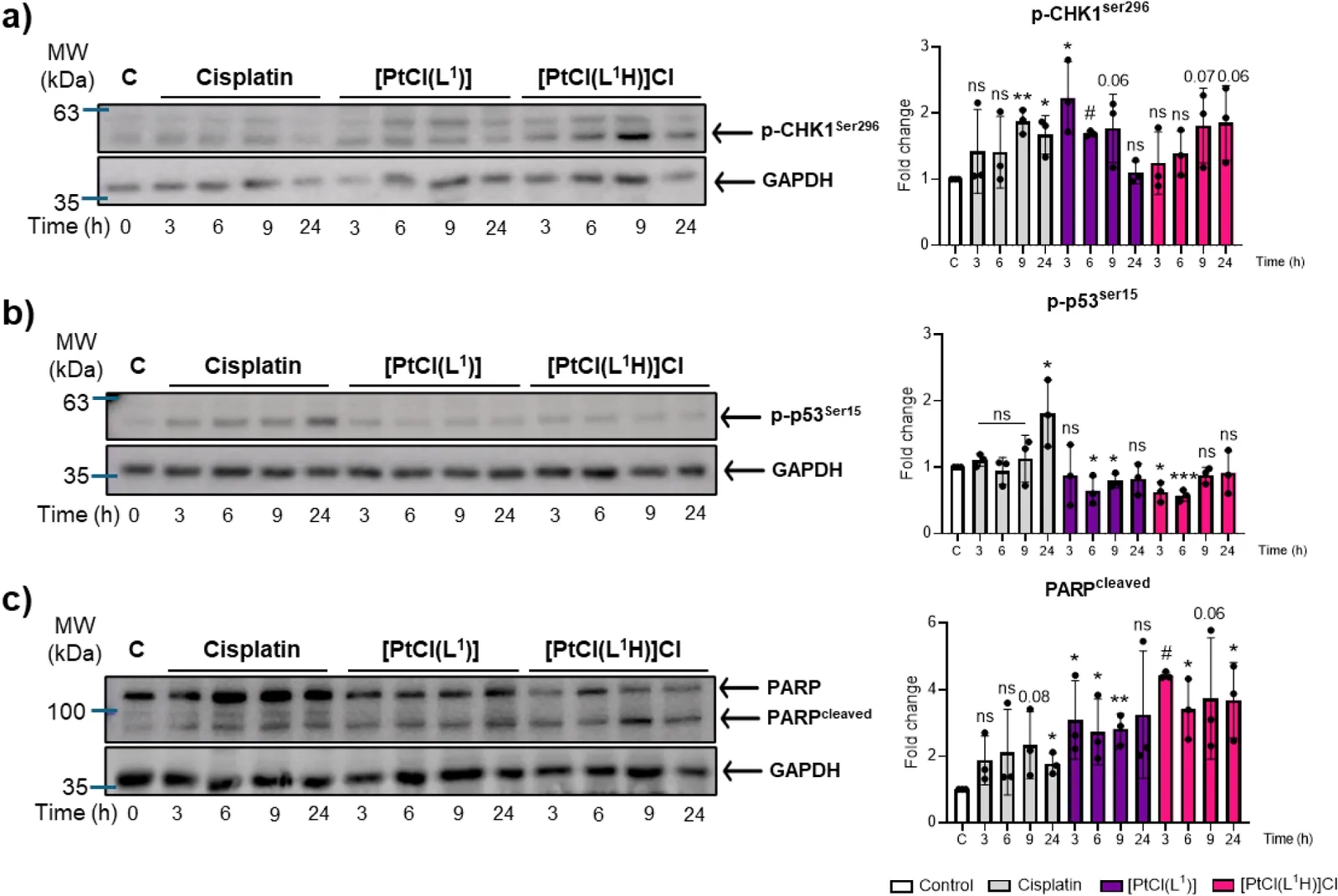
**[PtCl­(L^1^**)] and **[PtCl­(L^1^H)]­Cl** activate the DDR. Western blot analysis in MKN45 cells after treatment with cisplatin (10 μM), **[PtCl­(L^1^**)] (25 μM), and **[PtCl­(L^1^H)]­Cl** (10 μM) at different times (3, 6, 9, and 24 h): **a)** phospho-CHK1­(Ser296); **b)** phospho-p53­(Ser15); **c)** PARP (cleaved). Graphs show each protein expression normalized with GAPDH or α-tubulin, used as endogenous controls: Control cells (C, white bars), cisplatin (gray), **[PtCl­(L^1^**)] (purple), and **[PtCl­(L^1^H)]­Cl** (pink). Statistical significance was evaluated with an unpaired Student’s 2-tailed *t*-test (ns: not significant, *p < 0.05, ** *p* < 0.01, ***p < 0.001, #*p* < 0.0001) compared to untreated cells (C: Control), set as 1.0. *N* = 3.

CHK1 activation reflects the engagement of the DDR pathway in response to replication stress via ATR.^70^ We examined, by Western blotting, the cleavage of PARP in MKN45 cells. We detected PARP cleaved 24 h after the treatment, confirming apoptosis activation. Furthermore, we assessed the apoptotic expression of BAX, a pro-apoptotic protein,^71^ and MCL1, an antiapoptotic protein, both involved in mitochondrial apoptosis. We observed that cisplatin increased Bax mainly at 9 and 24 h, while **[PtCl­(L** ^**1**^)] increased it at 24 h, and **[PtCl­(L** ^**1**^ **H)]­Cl** did it earlier (at 3 h). In contrast, MCL1 decreased at 24 h, confirming its pro-apoptotic activity in MKN45 cells (Figure 12). The early induction of BAX and the concomitant decrease in MCL1 indicate the engagement of the intrinsic apoptotic pathway downstream of DNA damage.

**Figure 12:**
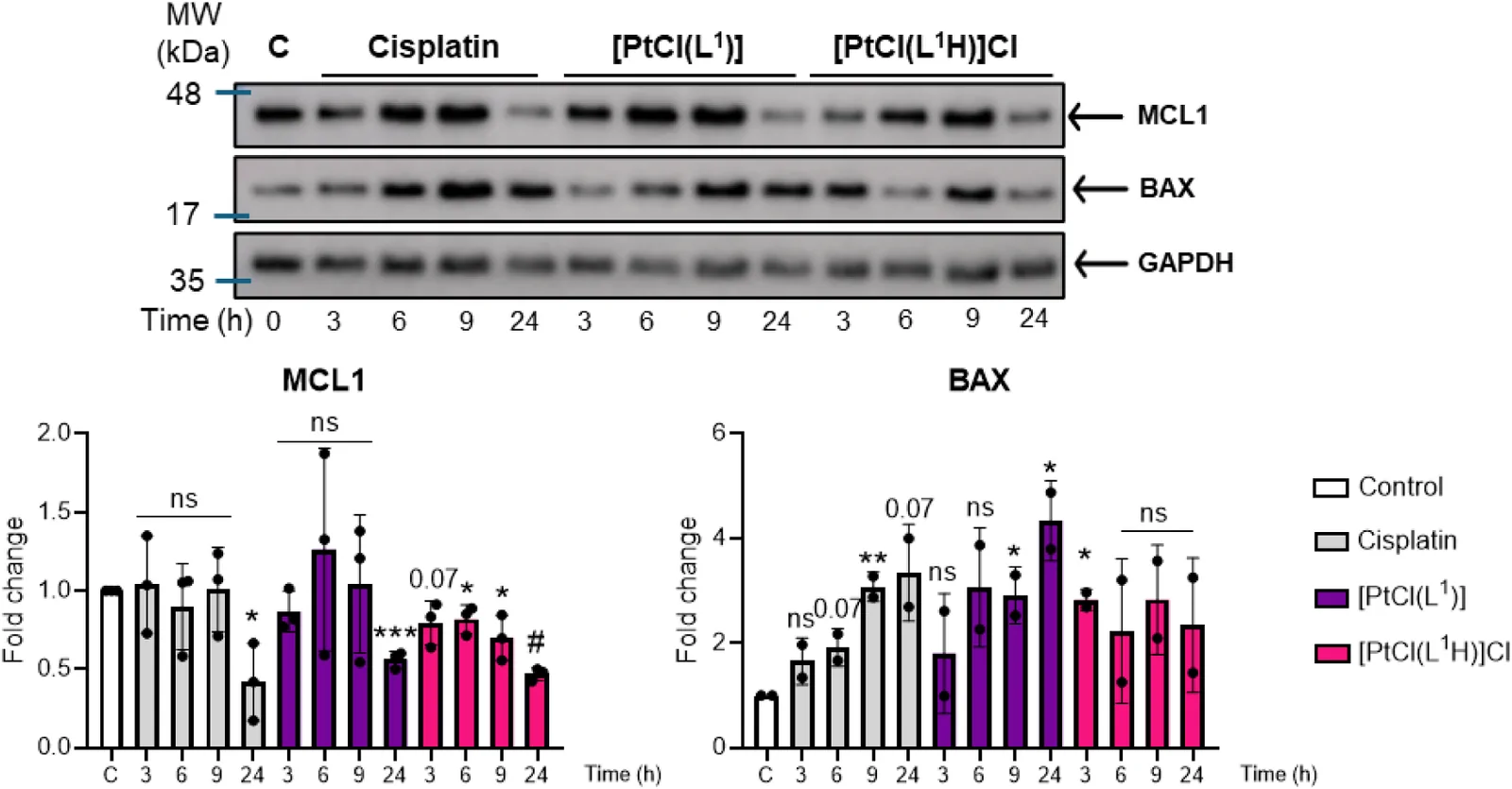
Complexes induce intrinsic apoptosis. Western blot analysis of MCL1 (*N* = 3) and BAX (*N* = 2) proteins in MKN45 cells after treatment with cisplatin (10 μM), **[PtCl­(L^1^**)] (25 μM), and **[PtCl­(L^1^H)]­Cl** (10 μM) at different times (3, 6, 9, and 24 h). Graphs show each protein expression normalized with GAPDH or α-tubulin, used as endogenous controls: Control cells (C, white bars), cisplatin (gray), **[PtCl­(L^1^**)] (purple), and **[PtCl­(L^1^H)]­Cl** (pink). Statistical significance was evaluated with an unpaired Student’s 2-tailed *t*-test (ns: not significant, *p < 0.05, ** *p* < 0.01, ***p < 0.001, #*p* < 0.0001) compared to untreated cells (C: Control), set as 1.0. *N* = 3.

The stronger and more sustained cytotoxic effect of **[PtCl­(L** ^**1**^ **H)]­Cl** was accompanied by pronounced activation of the DDR and apoptotic signaling, indicating that the improved chemical stability of the reduced complex enables a more effective engagement of canonical platinum-induced cell death pathways.

### Mitochondrial Dysfunction and ROS Accumulation

Given the observed apoptotic effects, we next examined mitochondrial function to assess whether these compounds interfere with its activity. In addition to nuclear DNA, platinum-based compounds have been reported to affect mitochondrial function, either because of DNA damage or through direct mitochondrial targeting. This prompted us to explore whether mitochondrial dysfunction and ROS generation contributed to the cytotoxic effects observed.^72–74^ First, we measured mitochondrial mass and mitochondrial membrane potential (ΔΨm) in MKN45 cells exposed to cisplatin, **[PtCl­(L** ^**1**^)] and **[PtCl­(L** ^**1**^ **H)]­Cl**. Mitochondrial mass and ΔΨm were measured by using MitoTracker DeepRed and MitoTracker Red CMXRos, respectively, and were used to calculate the ΔΨm/Mit. Mass ratio (Figure 13a), a way to normalize the membrane potential respective to the total Mit. Mass. Our results indicated that all the complexes decrease the ΔΨm/Mit. Mass ratio is an indicator of dysfunctional/damaged mitochondria, with the most potent effect observed with **[PtCl­(L** ^**1**^ **H)]­Cl** (>50% reduction). Mitochondrial dysfunction often results in enhanced ROS production, primarily due to impairments in the electron transport chain, where O_2_ acts as the terminal electron acceptor. We then evaluated mitochondrial O_2_^**·**^ generation after 24 h (Figure 13b). Although cisplatin and **[PtCl­(L** ^**1**^)] significantly increased O_2_^**·**–^ levels, **[PtCl­(L** ^**1**^ **H)]­Cl** produced a huge effect on ROS generation. Accordingly, the three complexes induced p38 phosphorylation (fold > 2.0) closely related to oxidative stress.^75^ Finally, we wondered if their cytotoxic effect could be linked to oxidative damage (Figure 13c). To that end, we evaluated cell viability in the presence or absence of a nontoxic concentration (0.5 mM) of N-acetylcysteine (NAC), a ROS scavenger previously used in different publications for a similar purpose.^6,76,77^ Interestingly, while NAC treatment did not affect cisplatin and **[PtCl­(L** ^**1**^)] toxicity, it produced a clear protective effect after **[PtCl­(L** ^**1**^ **H)]­Cl** treatment, indicating that the antiproliferative effect of this compound is mostly due to oxidation damage, which can be rescued by scavenging ROS (Figure 13d). The observed mitochondrial depolarization and ROS accumulation are consistent with a DNA damage-driven apoptotic program, reinforcing the mechanistic link between DNA interaction and cell death.

**Figure 13:**
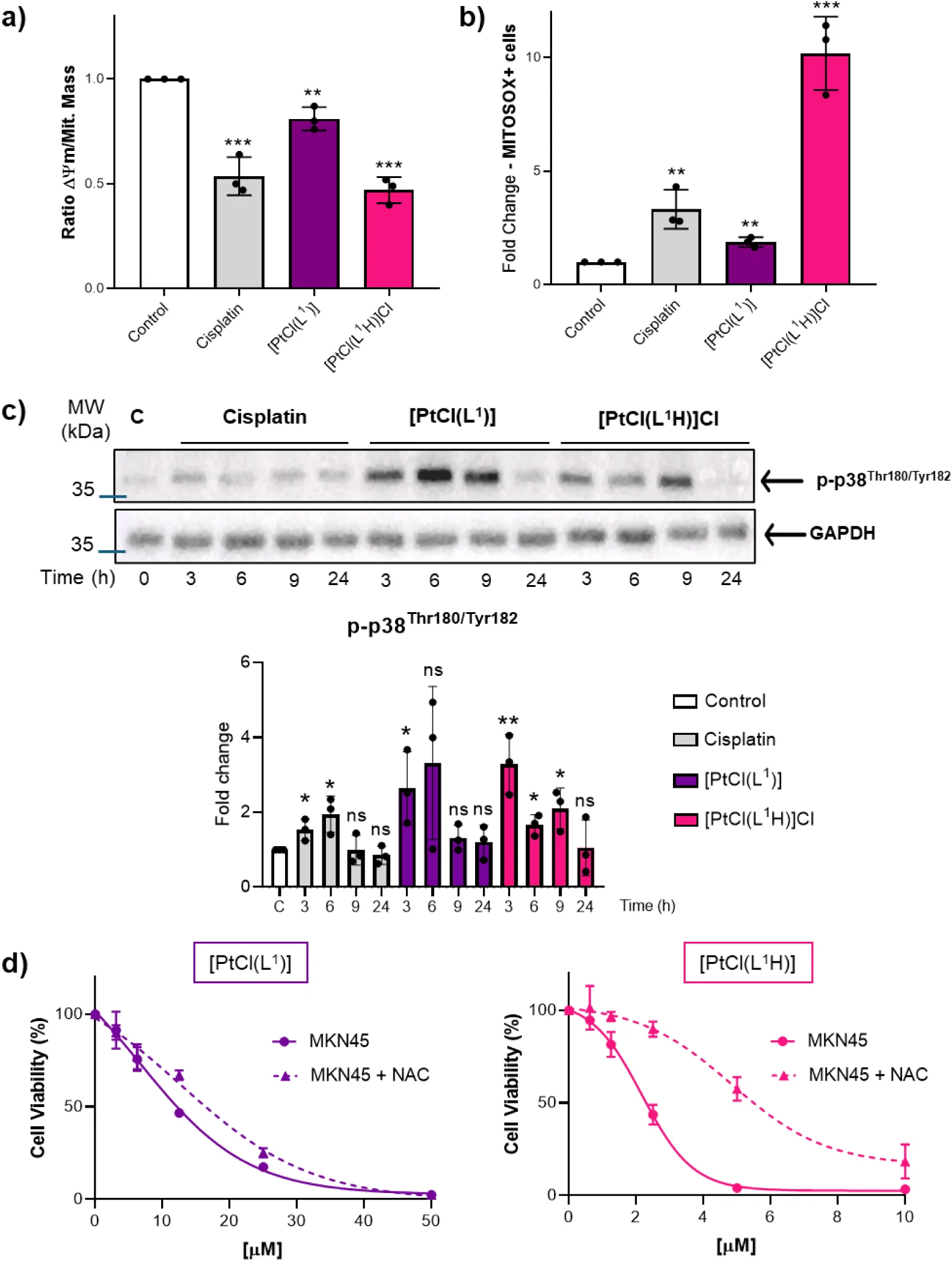
**[PtCl­(L^1^H)]­Cl** induces cell death via oxidative damage. **a)** Mean fold-change ± SD of the ratio of ΔΨm probe (CMX-ROS)/Mitochondrial mass probe (MTDR) as a measurement to evaluate mitochondrial functionality, and **b)** mean fold-change ± SD of MitoSOX probe (mitochondrial superoxide indicator) in MKN45 cells treated with cisplatin (10 μM), **[PtCl­(L^1^**)] (25 μM), and **[PtCl­(L^1^H)]­Cl** (10 μM) for 24 h. **c)** Western blot analysis of phospho-p38 in MKN45 cells after treatment with cisplatin (10 μM), **[PtCl­(L^1^**)] (25 μM), and **[PtCl­(L^1^H)]­Cl** (10 μM) at different times (3, 6, 9, and 24 h). In all experiments statistical significance was evaluated with an unpaired Student’s 2-tailed *t*-test (ns: not significant, *p < 0.05, **p < 0.01, ***p < 0.001) compared to untreated cells (C: Control) set as 1.0. *N* = 3. **d)** Effects of the complexes on cell viability in MKN45 cells in the absence (solid line) or presence (dashed line) of N-acetylcysteine (NAC) 0.5 mM. Cells were treated with increasing concentrations of **[PtCl­(L^1^**)] (left graph) and **[PtCl­(L^1^H)]­Cl** (right graph). Graphs show the mean percentage ± SD of viable cells after 48 h of treatment. *N* = 3.

### Effect on 3D Models: CSC-Enriched Tumorospheres

CSCs are intrinsically resistant to conventional therapies such as cisplatin, constituting a common challenge in the clinic that often leads to tumor relapses.^6,78^ Thus, we further evaluated the potential of **[PtCl­(L** ^**1**^)] and **[PtCl­(L** ^**1**^ **H)]­Cl** to eliminate GCSCs by evaluating the viability of CSC-enriched cultures (spheroids) of MKN45 cells. GCSCs were resistant to cisplatin and also oxaliplatin (RF: 2.3 and 2.0, respectively) compared to their adherent counterparts. Strikingly, both complexes, **[PtCl­(L** ^**1**^)] and **[PtCl­(L** ^**1**^ **H)]­Cl**, maintained similar cytotoxicity in both adherent and GCSC populations. To gain further information about this trend, we translated our results to another tumor model based on pancreatic ductal adenocarcinoma (PDAC) (PANC1 cells). The results were in agreement with the previous GCSCs model, obtaining a huge resistance in PANC1 CSCs after cisplatin and oxaliplatin treatments (RF of 6.0 and 5.3) but not to **[PtCl­(L** ^**1**^)] nor **[PtCl­(L** ^**1**^ **H)]­Cl**. We suggest that **[PtCl­(L** ^**1**^ **H)]­Cl** and to a lesser extent **[PtCl­(L** ^**1**^)], retain their efficacy against gastrointestinal CSCs (Figure 14 and Table 6).

**Table 6:** IC_50_ Concentration (Mean Value ± SD) μM, 72 h, in CSC-Enriched Tumorospheres. Resistant Factor is Indicated in Red. N = 3

	MKN45	MKN45^CSCs^	PANC1	PANC1^CSCs^
**Cisplatin**	11.16 ± 0.72	25.71 ± 1.96 (**2.3**)	6.20 ± 0.62	37.39 ± 3.45 (**6.0**)
**Oxaliplatin**	18.36 ± 0.34	35.89 ± 0.24 (**2.0**)	8.19 ± 3.14	43.02 ± 5.84 (**5.3**)
**[PtCl(L** ^**1**^)]	30.00 ± 3.25	33.58 ± 1.57 (**1.1**)	29.22 ± 3.75	38.25 ± 3.68 (**1.3**)
**[PtCl(L** ^**1**^ **H)]Cl**	10.84 ± 0.28	12.20 ± 1.91 (**1.1**)	4.86 ± 0.33	4.68 ± 0.88 (**1.0**)

**Figure 14:**
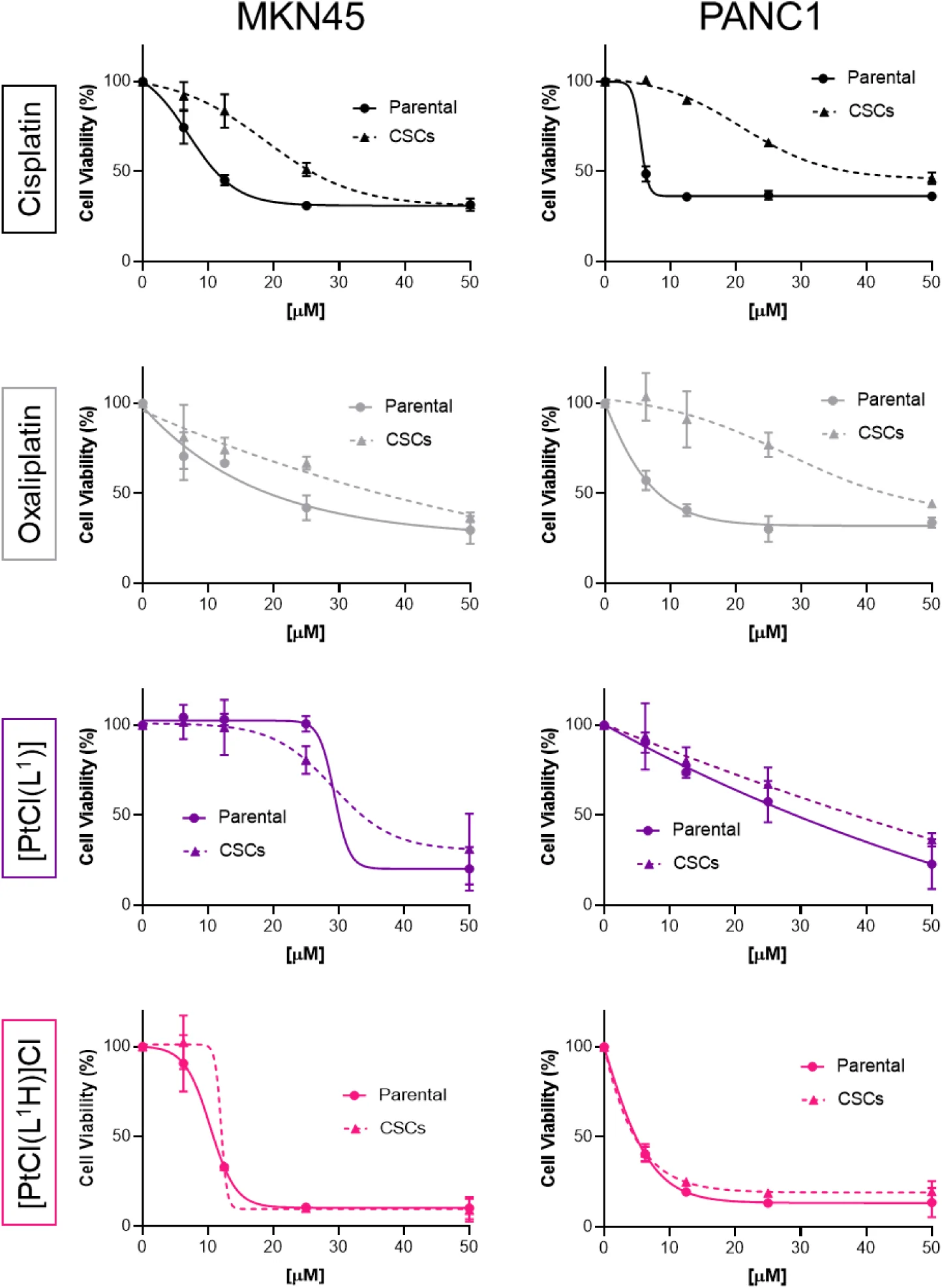
**[PtCl­(L^1^**)] and **[PtCl­(L^1^H)]­Cl** are active against CSCs. Cell viability was quantified using an MTS assay after 72 h of treatment with cisplatin, oxaliplatin, **[PtCl­(L^1^**)] and **[PtCl­(L^1^H)]­Cl** (0–50 μM) in MKN45 (left panel) and PANC1 (right panel) cell lines, cultured in adherence (parental cells) or as spheres (CSCs). Data represent the mean percentage ± SD values obtained in three experiments performed in quadruplicate.

In line with the above results, **[PtCl­(L** ^**1**^ **H)]­Cl** pretreatment of adherent cultures led to a marked reduction in their capacity to form clones and first-generation spheres, resulting in a significant reduction in the number of resulting colonies and tumorspheres in MKN45 cells (Figure 15). On the other hand, **[PtCl­(L** ^**1**^)] only affected sphere formation without interfering with the clonogenic capacity of MKN45 cells. None of the ligands produced any significant effect in terms of clonogenicity or self-renewal.

**Figure 15:**
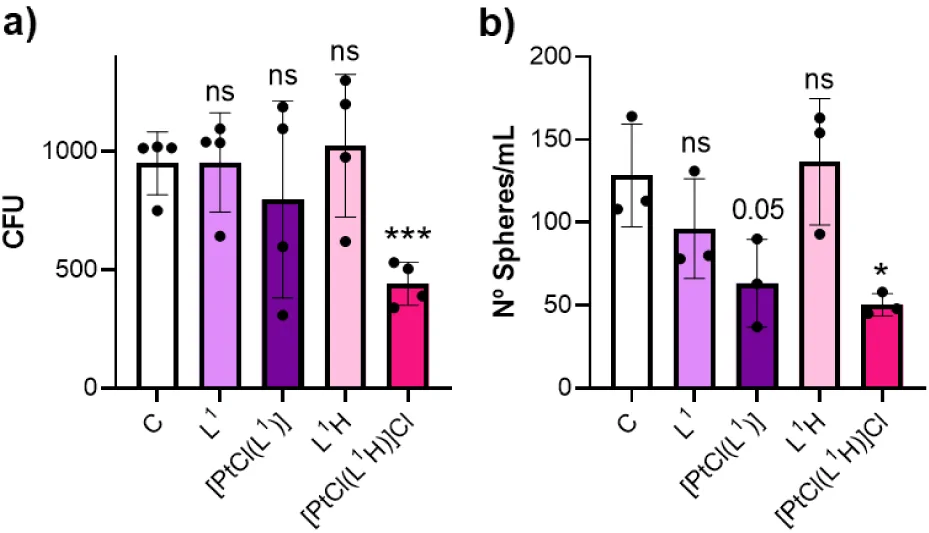
**[PtCl­(L^1^H)]­Cl** reduces cell clonogenicity and sphere formation. **a)** Colony formation assay of MKN45 cells treated with **L^1^**, **[PtCl­(L^1^**)] (25 μM), and **L^1^H** and **[PtCl­(L^1^H)]­Cl** (10 μM) for 24 h, seeded for 7 days, and stained with crystal violet for absorbance measurement. Data represent the mean ± SD values of Colony Forming Units (CFU). **b)** MKN45 cells were treated with **L^1^**, **[PtCl­(L^1^**)] (25 μM), and **L^1^H** and **[PtCl­(L^1^H)]­Cl** (10 μM) for 24 h prior to being seeded in MW24 ULA and incubated for 1 week. The number of spheres was counted per well in each condition. Data represent the mean ± SD values. Statistical significance was evaluated by one-way ANOVA with Dunnett’s posttest (ns: not significant, *p < 0.05) compared to untreated cells (C: [MS1] [JM2] Control). *N* = 4.

While numerous studies report novel metal-based complexes with activity against CSCs, only a limited number have examined the mechanisms driving their effects in this subpopulation.^79–83^ To gain deeper insight into the mechanism of our model, we first aimed to validate the activation of DDR in the CSC model. We observed an increase in CHK1 autophosphorylation after oxaliplatin and **[PtCl­(L** ^**1**^ **H)]­Cl** treatment. The lack of p53 activation by **PtCl­(L** ^**1**^)] and **[PtCl­(L** ^**1**^ **H)]­Cl** was maintained in this model, in contrast to oxaliplatin. On the other hand, only **[PtCl­(L** ^**1**^ **H)]­Cl** significantly induced p38 phosphorylation (Figure 16A). Additionally, PARP cleavage was detected with all of the complexes in MKN45 CSCs (Figure 16b). All these results together indicate engagement of a DNA damage-associated stress response, followed by caspase-dependent PARP cleavage.

**Figure 16:**
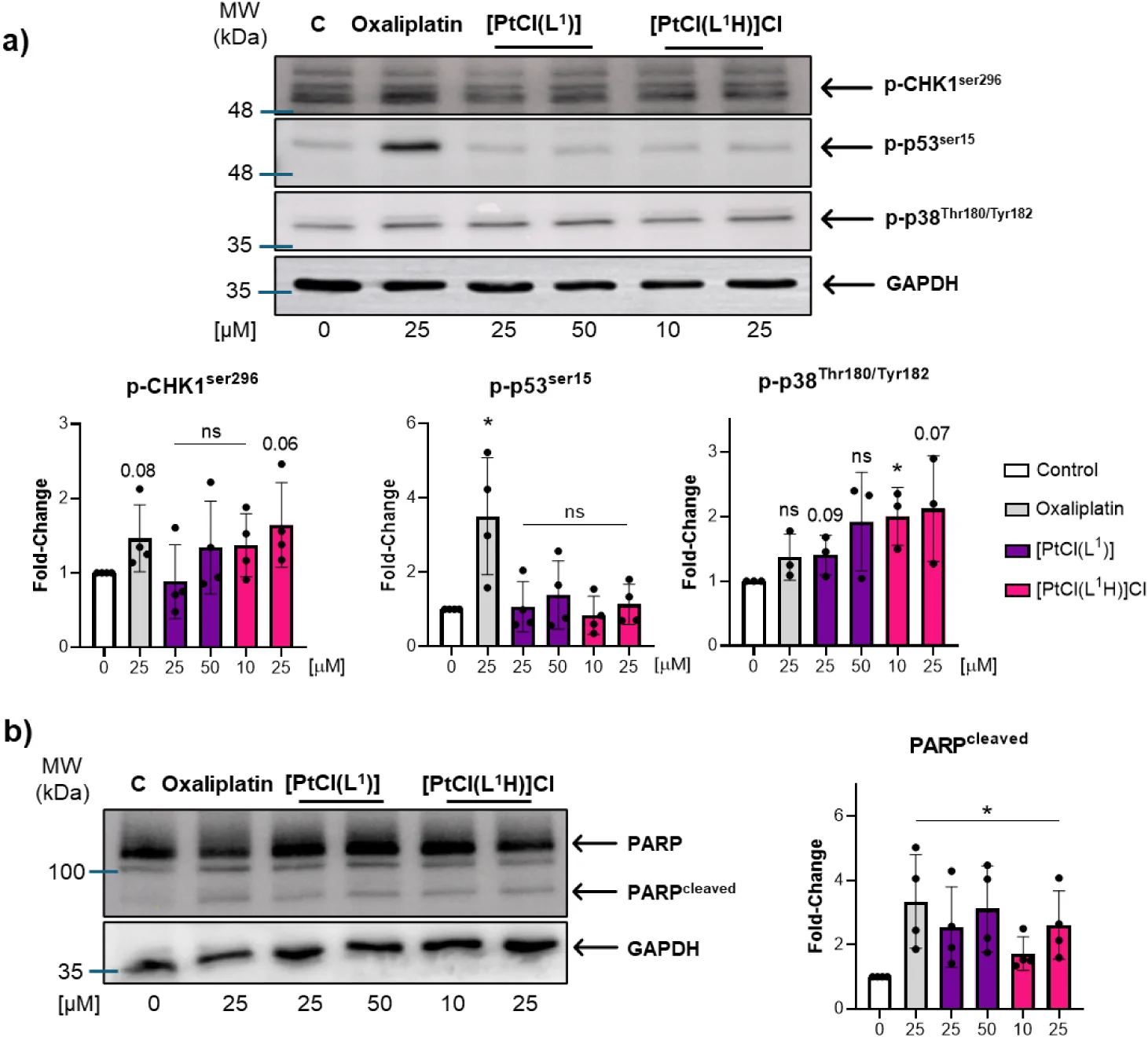
**[PtCl­(L^1^H)]**Cl activates the DDR and p38 MAPK pathway. Western blot analysis in MKN45 CSCs after treatment with oxaliplatin (25 μM), **[PtCl­(L^1^**)] (25 and 50 μM), and **[PtCl­(L^1^H)]­Cl** (10 and 25 μM) for 24 h: **a)** phospho-CHK1­(Ser296), phospho-p53­(Ser15), and phospho-p38­(Thr180/Tyr182); **b)** PARP (cleaved). Graphs show each protein expression normalized with GAPDH used as an endogenous control: Control cells (C, white bars), oxaliplatin (gray), **[PtCl­(L^1^**)] (purple), and **[PtCl­(L^1^H)]­Cl** (pink). Statistical significance was evaluated with Student’s 2-tailed *t*-test (ns: not significant, *p < 0.05) compared to untreated cells (C: Control) set as 1.0. *N* ≥ 3.

Finally, we evaluated several markers related to CSC maintenance and self-renewal: Notch1 (cleaved), phosphorylated STAT3 (at Tyr705), and OCT3/4 (pluripotency marker).^84–86^ The cleavage (and thus the activation) of Notch1 was abolished with **[PtCl­(L** ^**1**^ **H)]­Cl**, while oxaliplatin and **[PtCl­(L** ^**1**^)] did not interfere with that signaling network (Figure 17). Furthermore, we demonstrated a clear inhibition of STAT3 phosphorylation at Tyr705 after **[PtCl­(L** ^**1**^ **H)]­Cl** treatment, which was even significantly higher than the slight reduction produced by oxaliplatin, and in contrast to **[PtCl­(L** ^**1**^)] which did not produce any effect. This result, together with the absence of cleaved Notch1, is of extreme importance because STAT3 is also mainly involved in the negative regulation of immune response, cell growth, differentiation and apoptosis, and tumor occurrence and metastasis.^87,88^ To conclude and validate the functional tumorosphere formation assays, we evaluated OCT3/4 protein levels by Western blot. Although no changes were observed in Notch1 and phospho-STAT3, **[PtCl­(L** ^**1**^)] reduced OCT3/4 levels at a concentration of 50 μM, suggesting that the observed effects and its anti-CSC activity appear to be independent of the Notch and JAK/STAT pathways (at least for the selected markers). Similarly, **[PtCl­(L** ^**1**^ **H)]­Cl** also decreased OCT3/4 levels, a phenomenon that was not observed with oxaliplatin (Figure 17), emerging as a novel Pt­(II) agent capable of effectively targeting and eliminating CSCs by interfering with self-renewal and pluripotency.

**Figure 17:**
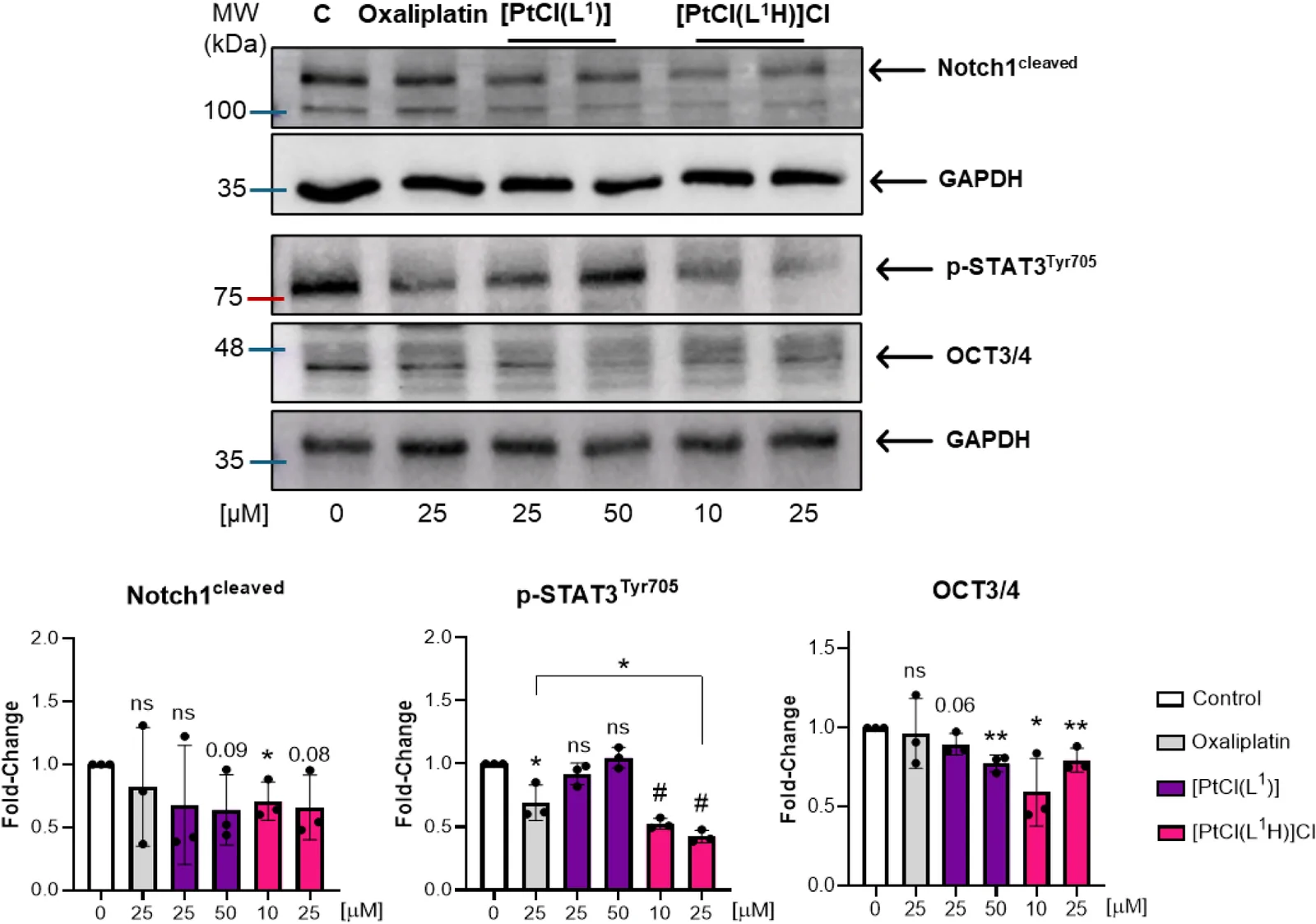
**[PtCl­(L^1^H)]­Cl** targets self-renewal and pluripotency in GCSCs. Western blot analysis in MKN45 CSCs after treatment with oxaliplatin (25 μM), **[PtCl­(L^1^**)] (25 and 50 μM), and **[PtCl­(L^1^H)]­Cl** (10 and 25 μM) for 24 h of Notch1 (cleaved, NICD), phospho-STAT3­(Tyr705), and OCT3/4 proteins. Graphs show each protein expression normalized with GAPDH used as the endogenous control: Control cells (C, white bars), oxaliplatin (gray), **[PtCl­(L^1^**)] (purple), and **[PtCl­(L^1^H)]­Cl** (pink). Statistical significance was evaluated with an unpaired Student’s 2-tailed *t*-test (ns: not significant, *p < 0.05, **p < 0.01, #*p* < 0.0001) compared to untreated cells (C: Control) set as 1.0. *N* = 3.

### Transcriptomic Profiling and Functional Studies

Based on these previous and encouraging findings with the **[PtCl­(L** ^**1**^ **H)]­Cl** complex, we aimed to further investigate its molecular mechanism of action. To this end, we defined an optimal concentration (5 μM) that balances therapeutic activity with cell viability. This concentration was used to treat cells for 24 h prior to RNA sequencing (RNA-seq). Three gastrointestinal cell lines were selected: MKN-28 (GC), MKN-45 (GC), and PANC1 (PDAC). RNA-seq analysis was performed for each cell line, followed by differential expression analysis comparing untreated and **[PtCl­(L1H)]­Cl-**treated samples. Differential expression analysis revealed a clear transcriptional impact associated with **[PtCl­(L** ^**1**^ **H)]­Cl** treatment, as visualized by volcano plots showing multiple significantly upregulated and downregulated genes according to the selected fold-change and statistical significance thresholds (Figure 18a). These results indicate that **[PtCl­(L1H)]­Cl** induces broad transcriptional reprogramming in gastrointestinal cancer cells, affecting gene subsets with potential functional relevance. Using the resulting gene expression profiles, expressed in transcripts per million of reads, we performed a Gene Set Enrichment Analysis (GSEA) to compare untreated and **[PtCl­(L** ^**1**^ **H)]­Cl**-treated samples. This analysis, performed across the three cell lines using the Hallmark gene set collection and the Signal-to-Noise metric, identified a significant enrichment of gene sets associated with DNA damage, apoptosis, and ROS following **[PtCl­(L** ^**1**^ **H)]­Cl** treatment (Figure 18b and c), consistent with our previous findings. Interestingly, epithelial-to-mesenchymal transition (EMT) was identified as the main negatively enriched pathway upon **[PtCl­(L** ^**1**^ **H)]­Cl** treatment (Figure 18b and c). EMT (and mesenchymal-to-epithelial transition) encompasses the dynamic processes through which cells transition between epithelial and mesenchymal phenotypes, playing crucial roles not only in normal embryonic development but also in tumor progression and metastatic dissemination.^89^ Importantly, the downregulation of EMT by therapeutic compounds is considered highly beneficial in cancer treatment, as suppression of this program can reduce cellular migration, invasiveness, stemness, and therapy resistance, thereby limiting metastatic potential and improving treatment responsiveness.^90,91^ Therefore, we investigated the functional impact of **[PtCl­(L** ^**1**^ **H)]­Cl** on cell migration and invasion. A wound healing assay in MKN45 cells showed a clear impairment of migration after **[PtCl­(L** ^**1**^ **H)]­Cl** treatment, with approximately 30% of wound closure compared with more than 50% in control cells (Figure 18d). To further evaluate these effects, we performed transmembrane assays with noncoated or Matrigel-coated cell culture inserts to study migration or invasion, respectively. **[PtCl­(L** ^**1**^ **H)]­Cl** markedly impaired both migratory and invasive capacities in MKN45 cells (Figure 18e), supporting its potential as an antitumor agent, warranting further preclinical studies.

**Figure 18:**
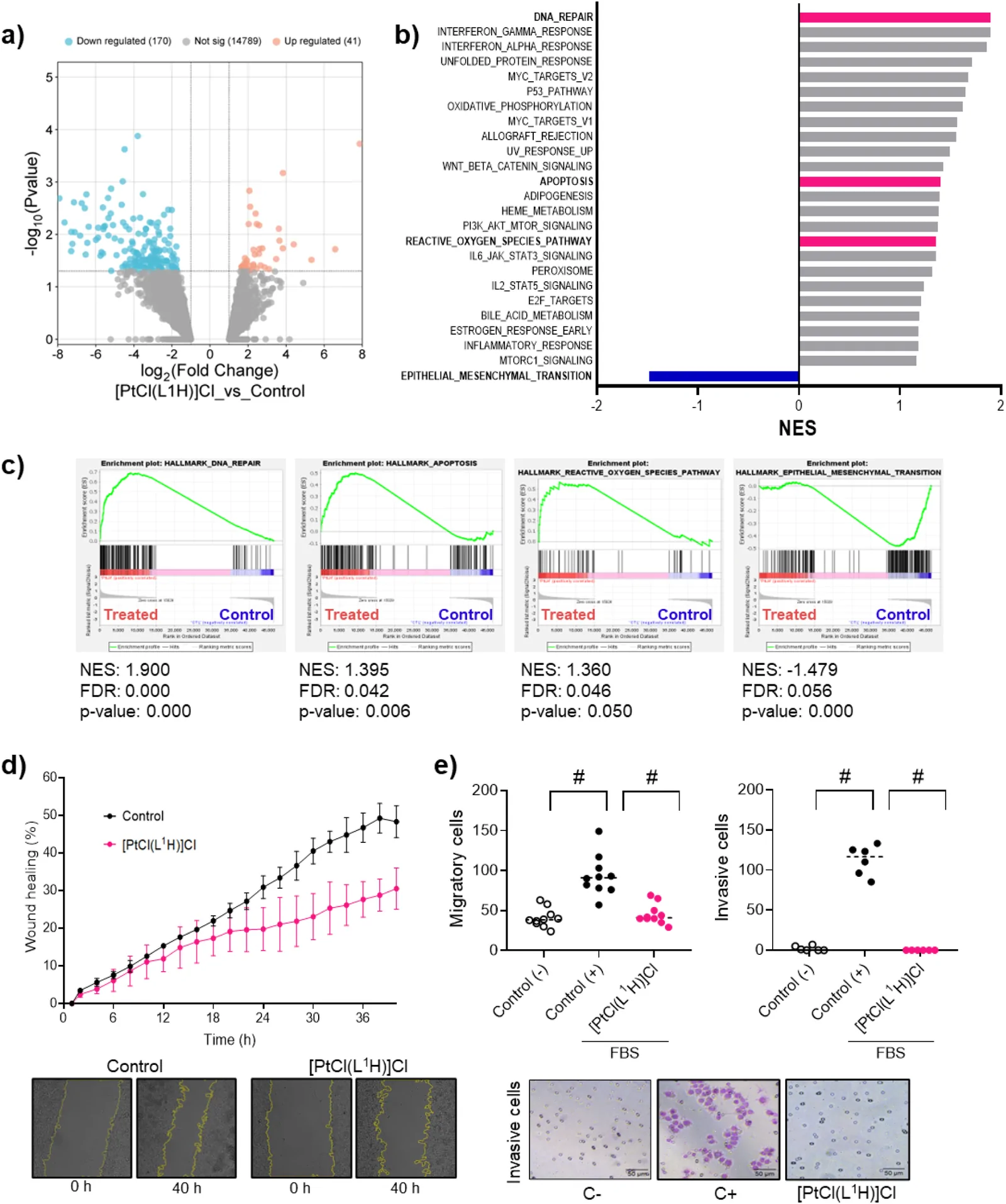
**[PtCl­(L^1^H)]­Cl** modulates transcriptional programs associated with DNA damage, apoptosis, oxidative stress, and EMT in gastrointestinal cancer cells. **a)** Volcano plots showing transcriptome-wide differential expression obtained from RNaseq comparing **[PtCl­(L^1^H)]­Cl** https://www.bioinformatics.com.cn/srplot. **b)** Gene sets enriched and depleted in the transcriptional profile of parental gastrointestinal cells treated with **[PtCl­(L^1^H)]­Cl** for 24 h compared to control cells. Shown are the NES (normalized enrichment score) values for each pathway using the Hallmark gene set and Signal-to-Noise metric, meeting the significance criteria: FDR < 25%. **c**) Enrichment plots for DNA repair, apoptosis, ROS, and EMT signaling pathways are depicted in **[PtCl­(L^1^H)]­Cl** -treated cells versus control cells. **d)** Wound healing assay in MKN45 cells treated with **[PtCl­(L^1^H)]­Cl** (10 μM). The graph shows the percentage of wound closure over the study time, analyzed using ImageJ (Mean ± SD is shown, *N* = 3). **e**) Transwell migration (left graph) or invasion (right graph) assay in MKN45 cells treated with **[PtCl­(L^1^H)]­Cl** (10 μM). Graphs show the quantification of stained migratory and invasive cells, respectively, using the transwell assay, without (Control-) and with 20% serum-conditioned medium (Control+ and **[PtCl­(L^1^H)]­Cl**-treated) at 48 h. Representative photographs of the stained invaded cells are shown. Scale bar: 50 μm. Statistical significance was evaluated by unpaired Student’s 2-tailed *t*-test (#*p* < 0.0001) compared to Control (+).

## Conclusions

This study demonstrates that chemical stabilization of a Schiff base scaffold through imine reduction represents an effective strategy for improving the biological performance of platinum-based anticancer agents. Compared with the nonreduced analogue **[PtCl­(L** ^**1**^)], the reduced complex **[PtCl­(L** ^**1**^ **H)]­Cl** displayed enhanced aqueous solubility and hydrolytic stability, leading to improved persistence under physiological conditions and more sustained biological activity. These findings identify solution stability as a key determinant influencing intracellular behavior, DNA interaction, and downstream cellular responses.

Importantly, **[PtCl­(L** ^**1**^ **H)]­Cl** retained potent activity against gastric cancer stem-like cells, effectively impairing self-renewal capacity and reducing stemness-associated features. Transcriptomic and functional analyses indicated that its anti-CSC activity is associated with the suppression of transcriptional programs related to stemness and epithelial–mesenchymal transition, accompanied by the inhibition of migratory and invasive phenotypes. Mechanistically, these effects were associated with the activation of DNA damage and oxidative stress responses, induction of apoptotic pathways, and reduced expression of pluripotency-related factors such as OCT3/4. Beyond identifying a new platinum­(II) scaffold, this work establishes ligand stabilization as a rational medicinal chemistry strategy to improve the efficacy of metal-based agents against aggressive and stem-like cancer cell populations.

## Experimental Section

### Materials and Methods

1,2-Phenylenediamine was acquired from TCI (Tokyo, Japan), 2-aminobenzaldehyde from BLDpharm (Shanghai, China), potassium tetrachloroplatinate­(II) from Alfa Aesar GmbH (Karlsruhe, Germany), and CDDP was supplied in solid by Alfa Aesar and in solution, donated by Ferrer FARMA. Oxaliplatin was purchased from Sigma-Aldrich (catalog no. O9512).

The solvents were acquired from VWR (Radnor, Pennsylvania). Elemental analysis was performed using a LECO CHNS-932 elemental analyzer, and thermogravimetric analysis (TGA) was conducted with a Thermobalance TGA Q500, TA Instruments, both located at the Interdepartmental Investigation Service (SIdI) at UAM. ATR-IR spectra were obtained on a PerkinElmer Spectrum 100 FT-IR spectrometer within a range of 4000–500 cm^–1^ UV–vis (ultraviolet–visible) spectra were obtained using a Thermo Evolution 220 spectrophotometer equipped with temperature control within ±0.1 °C. For **[PtCl­(L** ^**1**^ **H)]­Cl**, particle size and ζ-potential were measured in a 40 μM solution in water and Tris-HCl 5 mM on a Malvern Nano-ZS, Zetasizer Nano series.

The NMR (nuclear magnetic resonance) spectra were recorded at room temperature using a two-channel 300 MHz Bruker Avance IIIHD Nanobay spectrometer equipped with a 5 mm BBO ^1^H/X probe and Z gradients, and a 500 MHz Bruker AV spectrometer equipped with a 5 mm BBOF plus ^1^H–^19^F/X probe and Z gradients, located at the “Jesus Rodriguez” NMR User Laboratory maintained by SIdI. Chemical shifts were reported in ppm relative to the internal TMS (tetramethylsilane) reference. D_2_O, MeOH-*d*_4_, CHCl_3_-d and DMSO-*d*_6_ were acquired from VWR.

Mass spectrometry analysis was performed at SIdI using different ionization techniques. The masses of **L** ^**1**^ and **L** ^**1**^ **H** were determined on a Bruker Maxis II instrument employing APCI in positive mode with DIP (T = 200, range 50–2000 *m*/*z*). Ligand solutions were prepared by dissolving 1 mg in 1 mL of dichloromethane (DCM), followed by a 50:1000 dilution with DCM prior to measurement. The mass of **[PtCl­(L** ^**1**^)] was recorded on a Bruker Ultraflex III (MALDI-TOF/TOF) using positive ion detection over a range of 50–5000 Da. One milligram of the complex was dissolved in 1 mL of methanol and mixed with the matrix 2-[(2E)-3-(4-*tert*-butylphenyl)-2-methylprop-2-enylidene]­malononitrile (DCTB) in DCM prior to analysis. For **[PtCl­(L** ^**1**^ **H)]­Cl**, mass determination was performed on a Bruker TIMSTOF Pro instrument using ESI+ mode. The sample was prepared by dissolving 1 mg in 1 mL of methanol, followed by a 10:1000 dilution with methanol prior to measurement.

The purity of all new compounds used in biological studies was confirmed by elemental analysis, together with NMR spectroscopy. The experimental elemental analysis values were in good agreement with the calculated values, confirming that all compounds exhibited a purity greater than 95%.

In all the experiments, cells were treated with drugs following the appropriate sample preparation: **L** ^**1**^, **L** ^**1**^ **H**, and **[PtCl­(L** ^**1**^)] were dissolved in DMSO and then immediately diluted with culture medium to the appropriate concentration, with a final DMSO concentration of 1% (v/v). **[PtCl­(L** ^**1**^ **H)]­Cl** and oxaliplatin were dissolved in water.

### Synthesis and Characterization

#### Ligand *L* ^1^

A volume of 20 mL of an ethanol solution of 2-aminobenzaldehyde (2.37 mmol) was added dropwise, under stirring, to an an acid ethanol solution of 1,2-phenylenediamine (2.37 mmol) over 4 h at room temperature. Then, the reaction was allowed to proceed for an hour. After removal of the solvent, the residue was purified by extraction with a mixture of dichloromethane/NaOH (5% v/v). The organic phase was dried with anhydrous Na_2_SO_4_, filtered, and concentrated to dryness, giving a yellow solid.

**L** ^**1**^: Yield: 75%. ^1^H NMR (500 MHz, DMSO-*d*_6_): *δ* (ppm) 8.60 (s, 1H, H(7)), 7.43 (d, 1H, H(5), *J* = 7.79 Hz), 7.24 (s, 2H, -NH), 7.15 (t, 1H, H(3), *J* = 6.87 Hz), 6.99 (d, 1H, H(9), J = 6.26 Hz), 6.94 (t, 1H, H(11), *J* = 7.47 Hz), 6.78 (d, 1H, H(2), *J* = 7.24 Hz), 6.74 (d, 1H, H(12), *J* = 7.93 Hz), 6.60 (m, 2H, H(4) and H(10)) 4.94 (s, 2H, NH). ^13^C NMR (125.7 MHz, DMSO-*d*_6_) *δ*(ppm) 161.21 (C(7)), 149.34 (C(6)), 142.37 (C(13)), 137.17 (C(8)), 134.16 (C(5)), 131.42 (C(3)), 126.53 (C(11)), 117.53 (C(9)), 117.06 (C(1)), 116.63 (C(10)), 115.27 (C(2)), 114.76 (C(12)), 114.58 (C(4)). 2D [^1^H–^1^H] COSY NMR (500 MHz, DMSO-d^6^): *δ*(ppm) (7.43, 6.60), (7.43, 7.15), (7.15, 6.78), (7.15, 6.60), (6.99, 6.60), (6.94, 6.74), (6.94, 6.60). 2D [^1^H–^1^H] NOESY NMR (500 MHz, DMSO-d^6^): *δ*(ppm) (8.60, 7.43), (8.60, 6.99.) 2D [^1^H–^13^C] HSQC NMR (500 MHz, DMSO-d^6^): *δ*(ppm) (8.60, 161.21), (7.43, 134.16), (7.15, 131.42), (6.99, 117.51), (6.94, 126.53), (6.78, 115.27), (6.74, 114.76), (6.60, 114.56), (6.60, 116.63). 2D [^1^H–^13^C] HMBC NMR (500 MHz, DMSO-d^6^): *δ*(ppm) (8.60, 117.06), (8.60, 134.16), (8.60, 137.17), (8.60, 149.34), (7.43, 131.42), (7.43, 149.34), (7.43, 161.21), (7.43, 131.42), (7.15, 134.16), (7.15, 149.34), (6.99, 142.13), (6.99, 114.76), (6.94, 142.37), (6.94, 117.53), (6.78, 117.06), (6.78, 114.58), (6,74, 137.17), (6.74, 117.53), (6,60, 115.27), (6,60, 117.53), (6,60, 137.17). ATR-IR (cm^–1^): *υ*(N–H) 3442, 3367, 3278; *υ*(C–H) 3040, 2870; *υ*(C=N) 1610; *υ*(C–N) 1453, 1184.

#### Ligand L^1^H

Ligand **L** ^**1**^ **H** was synthesized by treating an ethanol solution of **L** ^**1**^ (0.41 mmol) with an excess of NaBH_4_ for 16 h. Afterward, 5 mL of H_2_O was added, and the mixture was stirred for an additional 30 min. The crude product obtained after removal of the solvent was purified by extraction with DCM/NaOH (5% v/v). The organic phase was collected, dried over anhydrous Na_2_SO_4_, filtered, and concentrated to dryness. The residue was purified by column chromatography using hexane:ethyl acetate (1:3) as an eluent, yielding a yellow solid.

**L** ^**1**^ **H:** Yield: 64%. Anal. Calc. for C_13_H_15_N_3_ (C_4_H_8_O_2_)_0.5_ (% C, N, H) 72.29, 18.73, 7.19. Found: 72.38, 18.82, 7.04. ^1^H NMR (500 MHz, MeOH-*d*_4_): *δ*(ppm) 7.18 (d, 1H, H(5), *J* = 7.32 Hz), 7.06 (t, 1H, H(4), *J* = 7.63 Hz), 6.78 (d, 1H, H(9), *J* = 7.78 Hz), 6.75–6.66 (m, 4H, H(2), H(3), H(10), H(12)), 6.60 (t, 1H, H(11), *J* = 7.02 Hz), 4.22 (s, 2H, H(7)). ^13^C NMR 125.7 MHz, MeOH-*d*_4_) *δ*(ppm) 147.21 (C(1)), 138.55 (C(6)), 135.95 (C(13)), 130.49 (C(5)), 129.25 (C(4)), 125.49 (C(8)), 121.10 (C(2)), 119.77 (C(1)­0), 119.51 (C(11)), 117.46 (C(9)), 117.34 (3), 113.38 (C(12)), 47.45 (C(7)). 2D [1H-1H] COSY NMR (500 MHz, MeOH-*d*_4_): (7.18, 6.71), (7.18, 7.06), (7.18,6.68), (7.18, 4.22), (6.78, 6.70), (6.70, 6.60). 2D [^1^H–^13^C] HSQC NMR (500 MHz, MeOH-*d*_4_): *δ*(ppm) (7.18, 130.49), (7.06, 129.25), (6.78, 117.34), (6.73, 117.46), (6.71, 113.07), (6.70, 119.77), (6.68, 121.10), (6.60, 119.51) (4.22, 47.45). 2D [^1^H–^13^C] HMBC NMR (500 MHz, MeOH-*d*_4_): *δ*(ppm) (7.18, 129.25), (7.18, 147.21), (7.06, 130.49), (7.06, 147.21), (6.78, 119.70), (6.78, 125.49), (6.73, 138.55), (6.73, 121.10), (6.70, 138.55), (6.70, 125.45), (6.70, 117.34), (6.60, 135.95), (6.60, 117.38), (6.60, 113.07) (4.24, 147.21), (4.24, 138.55), (4.24, 130.49), (4.24, 125.57). ATR-IR (cm^–1^): *υ*(N–H) 3405, 3331, 3241; *υ*(C–H) 3025, 2926; *υ*(C=C)­1617, 1587, 1499; *υ*(C–N) 1436, 1264. APCI+ (*m*/*z*) = [M + H]^+^: 214.

#### General Method for Ligands L^2^H to L^4^H Synthesis

In the first step, the corresponding diamine was selectively protected with di*tert*-butyl dicarbonate (Boc_2_O) as follows: 1,2-ethylenediamine, 1,3-propylenediamine, or 1,4-butylenediamine (1 mmol) was dissolved in 50 mL of dichloromethane and treated with a solution of 0.33 mmol of Boc_2_O in 70 mL of dichloromethane. The Boc_2_O solution was added dropwise at room temperature under stirring over 1 h. Then, the reaction mixture was stirred for 18 h, during which a white solid precipitated, corresponding to the deprotected diamine formed, and was removed by filtration. The dichloromethane filtrate was separated and washed three times with 25 mL of a 5% NaOH (w/v) aqueous solution. The organic phase was then dried over Na_2_SO_4_ and filtered, and the solvent was removed under reduced pressure to obtain the monoprotected diamine as an oil. The second step was to obtain the Schiff base ligands by condensation of the 2 mmol monoprotected diamine with 2 mmol of 2-aminobenzaldehyde. Both reagents were dissolved in 15 mL of ethanol, and the mixture was stirred for 2 h at room temperature. Then, the solvent was evaporated, and a cream-colored solid was precipitated with 20 mL of hexane and dried under vacuum.

The third step was the reduction of the imine group; 3 mmol of the product obtained (N-Boc-L,^2^ N-Boc-L^3^ or N-Boc-L^4^) was dissolved in 25 mL of ethanol and slowly treated with 9 mmol of NaBH_4_ divided in five portions and added at 30-min intervals. After the addition, 2 mL of water was introduced to quench the reaction, and the mixture was stirred for an additional 30 min. The solvent was removed under vacuum, and the residue was dissolved in 20 mL of dichloromethane and washed with 5% NaOH aqueous solution (6 × 25 mL). The organic phase was collected, dried over Na_2_SO_4_, and evaporated to afford the reduced ligand as an oil. Finally, the fourth step was the deprotection of the N-Boc group using trifluoroacetic acid (TFA). One mmol of the protected ligand (N-Boc-L^2^, N-Boc-L^3^ or N-Boc-L^4^) was dissolved in 3 mL of dichloromethane and treated with 9 mmol of TFA under ice-bath conditions. The solution gradually changed color from yellow to orange/red, and the reaction mixture was stirred for 4 h at room temperature. After thistime, 20 mL of NaOH 5% (w/v) was added dropwise, and the mixture was stirred for 30 min, resulting in a color change to pale yellow. The reaction mixture was extracted with dichloromethane (6 × 25 mL). The organic phase was dried over Na_2_SO_4_ and the solvent was removed to afford the final tridentate ligands.

**N-Boc-ethylenediamine:** Yield: 79%. ^1^H NMR (300 MHz, CHCl_3_-d): *δ*(ppm) 3.16 (q, 2H, −CH_2_, *J* = 6.12 Hz), 2.77 (t, 2H, −CH_2_, *J* = 6.17 Hz), 1.42 (s, 9H, −CH_3_). **N-Boc-propylenediamine:** Yield: 88% ^1^H NMR (300 MHz, CHCl_3_-d): *δ*(ppm) 3.19 (m, 4H, −CH_2_, *J* = 6.41 Hz), 2.76 (t, 2H, −CH_2_, *J* = 6.52 Hz), 1.60 (dd, 2H, −CH_2_, *J* = 6.89 Hz)­1.44 (s, 9H, −CH_3_). **N-Boc-butylenediamine:** Yield: 63% ^1^H NMR (300 MHz, CHCl_3_-d): *δ*(ppm) 3.19–3.15 (m, 2H, −CH_2_), 2.76 (t, 2H, −CH_2_, *J* = 6.88 Hz), 1.59–1.51 (m, 4H, −CH_2_), 1.43 (s, 9H, −CH_3_).

**N-Boc-L** ^**2**^: Yield: 82%. ^1^H NMR (300 MHz, CHCl_3_-d): *δ*(ppm) 8.34 (s, 1H, HC = N), 7.22–7.13 (m, 2H, Ph), 6.72–6.65 (m, 2H, Ph), 3.67 (t, 2H, −CH_2_, *J* = 5.86 Hz), 3.46 (dd, 2H, −CH_2_, *J* = 5.64 Hz), 1.45 (s, 9H, −CH_3_). **N-Boc-L** ^**3**^: Yield: 72%. *δ*(ppm) 8.33 (s, 1H, HC = N), 7.20–7.11 (m, 2H, Ph), 6.70–6.64 (m, 2H, Ph), 3.61­(t, 2H, −CH_2_, *J* = 6.63 Hz), 3.26 (dd, 2H, −CH_2_, *J* = 6.37 Hz), 1.87 (q, 2H, −CH_2_, *J* = 6.80 Hz), 1.44 (s, 9H, −CH_3_). **N-Boc-L** ^**4**^: Yield: 79%. *δ*(ppm) 8.32 (s, 1H, HC = N), 7.20–7.10 (m, 2H, Ph), 6.64–6.55 (m, 2H, Ph), 3.63–3.52 (m, 2H, −CH_2_), 3.46 (dd, 2H, −CH_2_, *J* = 7.21 Hz), 1.71 (q, 2H, −CH_2_, *J* = 7.21 Hz), 1.57 (q, 2H, −CH_2_, *J* = 7.42 Hz), 1.45 (s, 9H, −CH_3_).

**N-Boc-L** ^**2**^ **H:** Yield: 93% ^1^H NMR (300 MHz, CHCl_3_-d): *δ*(ppm) 7.11–6.97 (m, 2H, Ph), 6.74–6.62 (m, 2H, Ph), 3.80 (s, 2H, −CH_2_) 3.25 (d, 2H, −CH_2_, *J* = 5.64 Hz), 2.74 (t, 2H, −CH_2_, *J* = 6.03 Hz), 1.44 (s, 9H, −CH_3_). **N-Boc-L** ^**3**^ **H:** Yield: 95%^1^H NMR (300 MHz, CHCl_3_-d): *δ*(ppm) 7.18–6.98 (m, 2H, Ph), 6.71–6.61 (m, 2H, Ph), 3.77 (s, 2H, −CH_2_) 3.25–3.10 (m, 2H, −CH_2_), 2.71–2.59 (m, 2H, −CH_2_),1. 65 (q, 2H,-CH_2_, *J* = 6.03 Hz) 1.44 (s, 9H, −CH_3_). **N-Boc-L** ^**4**^ **H:** Yield: 85% ^1^H NMR (300 MHz, CHCl_3_-d): *δ*(ppm) 7.08 (t, 1H, Ph, *J* = 7.66 Hz), 7.01 (d, 1H, Ph, *J* = 7.52 Hz), 6.71–6.62 (m, 2H, Ph), 3.78 (s, 2H, −CH_2_) 2.66–2.56 (m, 2H, −CH_2_), 1.54–1.46 (m, 4H, −CH_2_), 1.44 (s, 9H, −CH_3_).

**L** ^**2**^ **H:** Yield: 91%. ^1^H NMR (300 MHz, CHCl_3_-d): *δ*(ppm) 7.17–7.01 (m, 2H, Ph), 6.74–6.64 (m, 2H, Ph), 3.81 (s, 1H, −CH_2_), 2.82 (t, 2H, −CH_2_, *J* = 5.84 Hz), 2.69 (t, 2H, −CH_2_, *J* = 5.80 Hz). ^13^C NMR 75 MHz, CHCl_3_-d): *δ*(ppm) 146.78 (C­(Ph)), 129.97 (C­(Ph)), 128.46 (C­(Ph)), 124.27 (C­(Ph)), 117.89 (C­(Ph)), (C­(Ph)), 115.83 (C­(Ph)), 52.97 (C­(−CH_2_)), 52.03 (C­(−CH_2_)), 41.86 (C­(−CH_2_)). ATR-IR (cm^–1^): *υ*(N–H) 3426, 3353, 3241; *υ*(C–H) 2918, 2845; *υ*(C=C)­1623, 1585, 1492; *υ*(C–N) 1451, 1260. **L** ^**3**^ **H:** Yield: 71%. ^1^H NMR (300 MHz, CHCl_3_-d): *δ*(ppm) 7.11–7.01 (m, 2H, Ph,), 6.71–6.64 (m, 2H, Ph), 3.80 (s, 1H, −CH_2_), 2.77 (t, 2H, −CH_2_, *J* = 6.79 Hz), 2.69 (t, 2H, −CH_2_, *J* = 6.79 Hz), 1.64 (q, 2H, −CH_2_, *J* = 6.86 Hz). ^13^C NMR 75 MHz, CHCl_3_-d) *δ*(ppm) 146.88 (C­(Ph)), 129.89 (C­(Ph)), 128.38 (C­(Ph)), 124.30 (C­(Ph)), 117.80 (C­(Ph)), 115.78 (C­(Ph)), 53.28 (C­(−CH_2_)), 47.14 (C­(−CH_2_)), 40.41 (C­(−CH_2_)), 33.87 (C­(−CH_2_)). ATR-IR (cm^–1^): *υ*(N–H) 3400, 3362, 3288; *υ*(C–H) 2931, 2849; *υ*(C=C)­1613, 1585, 1499; *υ*(C–N) 1458, 1281. **L** ^**4**^ **H:** Yield: 95%. ^1^H NMR (300 MHz, CHCl_3_-d): *δ*(ppm) 7.11–7.00 (m, 2H, Ph,), 6.72–6.64 (m, 2H, Ph), 3.79 (s, 2H, −CH_2_), 2.69 (t, 2H, −CH_2_, *J* = 6.80 Hz), 2.64 (t, 2H, −CH_2_, *J* = 6.61 Hz), 1.62–1.42 (m, 4H, −CH_2_). ^13^C NMR 75 MHz, MeOH-*d*_4_) *δ*(ppm) 147.32 (C­(Ph)), 130.80 (C­(Ph)), 129.16 (C­(Ph)), 125.80 (C­(Ph)), 119.36 (C­(Ph)), 117.45 (C­(Ph)), 52.12 (C­(−CH_2_)), 49.94 (C­(−CH_2_)), 42.43 (C­(−CH_2_)), 31.66 (C­(−CH_2_)), 28.07 (C­(−CH_2_)). ATR-IR (cm^–1^): *υ*(N–H) 3353, 3293, 3202; *υ*(C–H) 2926, 2853; *υ*(C=C)­1615, 1583, 1494; *υ*(C–N) 1458, 1282.

#### Complex [PtCl­(L^1^H)]­Cl

A mixture of *cis*-[PtCl_2_(DMSO)_2_] (0.071 mmol) and **L** ^**1**^ **H** (0.071 mmol) in 3 mL of chloroform was stirred at 60 °C for 1 h. Afterward, a pink solid and a red solution were obtained. The solid was isolated by centrifugation and washed three times with 2 mL of dichloromethane.

**[PtCl­(L** ^**1**^ **H)**]**Cl**: Yield: 84%. Anal. Calc. for C_13_H_15_N_3_Cl_2_Pt (% C, N, H) 32.58, 8.77, 3.15. Found: 32.35, 8.58, 3.44. ^1^H NMR (500 MHz, D_2_O): *δ*(ppm) 7.80 (d, 1H, H(5), *J* = 8.85 Hz), 7.55–7.43 (m, 6H, H(2), H(3), H(4), H(9), H(11), H(12)), 7.40 (t, 1H, H(10), *J* = 7.48 Hz), 4.94 (s, 2H, H(7)). ^13^C NMR (125.7 MHz, D_2_O): *δ*(ppm) 146.70 (C(1)), 144.00 (C(6)), 138.76 (C(13)), 134.62 (C(9)), 133.67 (C(12)), 132.83 (C(2)), 132.50 (C(8)), 132.11 (C(4)), 130.64 (C(10)), 129.28 (C(3)), 126.92 (C(11)), 126.15 (C(5)), 57.77 (C(7)). 2D [^1^H–^1^H] COSY NMR (500 MHz, D_2_O): (7.80, 7.47), (7.54, 7.40). 2D [^1^H–^1^H] NOESY NMR (500 MHz, D_2_O): (7.80, 4.94), (7.54, 4.94). 2D [^1^H–^13^C] HSQC NMR (500 MHz, D_2_O): *δ*(ppm) (7.80, 123.87), (7.53, 132.34), (7.52, 131.39), (7.44, 124.63), (7.44, 130.55), (7.44, 129.83), (7.44, 124.63), (7.39, 128.26), (4.94, 55.49). 2D [^1^H–^13^C] HMBC NMR (500 MHz, D_2_O): *δ*(ppm) (7.80, 130.69), (7.80, 141.44), (7.80, 144.44), (7.54, 55.49), (7.54, 131.55), (7.54, 136.28), (7.47, 126.83), (7.45, 129.83), (7.45, 144.44), (7.44, 123.82), (7.44, 128.11), (7.44, 141.44), (7.40, 124.68), (7.40, 129.83), (4.94, 130.26), (4.94, 132.41), (4.94, 136.28).^195^Pt NMR (64.5 MHz, D_2_O): −2618 ppm. ATR-IR (cm^–1^) *υ*(C–H) 3060, 2855, 2769; *υ*(C–N) 1560, 1499, 1233. ESI^+^-MS (*m*/*z*) = [M]^+^: 443.

#### Complex [*PtCl­(L* ^1^)]

**[PtCl­(L** ^**1**^ **H)]­Cl** (1.0 mmol) was dissolved in methanol (20 mL) and stirred at room temperature under a gentle stream of air for 16 h, while maintaining the solvent volume constant. After 1 h, a gradual precipitation of a purple solid was observed, which increased over time. After 16 h, the solvent was fully evaporated under the stream of air, and the solid was washed with water (1 mL) and diethyl ether (3 × 1 mL) and dried under vacuum. **[PtCl­(L** ^**1**^)] crystals were obtained by solubilizing 25 mg of **[PtCl­(L** ^**1**^ **H)]­Cl** in 3 mL of methanol and leaving it to evaporate slowly in open air until dark purple crystals formed. The crystalline solid was collected and washed with water, diethyl ether, and hexane.

**[PtCl­(L** ^**1**^)]: Yield: 89%. Anal. Calc. for C_13_H_12_N_3_ClPt·(C_6_H_14_)_0.20_ (% C, N, H) 37.15, 9.15, 3.47. Found: 37.07, 9.41, 3.34. ^1^H NMR (500 MHz, DMSO-*d*_6_): *δ* (ppm) 9.34 (s, 1H, H(7)), 8.18 (d, 1H, *J* = 7.32 Hz, H(9)), 7.78 (s, 2H, NH), 7.64 (d, 1H, *J* = 8.4 Hz, H(5)), 7.53 (s, 2H, NH), 7.32–7.29 (m, 2H, H(10), H(12)), 7.23 (t, 1H, *J* = 8.85 Hz, H(3)), 7.14 (t, 1H, *J* = 7.48 Hz, H(11)), 7.04 (d, 1H, *J* = 9.01 Hz, H(2)), 6.30 (t, 1H, *J* = 7.63 Hz, H(4)). ^13^C NMR (125.7 MHz, DMSO-*d*_6_) *δ*(ppm):149.95 (C(13)),147.04 (C(6)), 140.84 (C(7)), 138.33 (C(8)), 133.43 (C(5)), 130.80 (C(3)), 127.33 (C(12)), 127.13 (C(10)), 125.38 (C(11)), 121.14 (C(2)), 117.43 (C(1)), 115.35 (C(9)), 113.65 (C(4)). 2D [^1^H–^1^H] COSY NMR (500 MHz, DMSO-*d*_6_: (8.18, 7.31), (7.64, 6.30), (7.31, 7.14), (7.23, 7.02), (7.23, 6.30). 2D [^1^H–^1^H] NOESY NMR (500 MHz, DMSO-*d*_6_) *δ*(ppm) (9.34, 8.18), (9.34, 7.64) 2D [^1^H–^13^C] HSQC NMR (500 MHz, DMSO-*d*_6_): *δ*(pm) (9.34, 140.84), (8.18, 115.21), (7.64, 133.52), (7.32, 127.64), (7.31, 127.31), (7.23, 130.84), (7.14, 125.40), (7.04, 121.14), (6.30, 113.64). 2D [^1^H–^13^C] HMBC NMR (500 MHz, DMSO-*d*_6_): *δ*(pm) 7.80, 130.69), (7.80, 141.44), (7.80, 144.44), (7.54, 55.49), (7.54, 131.55), (7.54, 136.28), (7.47, 126.83), (7.45, 129.83), (7.45, 144.44), (7.44, 123.82), (7.44, 128.11), (7.44, 141.44), (7.40, 124.68), (7.40, 129.83), (4.94, 130.26), (4.94, 132.41), (4.94, 136.28) ^195^Pt NMR (64.5 MHz, DMSO-*d*_6_): −2037 ppm. ATR-IR (cm^–1^) *υ*(N–H) 3323; *υ*(C–H) 3012; *υ*(C=N) 1558; *υ*(C = C) 1496; *υ*(C–N) 1447, 1374, 1217. MALDI-MS (*m*/*z*) = [M + H]^+^: 442.

### Stability Studies

#### UV–Vis Monitoring

The stability of **L** ^**1**^ and **L** ^**1**^ **H** was studied in an aqueous solution containing 1% (v/v) DMSO. For the platinum complexes, the studies were conducted in four different media: water, 0.9%(w/v) NaCl, 5 mM Tris-HCl, 5 mM NaClO_4_, and 20 mM phosphate buffer (pH 7.4). A 10 mM stock solution in DMSO was prepared for compounds **L** ^**1**^, **L** ^**1**^ **H**, and **[PtCl­(L** ^**1**^)]. From this stock solution, different aliquots were taken to prepare six samples in the corresponding media with final concentrations of 5, 10, 20, 40, 60, and 80 μM, containing 1% (v/v) of DMSO. For **[PtCl­(L** ^**1**^ **H)]­Cl**, a 160 μM stock solution was prepared in water. From this, dilutions were made to obtain six samples with final concentrations of 5, 10, 20, 40, 60, and 80 μM in the different media. The samples were monitored at 37 °C for 24 h. A UV–vis spectrum was recorded every 20 min during the first 5 h, and then every h for the next 20 h.

#### ^1^H NMR Monitoring

The stability of complexes was monitored at 25 °C over 24 h in a two-channel 300 MHz Bruker Avance IIIHD Nanobay spectrometer equipped with a 5 mm BBO^1^H/X probe and Z gradients, and a 500 MHz Bruker AV spectrometer equipped with a 5 mm BBOFplus ^1^H–^19^F/X probe and Z gradients. An ^1^H NMR spectrum was recorded every 60 min for 24 h. The complex **[PtCl­(L** ^**1**^)] was dissolved in DMSO-*d*_6_; moreover, two drops of D_2_O were added to the sample for additional analysis. **[PtCl­(L** ^**1**^ **H)]­Cl** was dissolved in D_2_O and a TSP-*d*_4_ solution (3-(trimethylsilyl)-2,2,3,3-tetradeuteropropionic acid) was used as a reference.

#### Conductivity Monitoring

Conductivity was monitored over 24 h at 37 °C using a Crison EC-Meter Basic 30+. One millimolar solutions of complexes **[PtCl­(L** ^**1**^ **H)]­Cl** and **[PtCl­(L** ^**1**^)] were prepared in DMF, DMSO, water, and a mixture of water with 1% (v/v) DMSO.

### Cell Culture

The human gastric cancer cell lines AGS (primary tumor; ATCC/LGC Standards, Spain), MKN28, and MKN45 (poorly differentiated; DSMZ: Deutsche Sammlung von Mikroorganismen und Zellkulturen GmbH), pancreatic cell line PANC1 (ATCC), and nontumor cell line GES-1 (human gastric mucosal epithelial cells, Cytion, product number 305428) were cultured in RPMI 1640 (Sigma), with the exception of AGS cells, which were culwhtured in HAM’SF12, according to the manufacturer’s datasheet. All the culture media were supplemented with 10% FBS (v/v), 2 mM l-Glutamine, Fungizone (2.5 μg/mL), and Gentamicin (0.035 mg/mL). Cultures were kept at 37 °C, 5% CO_2_, and 95% humidity. Mycoplasma contamination tests are frequently run in our laboratory. Experiments were performed between passages two and eight.

To generate CSC-enriched 3D spheres (tumorospheres), MKN45 and PANC1 cells were cultured in DMEM/F12 (Invitrogen) supplemented with B-27 (10889038, Gibco), 2 mM l-glutamine, Fungizone (2.5 μg/mL), gentamicin (0.035 mg/mL), and 20 ng/mL bFGF (PeproTech). These cells were seeded in ultralow-attachment (ULA, Corning) 6-well plates (3 × 10^5^ cells/3 mL in each well) and kept in a humidified incubator (5% CO_2_ at 37 °C). Fresh medium was added to the culture every few days, and the culture was analyzed daily for tumorosphere formation (up to the seventh day). For serial passaging, tumorospheres were harvested using a 40-μm cell strainer (Corning, 353180), dissociated into single cells with trypsin, and reseeded under identical conditions. Secondary spheres were typically used for all downstream experiments.^92^

### Cell Viability

Cell viability in parental cells was assessed using a crystal violet-based staining method. 5 × 10^3^ cells/well were seeded in 96-multiwell plates. The following day, cells were treated with compounds at different concentrations. To evaluate the role of ROS in cytotoxicity, the growth medium was supplemented with NAC 0.5 mM, a general ROS scavenger.^93^ After 48 h, cells were fixed with 0.1% glutaraldehyde (20 min) and then washed twice with PBS 1x. Then, cells were stained with 0.1% crystal violet (30 min) and a colorimetric assay set at 595 nm was used to estimate the number of cells per well.

To evaluate cell viability in CSCs, an MTS staining method was used in both parental cells and tumorospheres. Adherent cells (5 × 10^3^ per well) were seeded in 96 multiwell plates, and the following day, they were treated with compounds at different concentrations. CSCs (MKN45 and PANC1) enriched sphere-derived cultures were seeded in ULA 96-well plates at a concentration of 5 × 10^3^ cells per well to form second-generation spheres. Four days later, secondary spheres were treated. After 72 h (depending on the experiment), 10 μL/well MTS solution was added to each well, and after incubation in the darkness for 1–4 h (37 °C, 5% CO_2_), absorbance was recorded at 490 nm.

In all of the experiments, IC_50_ values were calculated by using the GraphPad Prism program: nonlinear regression was employed to fit the data to the log (inhibitor) versus response (variable slope).

### Cellular Uptake and Distribution by ICP-MS

To assess the uptake and intracellular distribution of the compounds, MKN45 cells were seeded in p60 plates (1.5 × 10^6^ cells per plate) and allowed to attach overnight (37 °C, 5% CO_2_). Cells were incubated with cisplatin, **[PtCl­(L** ^**1**^)] and **[PtCl­(L** ^**1**^ **H)]­Cl** for 3 h (20 μM). Then, the cells were washed with PBS and trypsinized. In suspension, the cells were counted, 10^6^ cells were separated, washed twice with cold PBS, and centrifuged (1000 rpm, 5 min) to obtain a pellet. To disrupt the cellular membrane, the pellet was resuspended in lysis buffer (10 mM Tris, 1.5 mM MgCl_2_, 140 mM NaCl, pH 8.0–8.3) with Nonidet P40 (0.02%)^53^ After 15 min of incubation on ice, the suspension was centrifuged at 1300 g for 2 min at 4 °C, and the nuclear fraction (pellet) was separated from the cytoplasmatic fraction (supernatant). The Pt content in the two fractions was measured, after digestion in an open vase with HNO_3_ (65%), H_2_O_2_, and HCl, evaporated, and resuspended in ultrapure water to obtain a 2.0% (v/v) nitric acid solution, by ICP-MS on an Agilent 8900 ICP-MS Triple Quad, with ^187^Re and ^193^Ir used as internal standards. The protocol was performed at the ICP-MS unit of SIdI-UAM, which fulfills the Quality Management System IQNet – ISO 9001:2008.

### Fluorescence Binding Studies with Bovine Serum Albumin (BSA)

Bovine serum albumin (BSA, MW = 66.5 kDa) and all other reagents were of analytical grade and were used as received. All experiments were carried out in 5 mM Tris-HCl buffer (pH 7.4). For studies with **[PtCl­(L** ^**1**^)], samples contained 1% DMSO (v/v) in accordance with its previously established stability conditions. For **[PtCl­(L** ^**1**^ **H)]­Cl**, no DMSO was used.

A stock solution of BSA was prepared by dissolving approximately 13.3 mg of protein in 2.0 mL of 5 mM Tris-HCl buffer (pH 7.4). The solution was centrifuged at 10,000 rpm for 5 min to remove air bubbles. The exact concentration of BSA was determined spectrophotometrically at 278 nm using a molar extinction coefficient of 44,000 M^–1^ cm^–1^, affording stock concentration on the order of 10^–4^ M. For fluorescence titrations, a series of 8 samples was prepared at a constant BSA concentration of 1.0 × 10^–6^ M and increasing complex concentrations in the 0.1–10 × 10^–6^ M range. Sample solutions were prepared in a 5 mM Tris-HCl buffer under the solvent conditions described above for each complex. BSA was added last in all cases to minimize possible protein denaturation, particularly in DMSO-containing media. The samples were incubated at 37 °C for 24 h prior to measurement.

Fluorescence emission spectra were recorded on a Cary Eclipse spectrofluorometer using an excitation wavelength of 280 nm, corresponding to the intrinsic fluorescence of BSA tryptophan residues. Emission spectra were collected over the appropriate wavelength range (typically 300–500 nm). A buffer blank was recorded and used for baseline correction. Samples were measured sequentially in order of increasing complex concentration. UV–vis absorption spectra of all samples were recorded immediately after fluorescence measurements in order to correct the fluorescence data for inner-filter effects according to eq 1,^67^

1 $$F_{corr}=F_{obs}\times 10^{\left(A_{ex}+A_{em}\right)/2}$$

where F_obs_ is the observed fluorescence intensity, and A_ex_ and A_em_ are the absorbances at excitation (280 nm) and emission wavelengths, respectively.

The corrected fluorescence intensities were used to calculate quenching constants via the Stern–Volmer eq 2:

2 $$\frac{F_{0}}{F}=1+K_{SV}\left[Q\right]$$

and binding parameters using eq 3:

3 $$\log\left(\frac{F_{0}-F}{F}\right)=\log K_{BIN}+n\log\left[Q\right]$$

where F_0_ and F are the fluorescence intensities in the absence and presence of the quencher, respectively. [Q] is the concentration of the complex, K_SV_ is the Stern–Volmer constant, K_BIN_ is the binding constant, and *n* is the number of binding sites. Linear regression analysis of the corresponding plots provided the values of K_SV_ and K_BIN_ and *n.*

### Electrophoresis Assays

The commercial plasmid pBR322 (0.5 μg/μL) (GENECUST) was diluted in Tris-HCl (5 mM, pH = 7.4) to a final concentration of 0.0625 μg/μL. A volume of 19 μL of this stock solution was taken, and 1 μL of the complex solution was added. The concentrations of the complexes varied to achieve molar ratios of r_i_ = 0.05, 0.10, 0.15, and 0.20 (where r_i_ = [complex]/[DNA]).

Stock solutions of complexes **[PtCl­(L** ^**1**^)] and **[PtCl­(L** ^**1**^ **H)]­Cl** were prepared at 5 mM and 1 mM in DMSO and water, respectively. Cisplatin was prepared in a 10 mM NaClO_4_ solution. Aliquots from the stock solutions were diluted in water to prepare 50 μL of working solutions at the required concentrations, allowing the addition of 1 μL to the plasmid solution to achieve the desired r_i_ ratios. A 1 kb ladder (VWR N551) was used as a reference. As a control, 19 μL of pBR322 (0.0625 μg/μL) was incubated with 1 μL of water.

All samples were incubated for 24 h at 37 °C and shaken at 300 rpm in an incubator shaker (Eppendorf ThermoMixer C). Subsequently, 2 μL of the loading buffer (0.25% (w/v) bromophenol blue and 30% (v/v) glycerol was added to 10 μL of each sample. Sample mobility was analyzed by 1.2% (w/v) agarose gel electrophoresis in 1× TAE buffer (Tris, acetate, and EDTA) at 70 V for 90 min. Agarose gels were stained with ethidium bromide (5 μg mL^–1^), and the bands were visualized using a UV transilluminator (UVITEC Cambridge UVIDOC HD2 instrument).

### Viscosity Assays

Viscosity measurements were performed using an automated AND viscometer (model SV-1A) maintained at 25.0 ± 0.1 °C with a thermostated water bath equipped with a water-jacket accessory. All experiments were carried out at a fixed CT-DNA concentration of 4.62 × 10^–5^ M in Tris-HCl 5 mM, while varying the [compound]/[CT-DNA] molar ratio (1/R ranging from 0.0 to 0.4). Stock solutions of the test compounds were prepared at 5 mM: **[PtCl­(L** ^**1**^)] was dissolved in DMSO, **[PtCl­(L** ^**1**^ **H)]­Cl** in water, cisplatin in a 10 mM NaClO_4_ aqueous solution, and ethidium bromide (Sigma) in water. Appropriate aliquots of each stock solution were diluted with 5 mM Tris–HCl buffer containing CT-DNA to achieve the desired molar ratios. For **[PtCl­(L** ^**1**^)], the final DMSO concentration in the assay mixture was 3% (v/v). The effect of compound binding on DNA viscosity was evaluated by plotting the relative viscosity of CT-DNA, expressed as (η/η_0_)^1/3^, against 1/R, where η_0_ and η correspond to the viscosities of CT-DNA in the absence and presence of the compound, and 1/R = [compound]/[CT-DNA].

### Clonogenic Assay

For the clonogenic assay, we used two different approaches. (i) MKN45 cells were treated with the metallodrugs for 24 h: **[PtCl­(L** ^**1**^)] 25 μM, **[PtCl­(L** ^**1**^ **H)]­Cl** 10 μM. Then, the cells were cultured in a 6-well plate at a very low concentration (2 × 10^3^ per well) and incubated for 10 days. (ii) MKN45 cells were cultured in a 6-well plate at a very low concentration (2 × 10^3^ per well), incubated overnight to ensure attachment, and then treated with the drugs. After 10 days, the cells were fixed with 1% glutaraldehyde and stained with 0.1% crystal violet. The number of colonies was quantified directly and reflected as CFUs: Colony Forming Units.

### Tumor Sphere Assay

Gastric CSC spheres were generated by culturing pretreated MKN45 cells (1,000–5,000 cells/mL) in ULA 24-well plates (Corning). Seven days later, spheres were counted with an inverted EVOS FL microscope (Thermo Fisher Scientific) using a 10X objective with phase contrast. Sphere counts are represented as the number (N°) of spheres/mL.

### Cell Cycle Analysis

MKN45 cells were treated for 24 h with **L** ^**1**^ and **[PtCl­(L** ^**1**^)] (25 μM), and with **L** ^**1**^ **H** and **[PtCl­(L** ^**1**^ **H)]­Cl** (5 μM). Cisplatin was included as a reference drug (10 μM). Then, cells were trypsinized, fixed with 70% cold EtOH, and stored at 4 °C overnight. Cells were incubated with PI (50 μg/mL) and RNase (10 μg/mL) for 30 min, and DNA content was evaluated using a CytoFLEX Flow Cytometer (Becton Dickinson, SIdI UAM). Results were analyzed using CytExpert 2.4 and Kaluza Analysis 2.3 software.

### ROS and Mitochondrial Assays

We used flow cytometry to study the mitochondrial mass, mitochondrial membrane potential (ΔΨm), and ROS generation in MKN45 cells. MKN45 cells (5 × 10^5^) were seeded in 6-well dishes and cultured overnight. Then, the cells were treated for 24 h with the complexes. Cells were trypsinized, stained with Mitoprobes, and resuspended in buffer, and 2 μg/mL DAPI (Sigma) was used to exclude dead cells. A CytoFLEX Flow Cytometer (Becton Dickinson, SIdI UAM) was used. For ROS production measurement, MitoSOX (M36008, Invitrogen) was used at 10 μM for 30 min at 37 °C. For mitochondrial mass and ΔΨm measurement, Mitotracker Deep Red (MTDR, M22426, Invitrogen) and Mitotracker Red CMXRos (CMXROS, M7512, Invitrogen) were used, respectively. Probes were incubated with cells for 30 min at 37 °C, 5% CO_2_, at a concentration of 1 nM (MTDR) and 20 nM (CMXROS). To determine mitochondria functionality, the ratio between ΔΨm (MitoTracker Red CMXRos) versus Mit. Mass (MitoTracker Deep Red) was calculated. Data were analyzed with CytExpert 2.4 software.

### Western Blotting

Total protein extracts were obtained by adding the lysis buffer: 25 mM HEPES pH 7.5, 0.3 M NaCl, 1.5 mM MgCl_2_, 0.2 mM EDTA, 0.5 mM DTT, 20 mM β-glicerophosphate, 0.1 mM Na_3_VO_4_, 0.1% Triton X-100; in the presence of protease inhibitors (Protease and Phosphatase Inhibitor Cocktail, Sigma-Aldrich, PPC1010). Twenty microgram of protein per sample were loaded and resolved using 8%, 10%, or 15% SDS-PAGE polyacrylamide gels, and then transferred onto PVDF membranes (IPVH00010, Merck Millipore), followed by immunodetection using appropriate antibodies purchased from Cell Signaling Technology: p-p38^Thr180/Tyr182^ (#4511), p-p53^Ser15^ (#9284, 1:2000), p-CHK1^ser296^ (#2349), BAX (#9942), Notch1 cleaved (#3608), and GAPDH (#2118, 1:5000); purchased from Selleckchem: p-STAT3^Tyr705^ (#F0157, 1:2000); purchased from Santa Cruz Biotechnology: OCT3/4 (#sc-5279), Mcl-1 (#sc-819), PARP-1 (#sc-7150), and β-Actin (#sc-47778); and purchased from Sigma-Aldrich: α-tubulin (T9026, 1:10000). Unless indicated all antibodies were diluted to 1:1000. Secondary antibodies conjugated with horseradish peroxidase were purchased from BioRad, and chemiluminescence detection was performed using ECL (Cytiva, RPN2209).

### RNA Sequencing and Bioinformatic Analysis

Gastrointestinal cancer cells (MKN-28, MKN-45, and PANC1) were challenged with **[PtCl­(L** ^**1**^ **H)]­Cl** (5 μM) for 24 h. Total RNA samples were processed by Macrogen Inc. (Seoul, Republic of Korea) for transcriptome sequencing. Libraries were prepared using the TruSeq Stranded mRNA Library Prep Kit (Illumina), and paired-end sequencing (2 × 151 bp) was performed on an Illumina platform. The human reference genome GRCh38 and NCBI_109 annotation release were used for downstream analyses. Raw sequencing reads were quality-checked using FastQC (v0.11.7) and processed with Trimmomatic (v0.38) to remove adapters and low-quality bases. Reads shorter than 36 bp after trimming were discarded. Trimmed reads were aligned to the human reference genome (GRCh38) using HISAT2 (v2.1.0), and transcript assembly and quantification were performed using StringTie (v2.1.3b). Expression values were calculated as read counts, FPKM, and TPM. Differential gene expression analysis was performed using DESeq2 based on normalized read counts. RNA-sequencing data were analyzed using GSEA software (v. 4.3.2) and the Hallmark gene set database. Statistical significance was determined using 1000 permutations with a false discovery rate (FDR) < 25%.

### Cell Migration and Invasion Assay

Migration and invasion assays were performed with cell culture inserts with a transparent PET membrane (6.5 mm diameter, 8.0 μm pore size: Transwell migration and invasion assays, Falcon, Corning Inc., Corning, NY, USA) or coated with 6 μg growth factor-reduced Matrigel (BD Biosciences, Bedford, MA, USA), respectively. MKN-45 cells (1.5 × 10^5^) were seeded in the upper chamber. FBS (20%, v/v) was used as a chemoattractant and placed in the bottom chamber. Cells were treated with **[PtCl­(L1H)]­Cl** (5 μM) and allowed to migrate or invade for 48 h at 37 °C with 5% CO_2_. Then, nonmigrating and noninvading cells were removed using a cotton swab, and the filters were stained with Diff-Quik (Dade Behring, Newark, DE, USA). Migratory and invasive cells were counted in 50 fields of maximum migration or invasion with a light microscope at 40× magnification. Representative images were captured using an Axiophot Zeiss microscope (SEMOC, IIBM-CSIC-UAM). Cell migration was also studied using a wound healing method. The wound healing (Scratch) assay was used to study the effects of **[PtCl­(L** ^**1**^ **H)]­Cl** on MKN45 cell migration. The cells were cultured to 80% confluence in 24-well plates (7 × 10^5^ cells/0.5 mL). Subsequently, the cell monolayer was scratched with a sterile 10 μL pipet tip. The medium was removed, and 500 μL of fresh media with **[PtCl­(L** ^**1**^ **H)]­Cl** (10 μM) was added. The migratory ability of the treated cells was monitored from 0 to 48 h with a Cell Observer microscope (Observer Z1, Zeiss, IIBM-CSIC-UAM) and compared to nontreated cells.

### Statistics and Reproducibility

Statistical analyses were performed using GraphPad Prism 8.0 Student’s 2-tailed *t*-test or one-way ANOVA with Dunnett’s posttest. Values of *p < 0.05 were considered significant. Volcano plots were generated using the SRPlot online platform: https://www.bioinformatics.com.cn/srplot.^94^

## Supplementary Material





## Data Availability

All data supporting the findings of this study are available within the paper and its Supporting Information. All uncropped blots are collected in Figure S56 Raw sequencing data and gene counts have been deposited in the NCBI Sequence Read Archive (SRA) and Gene Expression Omnibus (GEO) under accession number GSE331422.

## Notes

Molecular Formula Strings (CSV)
Contents: Experimental details for single-crystal X-ray diffraction; crystallographic data and refinement parameters; ^1^H, ^13^C, and ^195^Pt NMR spectra; 2D NMR spectra (HSQC, HMBC, COSY, and NOESY); ESI-MS and MALDI-MS spectra; thermogravimetric analysis (TGA); UV–Vis stability studies; supplementary biological data, BSA fluorescence quenching, uncropped Western blot images, supplementary Figures S1–S56, and Tables S1–S2 (PDF)

## Abbreviations

AGS: gastric adenocarcinoma cell line
ATR: ataxia telangiectasia and Rad3-related
BAX: Bcl-2-associated protein X
Boc_2_O: di*tert*-butyl dicarbonate
BSA: bovine serum albumin
CC: covalently closed circular
CCDC: Cambridge Crystallographic Data Center
CDDP: cis-diamminedichloroplatinum­(II)
CFU: colony-forming units
CHK1: checkpoint kinase 1
CMX-ROS: chloromethyl-X-rosamine
COSY: correlation spectroscopy
CSC: cancer stem cell
CT: calf thymus
DAPI: 4′,6-diamidino-2-phenylindole
DDR: DNA damage response
DLS: dynamic light scattering
DMEM: Dulbecco’s Modified Eagle Medium
DMSO: dimethyl sulfoxide
DNA: DNA
DTT: dithiothreitol
EDTA: ethylenediaminetetraacetic acid
EMT: epithelial-to-mesenchymal transition
EPR: electron paramagnetic resonance
ESI-MS: electrospray ionization mass spectrometry
EtBr: ethidium Bromide
FBS: fetal bovine serum
FDR: false discovery rate
FLOT: 5-fluorouracil, leucovorin, oxaliplatin, and docetaxel, FOLFOX-based, 5-fluorouracil, leucovorin, and oxaliplatin
GAPDH: glyceraldehyde-3-phosphate dehydrogenase
GC: gastric cancer
GSEA: gene set enrichment analysis
HAM’SF12: Ham’s F12 Nutrient Mixture
HEPES: 4-(2-hydroxyethyl)-1-piperazineethanesulfonic acid
HMBC: heteronuclear multiple bond correlation
HSQC: heteronuclear single quantum coherence
ICP-MS: inductively coupled plasma mass spectrometry
IC_50_: half-maximal inhibitory concentration
IR: infrared spectroscopy
K_BIN_: binding constant
K_SV_: Stern–Volmer constant
MALDI-MS: matrix-assisted laser desorption/ionization mass spectrometry
MCL1: myeloid cell leukemia-1
MKN28 and MKN45: human gastric cancer cell lines
MitoSOX: mitochondrial superoxide indicators
MTDR: MitoTracker Deep Red
MTS: 3-(4,5-dimethylthiazol-2-yl)-5-(3-carboxymethoxyphenyl)-2-(4-sulfophenyl)-2H-tetrazolium
NAC: N-acethylcysteine
NMR: nuclear magnetic resonance
OC: circular open
OCT3/4: octamer-binding transcription factor 3/4
ORTEP: Oak Ridge Thermal Ellipsoid Plot
PARP: poly­(ADP-ribose) polymerase
PBS: phosphate-buffered saline
PET: polyethylene terephthalate
phe: phenylalanine
PDAC: Pancreatic Ductal Adenocarcinome
PI: propidium iodide
ppb: parts per billion
PVDF: polyvinylidene fluoride
RNase: ribonuclease
RNA-seq: RNA sequencing
ROS: reactive oxygen species
RT: room temperature
SD: standard deviation
SDS-PAGE: sodium dodecyl sulfate-polyacrylamide gel electrophoresis
STAT3: Signal Transducer and Activator of Transcription 3
TFA: trifluoroacetic acid
TGA: thermogravimetric analysis
TSP: sodium 3-(trimethylsilyl)­propionate
Tris: tris­(hydroxymethyl)­aminomethane
Trp: tryptophan
Tyr: tyrosine
UV: –Vis, ultraviolet–visible spectroscopy
ΔΨm: mitochondrial membrane potential
