# Supplementary material for: Chemical Stabilization and Transformation of Schiff Base Scaffold: A Path toward Superior Platinum-Based Anti-Cancer Stem Cell Drugs
Source: J Med Chem. 2026 Aug 14;69(17):20943–66. doi: 10.1021/acs.jmedchem.6c01871 (PMC13576395; doi:10.1021/acs.jmedchem.6c01871)
Supplement: Supplementary file 1 [file jm6c01871_si_002.pdf]

Chemical Stabilization and Transformation of Schiff Base Scaffold: A Path Toward Superior Platinum-Based Anti-Cancer Stem Cells drugs

Cynthia Sinaí Novoa Ramirez‡[a], *Jorge Melones Herrero*‡[b,c,d], *Susana Priego Gutierrez,*[b,c] *Leticia Cubo,*[a] *Adoración Gómez Quiroga* [a,e]* *and Isabel Sanchez Pérez**[b,c,d,f]

[a]Universidad Autónoma de Madrid (UAM), Department of Inorganic Chemistry, 28049 Madrid Spain [b]Department of Biochemistry. School of Medicine, UAM, 28029 Madrid, Spain. [c]Instituto de Investigaciones Biomédicas "Sols-Morreale" IIBM-CSIC-UAM, Madrid, Spain. [d]BIOPAC-IRYCIS [e] Institute of Advanced Chemical Sciences (IAdChem). UAM. 28049 Madrid. Spain. [f]Centro de Investigación Biomédica en Red, Área Rare Diseases, CIBERER, ISCIII, Madrid, Spain

Contents

Single crystal resolution method by X-Ray 2

Tables 3

NMR spectra of the ligands 4

Additional spectra for complex PtCl(L^1H)]Cl characterization 9

Additional spectra for complex [PtCl(L^1)] characterization 15

UV-Vis spectra for the stability studies of L^1H and $L^{1.}$ 19

UV-Vis spectra for the stability studies of PtCl(L^1H)]Cl and [PtCl(L^1)] 20

Supplementary graphs and data supporting biological studies performed with PtCl(L^1H)]Cl and [PtCl(L^1)]. 26

All uncropped blots data 28

Single crystal resolution method by X-Ray

Two crystals were obtained from a methanolic solution of **[PtCl(L^1H)]Cl** during crystallization: the neutral species **[PtCl(L^1)]**, arising from deprotonation of one of the nitrogen atoms of the oxidized tridentate ligand **L^1** and the cationic complex **[PtCl(L^1)]Cl**, in which the ligand is oxidized**.**

The diffraction pattern was indexed, and the total number of runs and images was based on the strategy calculation from the program CrysAlisPro (Rigaku) and the unit cell was refined using CrysAlisPro (Rigaku, V1.171.43.121a, 2024) on 16997 reflections, 70% of the observed reflections.

Data reduction, scaling and absorption corrections were performed using CrysAlisPro (Rigaku, V1.171.43.121a, 2024). The final completeness is 99.70% out to 68.225° in Q. A gaussian absorption correction was performed using CrysAlisPro 1.171.43.121a (Rigaku Oxford Diffraction, 2024) Numerical absorption correction based on gaussian integration overall multifaceted crystal mode Empirical absorption correction using spherical harmonics, implemented in SCALE3 ABSPACK scaling algorithm. The absorption coefficient m of this material is 20.905 mm^{-1} at this wavelength (l= 1.542Å) and the minimum and maximum transmissions are 0.272 and 0.711.

For **[PtCl(L^1)]Cl**, the structure was solved and the space group *P*-1 (# 2) determined by the ShelXT (Sheldrick, 2015) structure solution program using Intrinsic Phasing and refined by Least Squares using version 2018/3 of ShelXL 2018/3 (Sheldrick, 2015). All non-hydrogen atoms were refined anisotropically. Hydrogen atom positions were calculated geometrically and refined using the riding model. For **[PtCl(L^1)]**, the structure was solved and the space group *P*4/*mnc* (# 128) determined by the XT[19] structure solution program using Intrinsic Phasing and refined by Least Squares using version 2018/3 of ShelXL 2018/3 (Sheldrick, 2015). All non-hydrogen atoms were refined anisotropically. Hydrogen atom positions were calculated geometrically and refined using the riding model, but some hydrogen atoms were refined freely. The crystallographic data for the structure reported has been deposited with the Cambridge Crystallographic Data Centre as supplementary publication no. CCDC 04112 and 04531. Copies of the data can be obtained free of charge on application to CCDC, 12 Union Road, Cambridge, CB2 1EZ, UK (fax: (+44) 1223-336-033, e-mail: deposit@ccdc.cam.ac.uk). The information is also enclosed in Table SI 1 for best convenience.

Tables

*Table SI 1. Crystal data and structure refinement for **[PtCl(L¹)]Cl** and **[PtCl(L¹)]**.*

Compound	**[PtCl(L¹)Cl]•H_2O (CCDC 04531)**	**[PtCl(L¹)]·$(H_2O)_{0.25}$ (CCDC 04112)**
Formula	$C_{13}H_{15}Cl_2N_3OPt$	$C_{13}H_{12}ClN_3O_{0.25}Pt$
Dcalc./ g cm^{-3}	2.246	2.162
μ/mm^{-1}	21.268	20.905
Formula Weight	495.27	444.80
Color	Clear red	intense purple
Shape	plate	ribbon
Size/mm^3	0.10×0.05×0.02	0.08×0.03×0.02
T/K	275.02(10)	250.00(10)
Crystal System	triclinic	tetragonal
Space Group	P-1	P4/mnc
a/Å	9.4340(2)	20.43812(13)
b/Å	9.7706(2)	20.43812(13)
c/Å	10.2157(2)	6.54275(7)
α/°	114.163(2)	90.0
β/°	96.0689(18)	90.0
γ/°	115.078(2)	90.0
V/$Å^3$	732.39(3)	2733.02(4)
Z	2	8
Z'	1	0.5
Wavelength/Å	1.54184	1.54184
Radiation type	Cu K_a	Cu K_a
Qmin/°	5.042	3.058
Qmax/°	68.239	68.225
Independent Refl.	13734	1368
Reflections with I > 2(I)	2632	1325
R_{int}	0.0200	0.0319
Parameters	196	114
Restraints	14	1
Largest Peak	1.551	1.379
Deepest Hole	-1.037	-0.715
GooF	1.106	1.168
wR_2 (all data)	0.0471	0.0781
wR_2	0.0469	0.0772
R_1 (all data)	0.0198	0.0247

Table SI 2. Percentage of cells in each Cell Cycle Phase after 24 h treatments.

	G0/G1	S	G2/M
**Control**	48.95±2.94	24.82±1.28	26.24±4.10
**CDDP** 10 μM	23.54±2.80	55.67±3.60	20.79±5.89
**L¹** 25 μM	50.60±0.84	18.30±3.66	31.09±3.66
**L¹** 50 μM	57.76±7.00	17.77±7.82	23.97±5.83
**[PtCl(L¹)]** 25 μM	40.10±1.90	12.49±3.69	47.41±4.20
**[PtCl(L¹)]**50 μM	50.48±6.14	40.22±8.66	9.31±3.45
**L¹H** 5 μM	52.40±2.76	19.15±2.98	28.48±1.91
**L¹H** 20 μM	60.89±1.35	21.48±2.52	17.64±3.33
**[PtCl(L¹H)]Cl** 5 μM	43.75±6.35	19.73±3.56	36.50±3.68
**[PtCl(L¹H)]Cl** 20 μM	33.73±1.74	54.07±2.50	9.96±4.92

NMR spectra of the ligands

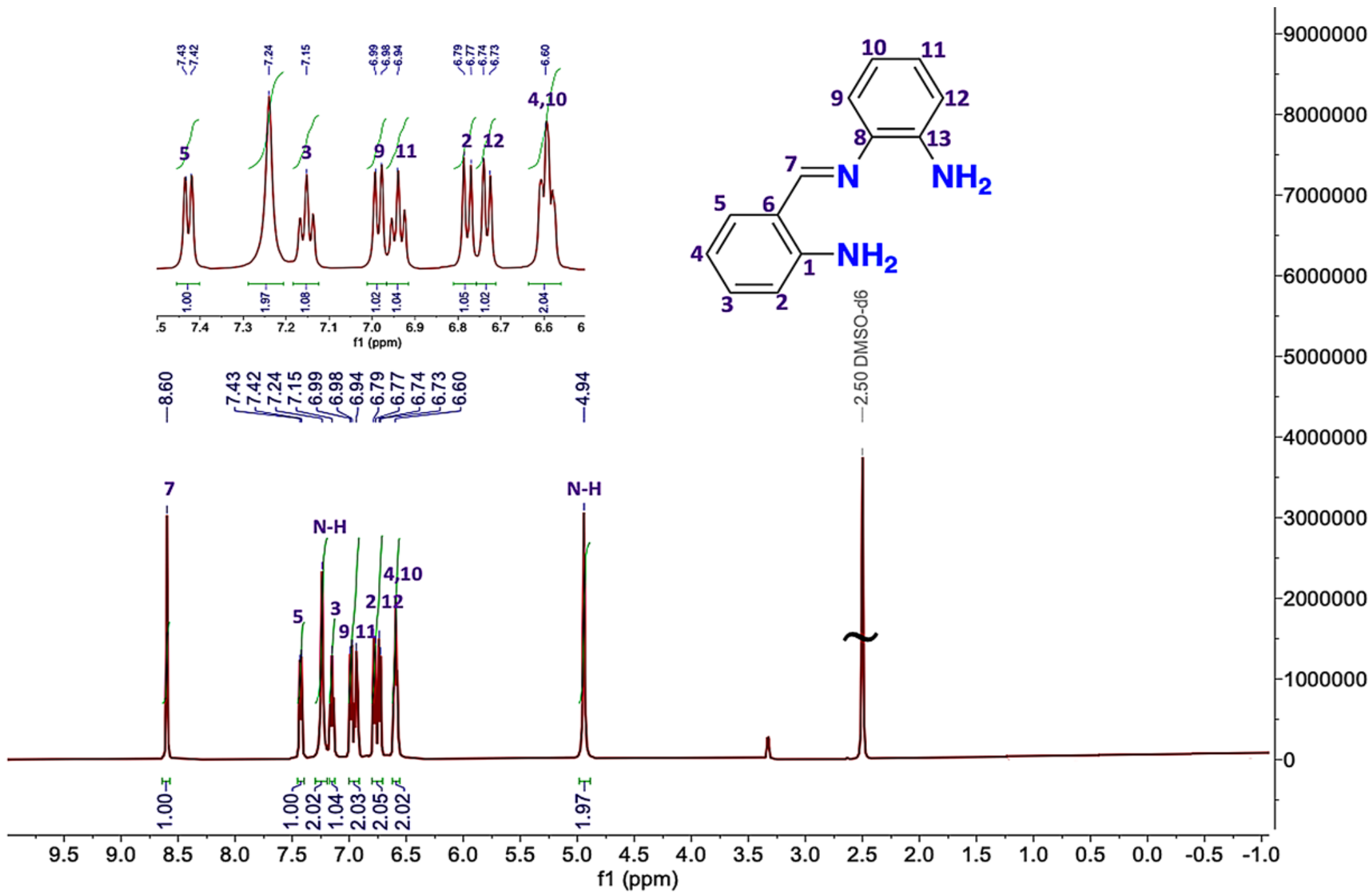

*Figure SI 1. ^{1}H NMR spectrum of **L[1]** at 25 °C in DMSO-d₆.*

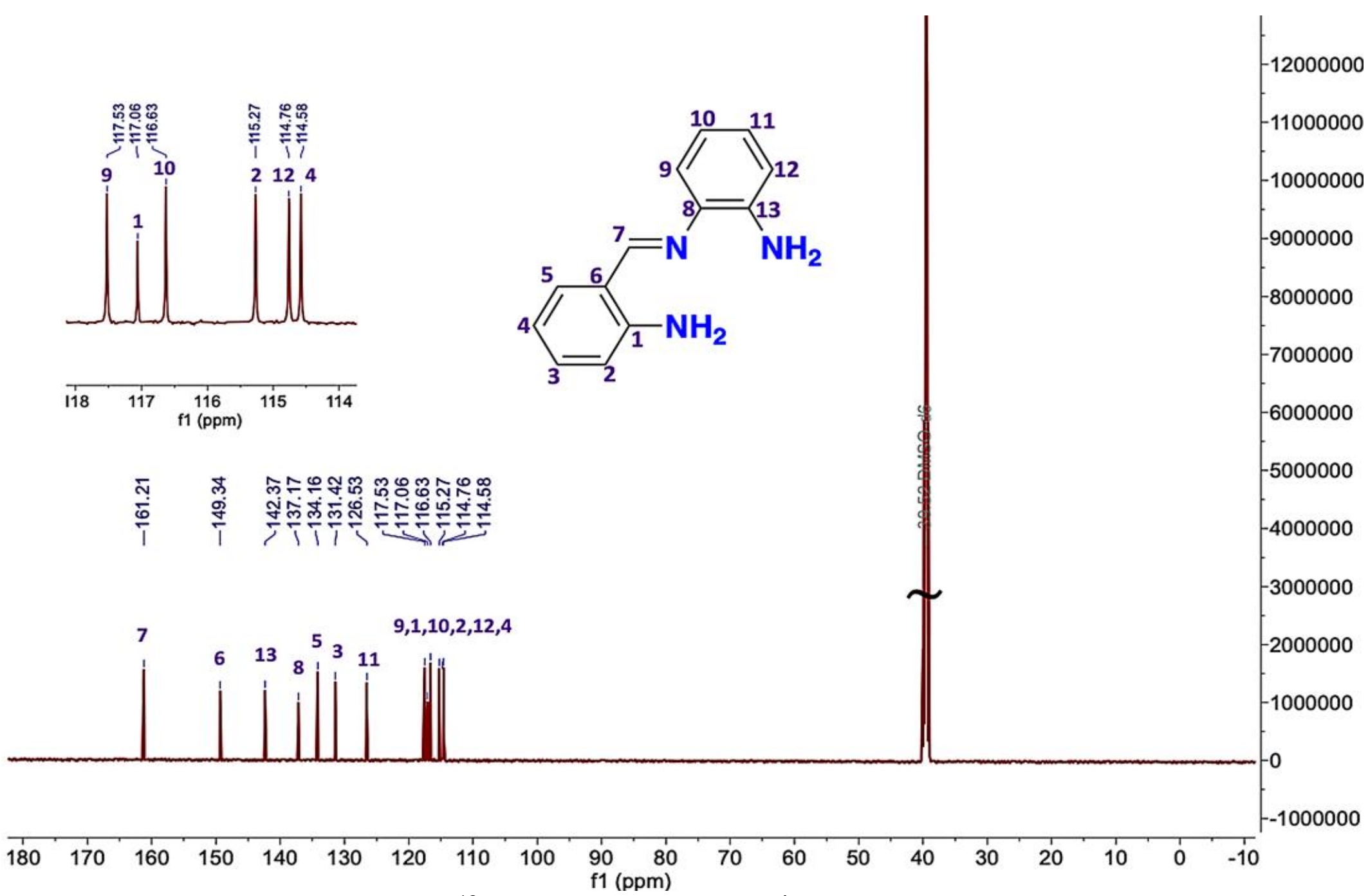

*Figure SI 2. ^{13}C NMR spectrum of **L[1]** at 25 °C in DMSO-d₆.*

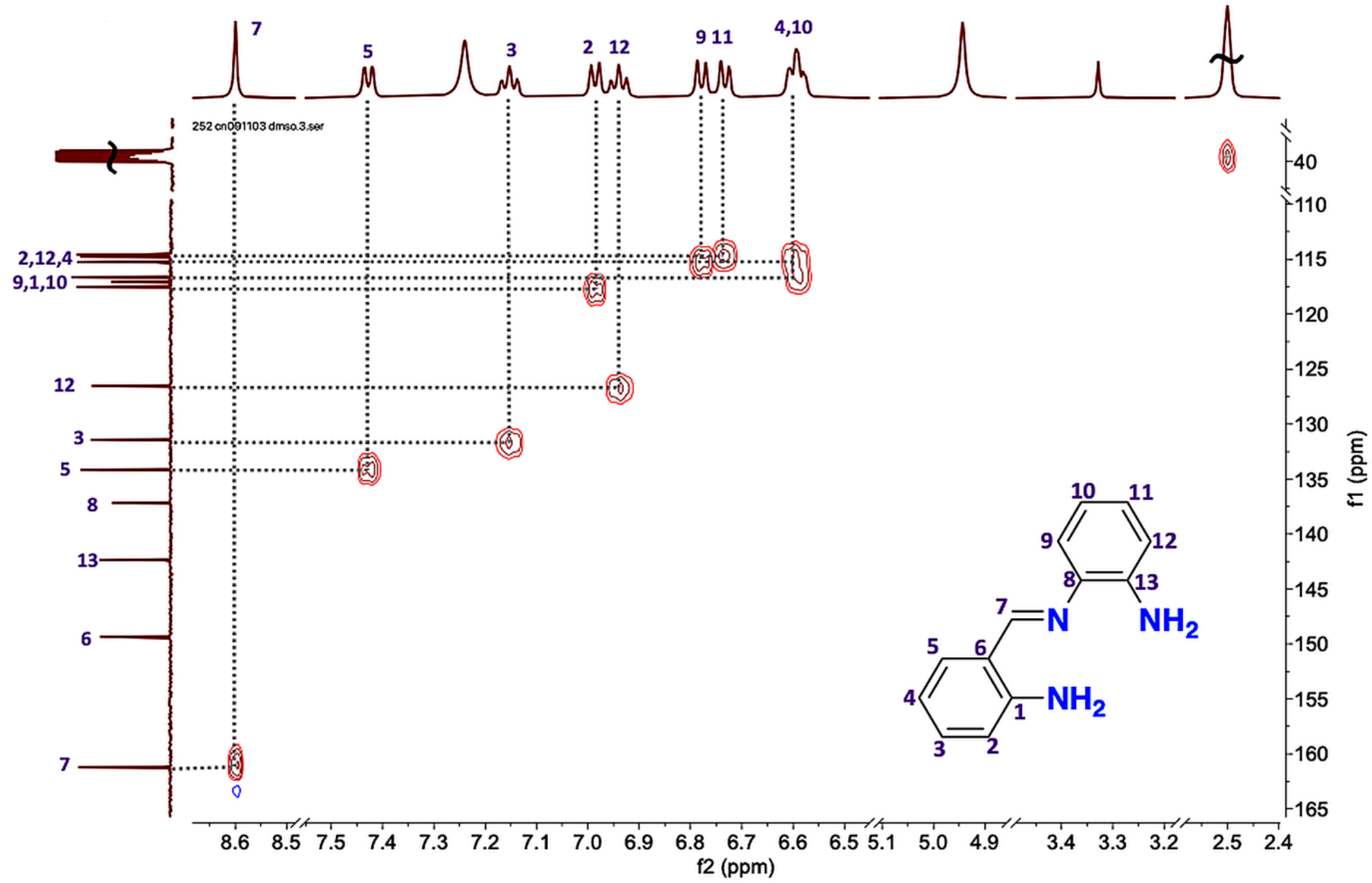

*Figure SI 3. 2D [¹H, ¹³C] HSQC NMR spectrum **L¹** at 25 °C in DMSO-d_6, showing only the signals corresponding to the observed correlations.*

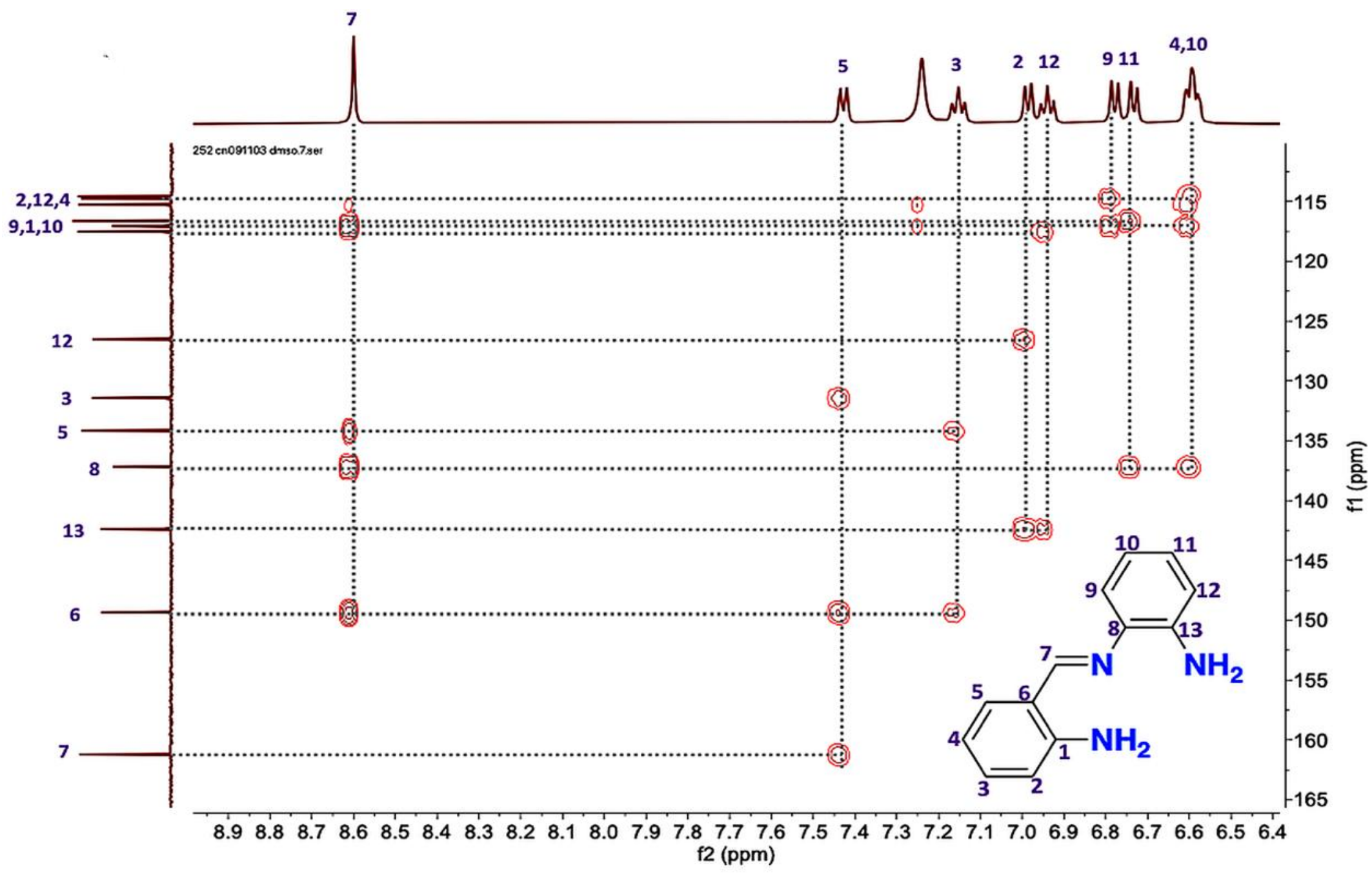

*Figure SI 4. 2D [¹H, ¹³C] HMBC NMR spectrum **L¹** at 25 °C in DMSO-d_6, showing only the signals corresponding to the observed correlations.*

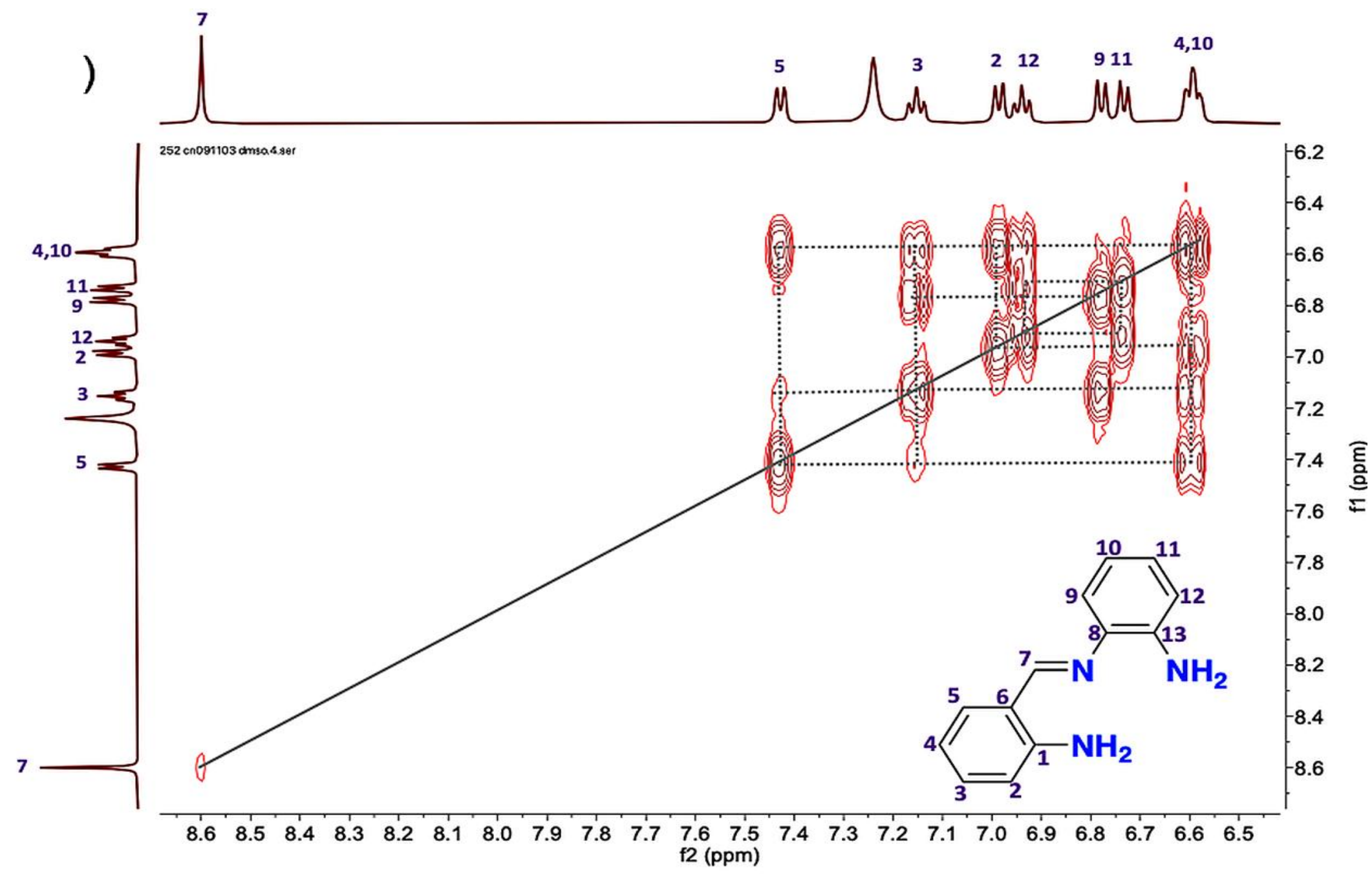

*Figure SI 5. 2D [^{1}H, ^{1}H] COSY NMR spectrum of **L^1** at 25 °C in DMSO-d$_6$, showing only the signals corresponding to the observed correlations.*

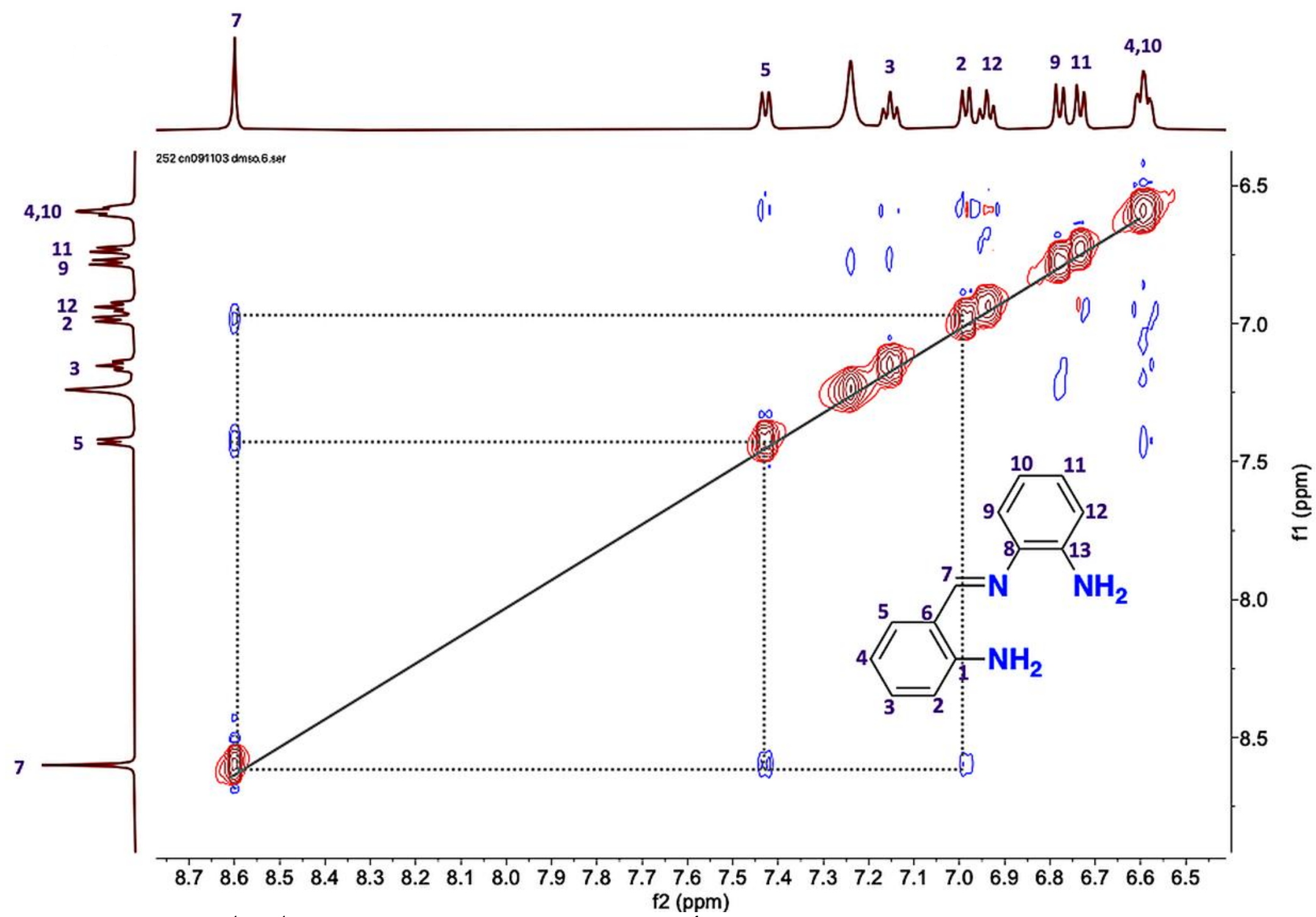

*Figure SI 6. 2D [^{1}H, ^{1}H] NOESY NMR spectrum of **L^1** at 25 °C in DMSO-d$_6$, showing only the signals corresponding to the observed correlations.*

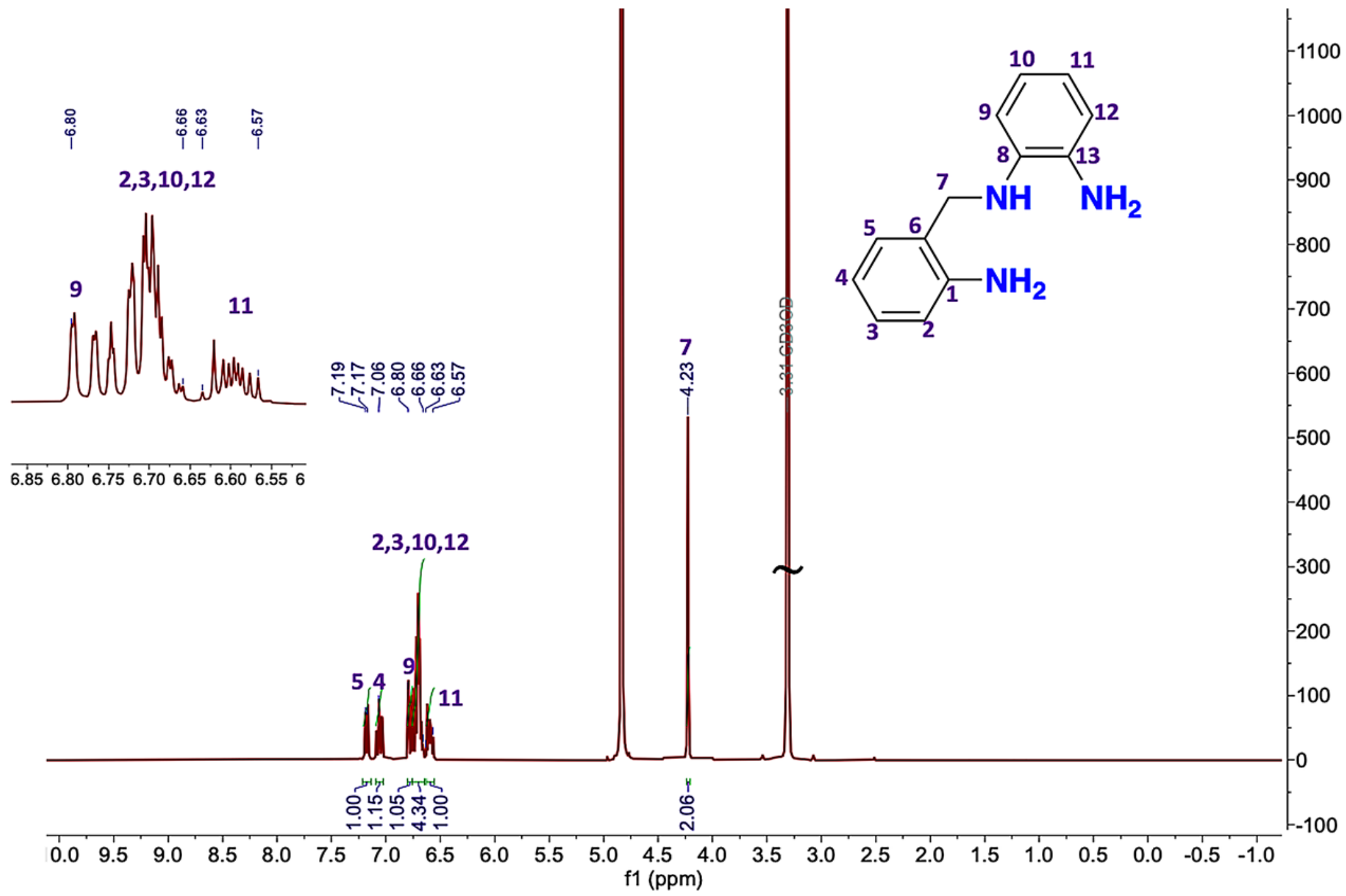

*Figure SI 7. ^{1}H NMR spectrum of **$L^{1}H$** at 25 °C in MeOH-d$_4$.*

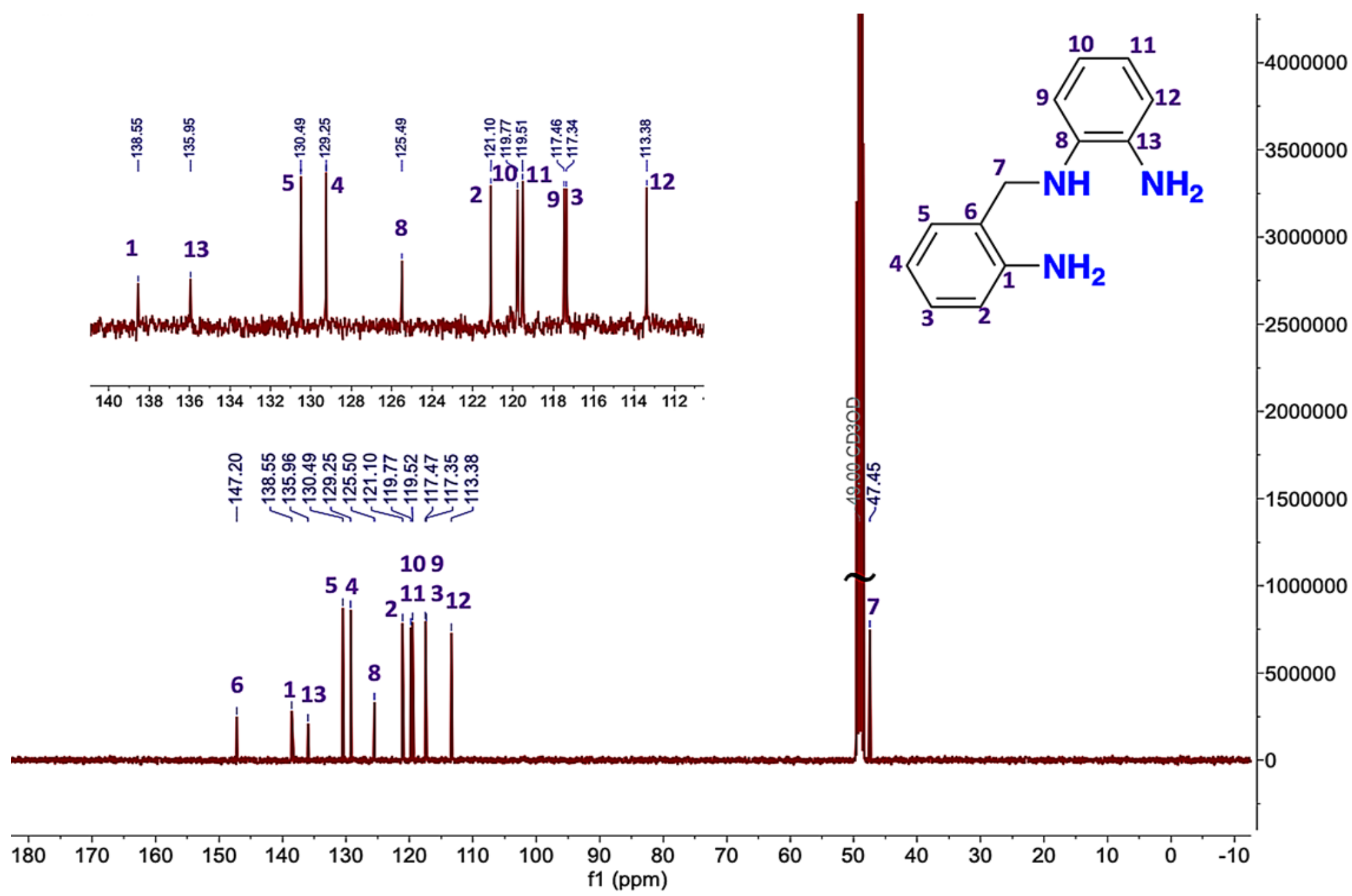

*Figure SI 8. ^{13}C NMR spectrum of **$L^{1}H$** at 25 °C in MeOH-d$_4$.*

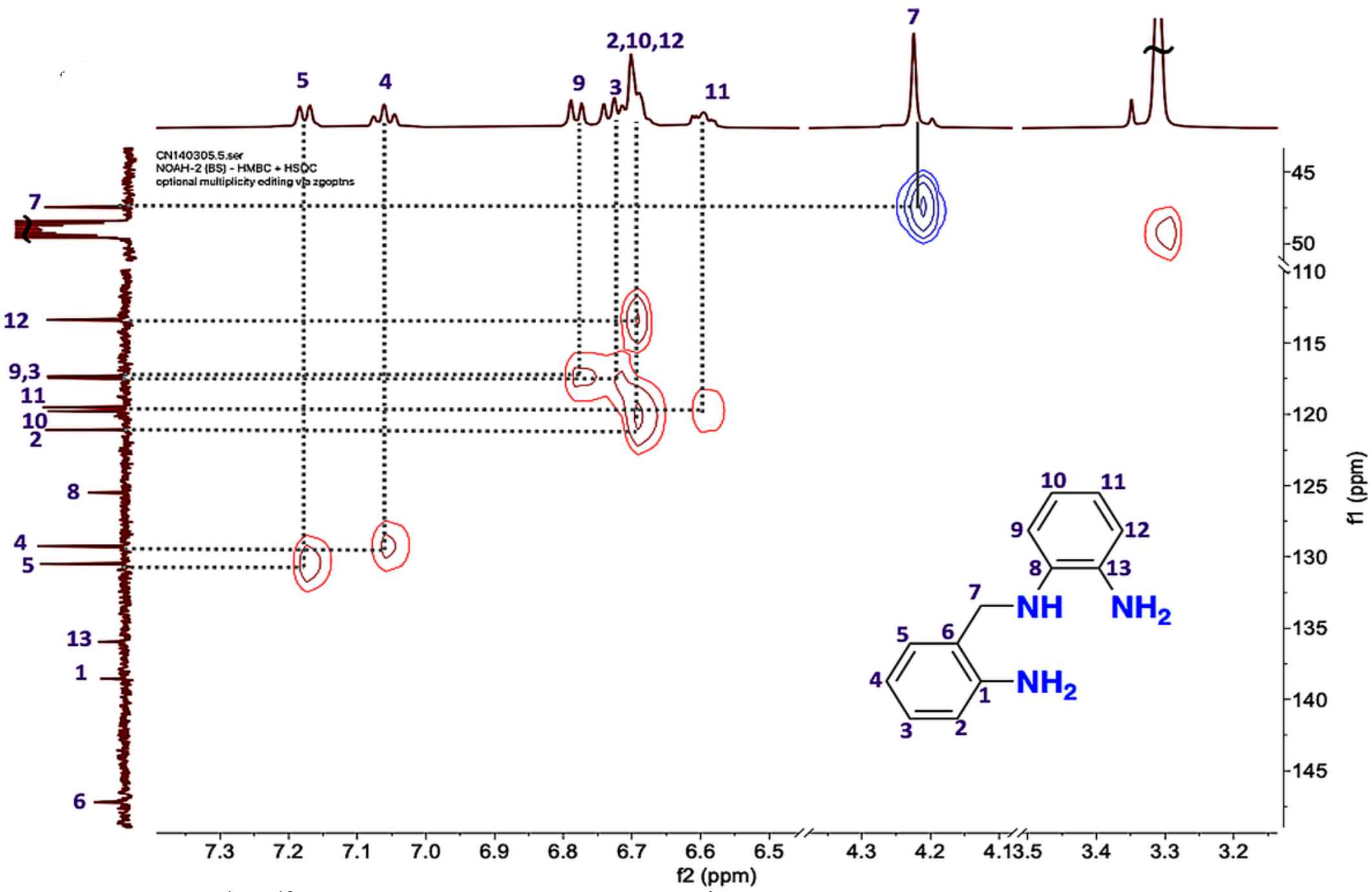

*Figure SI 9. 2D [^{1}H, ^{13}C] HSQC NMR spectrum of **L^1H** at 25 °C in MeOH-d$_4$, showing only the signals corresponding to the observed correlations.*

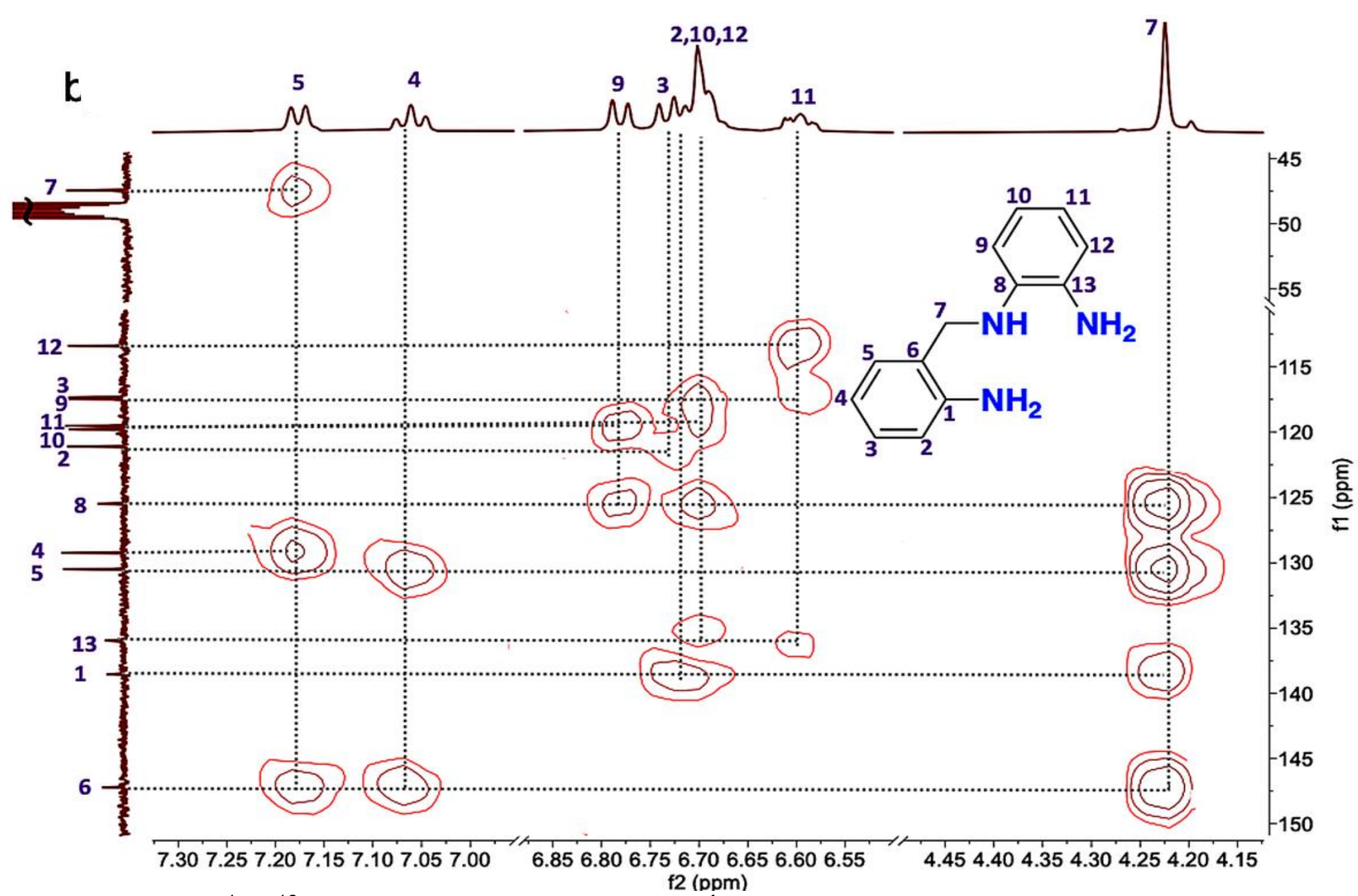

*Figure SI 10. 2D [^{1}H, ^{13}C] HMBC NMR spectrum of **L^1H** at 25 °C in MeOH-d$_4$, showing only the signals corresponding to the observed correlations.*

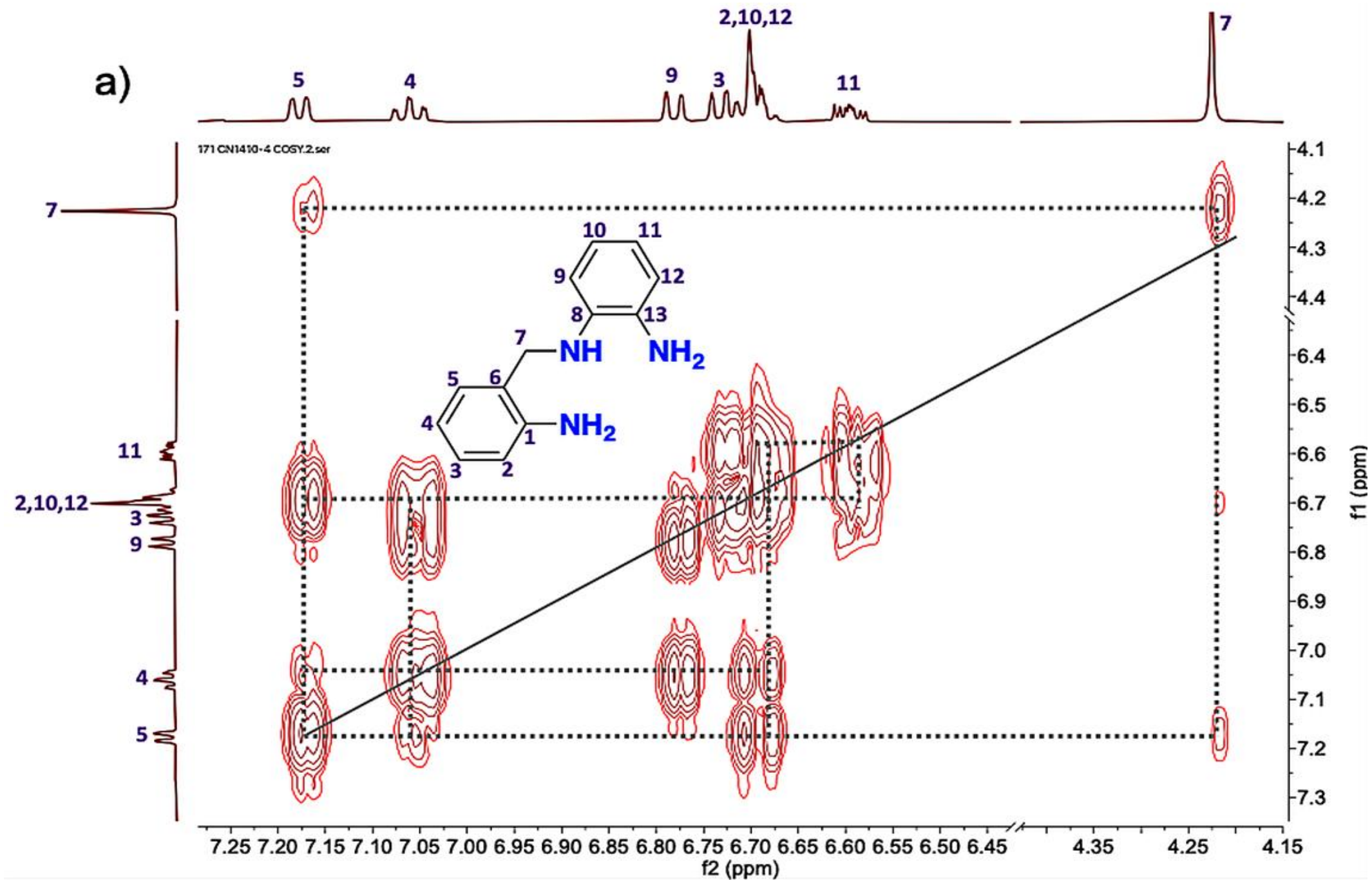

Figure SI 11. 2D [^{1}H, ^{1}H] COSY NMR spectrum of ligand ***L^{1}H*** *at 25 °C in MeOH-d$_4$, showing only the signals corresponding to the observed correlations.*

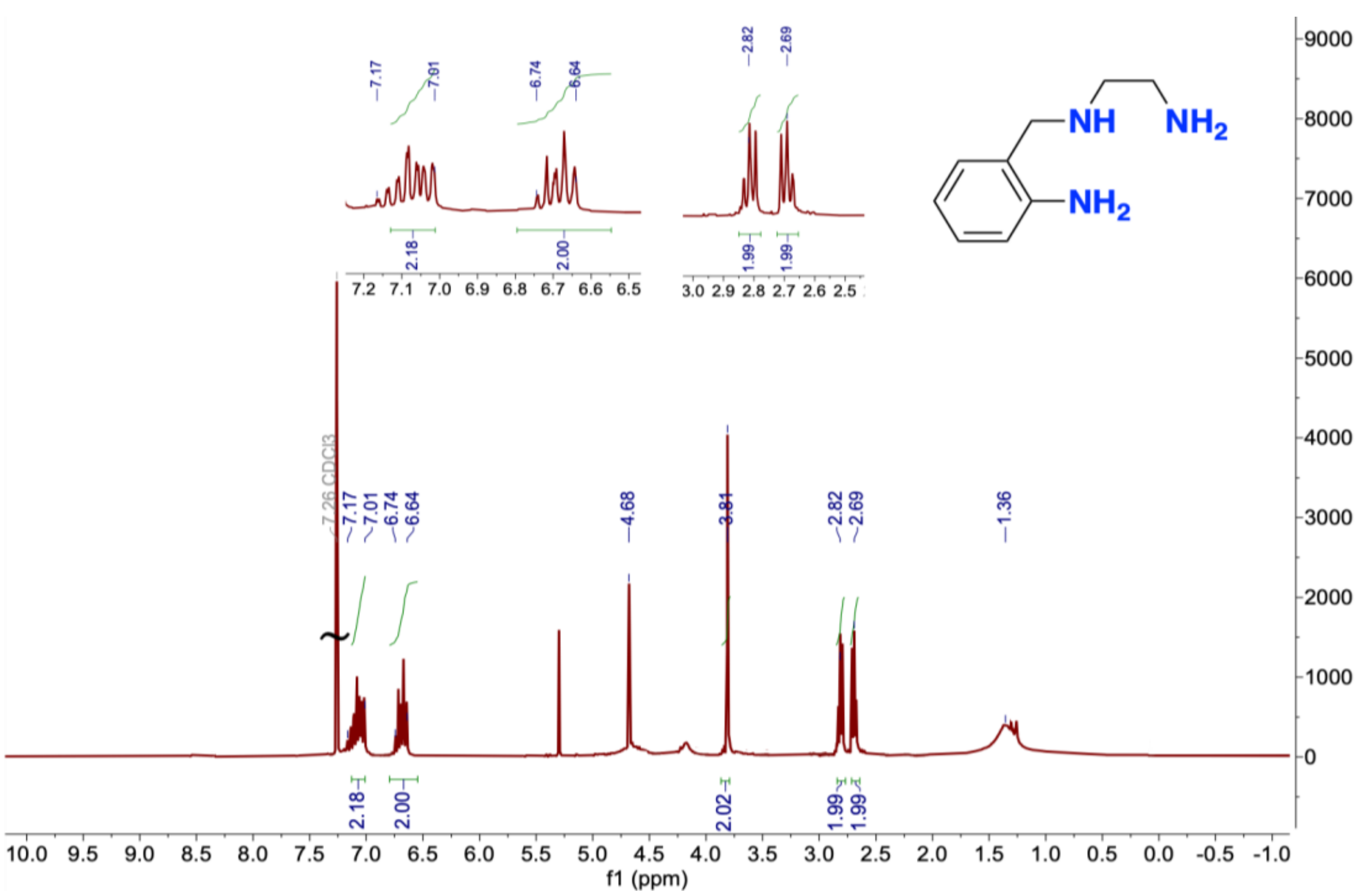

Figure SI 12. ^{1}H NMR spectrum of ***L^{2}H*** *at 25 °C in CHCl$_3$-d.*

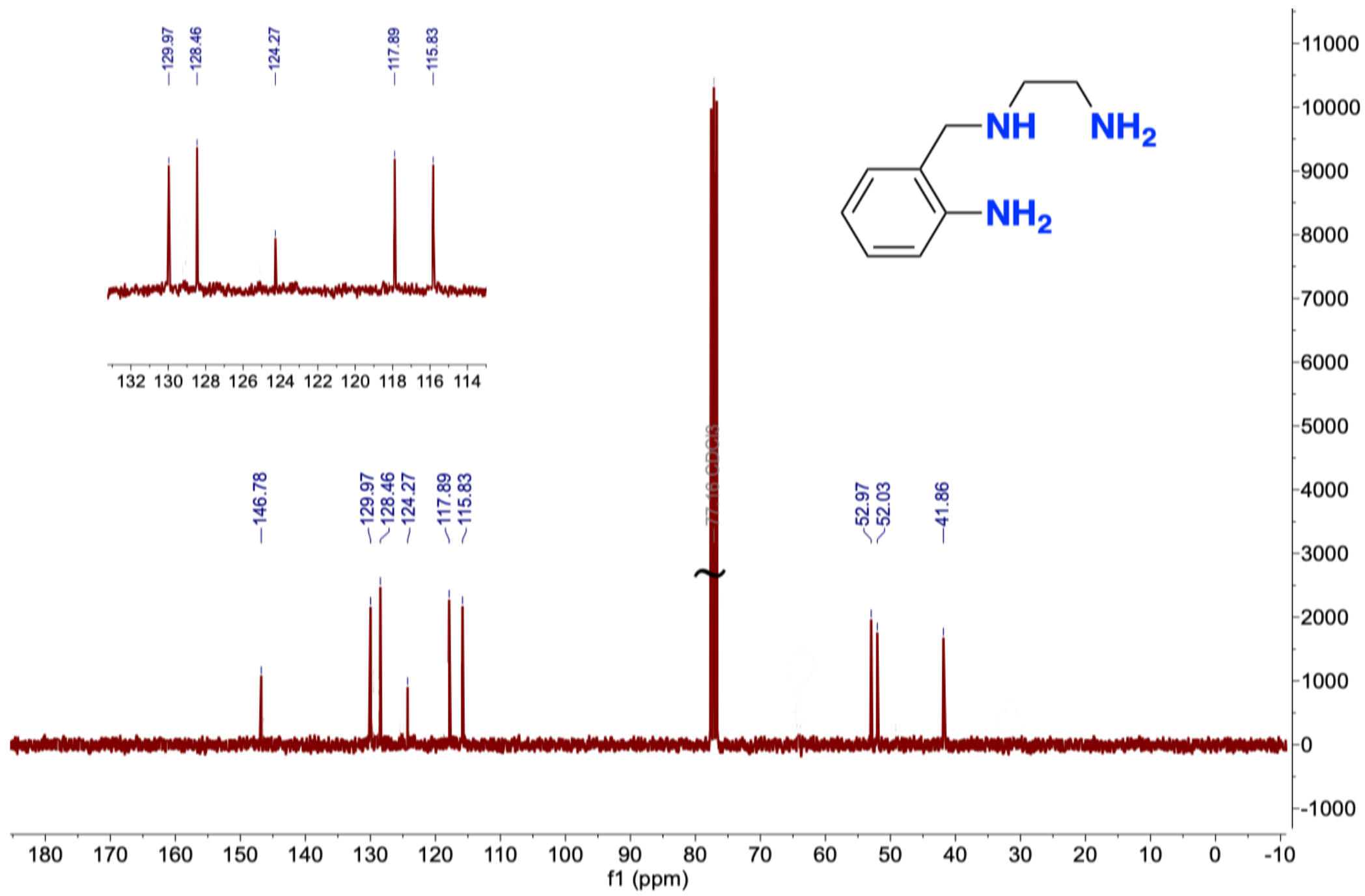

*Figure SI 13. ^{13}C NMR spectrum of **L^{2}H** at 25 °C in CHCl$_3$-d.*

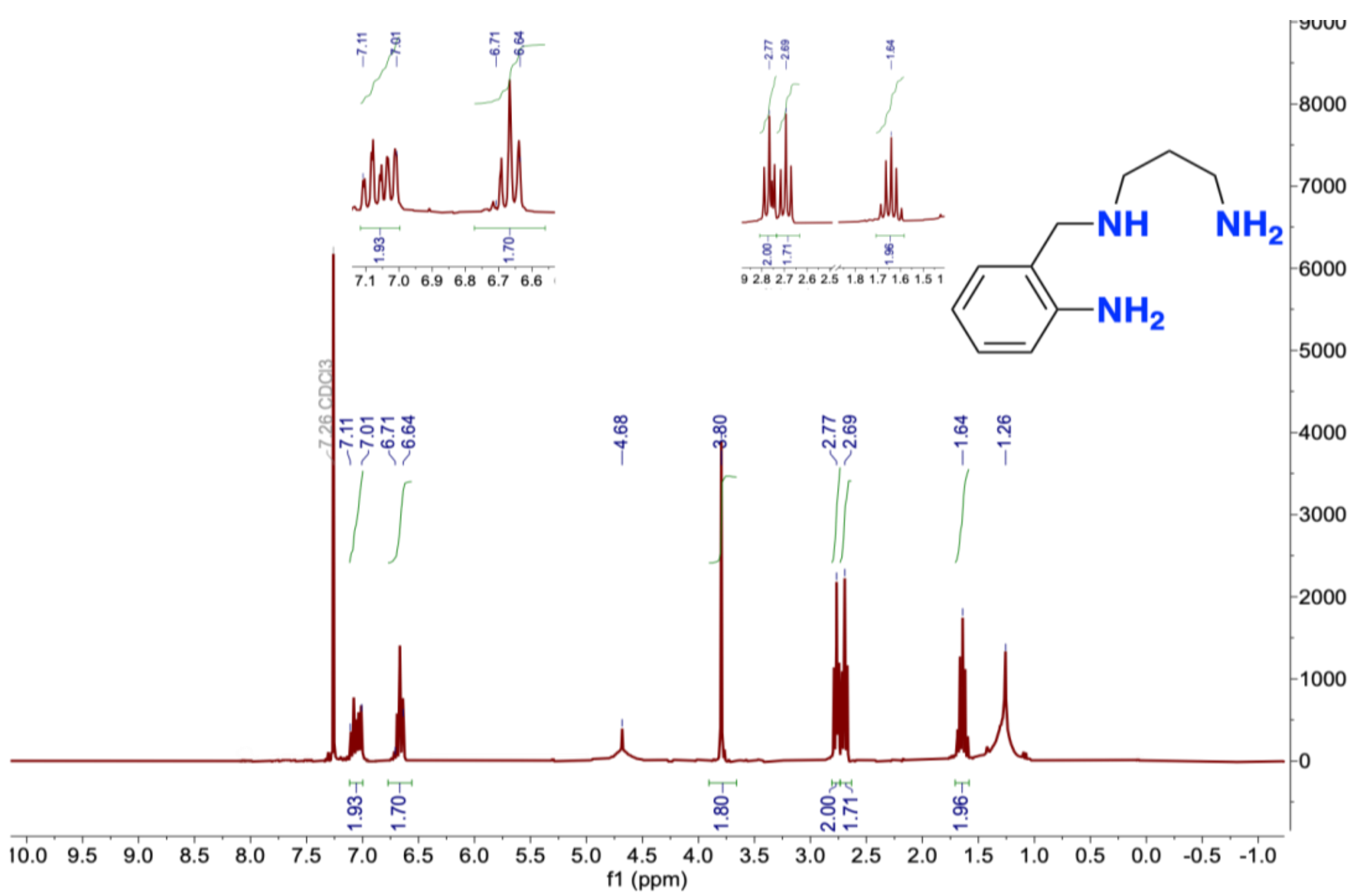

*Figure SI 14. ^{1}H NMR spectrum of **L^{3}H** at 25 °C in CHCl$_3$-d.*

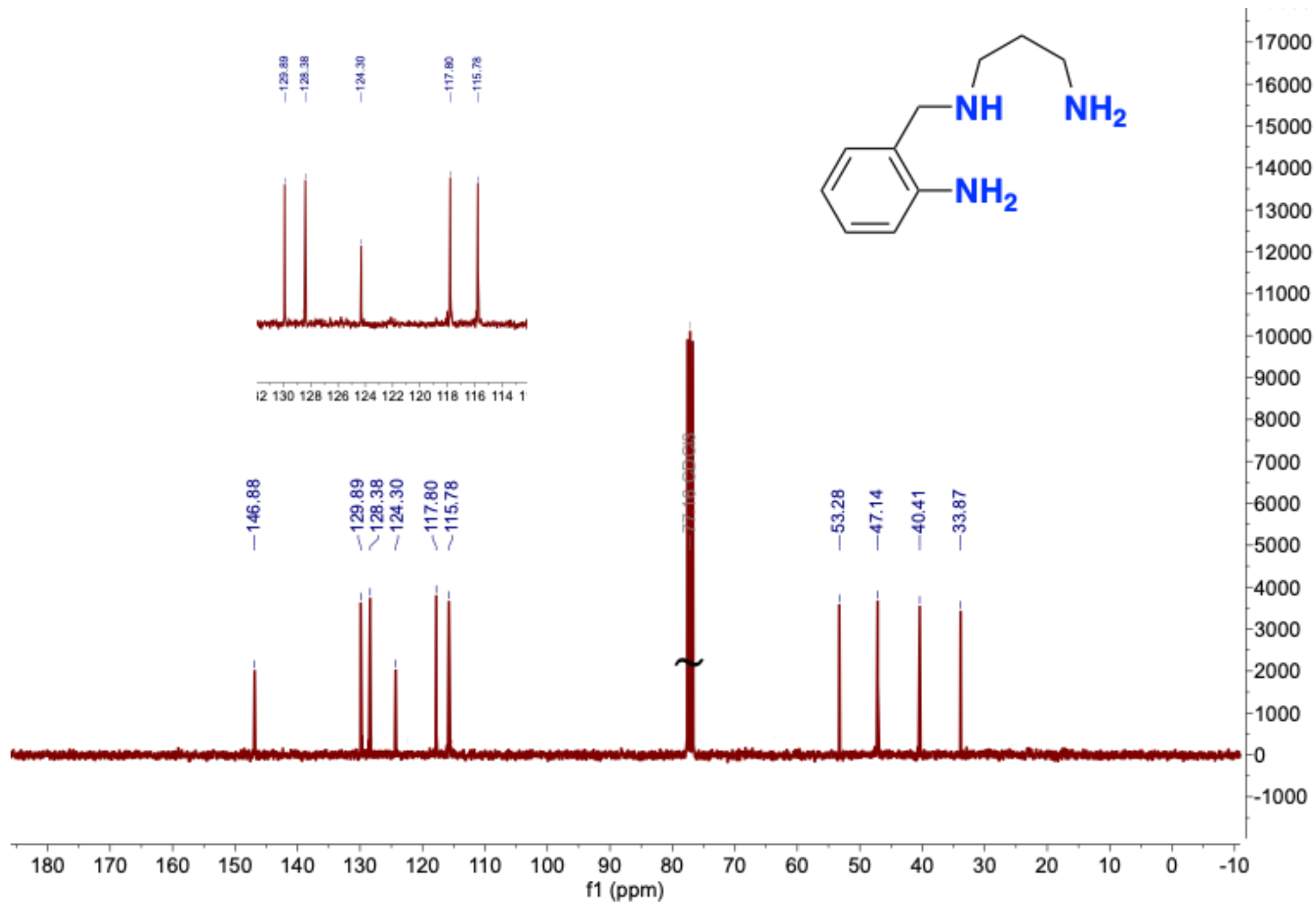

*Figure SI 15. ^{13}C NMR spectrum of **L^3H** at 25 °C in $CHCl_3$-d_6.*

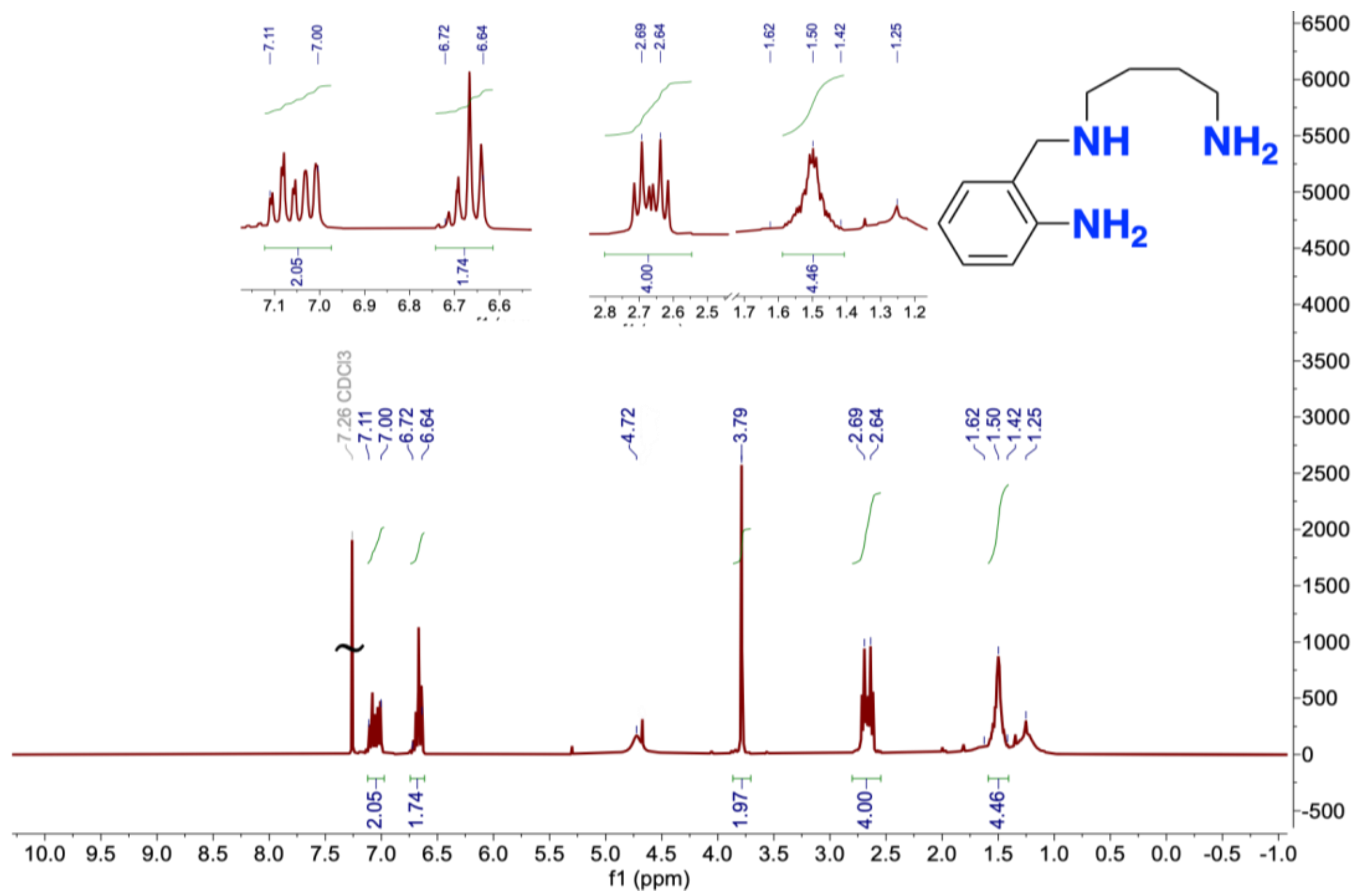

*Figure SI 16. ^{1}H NMR spectrum of **L^4H** at 25 °C in $CHCl_3$-d.*

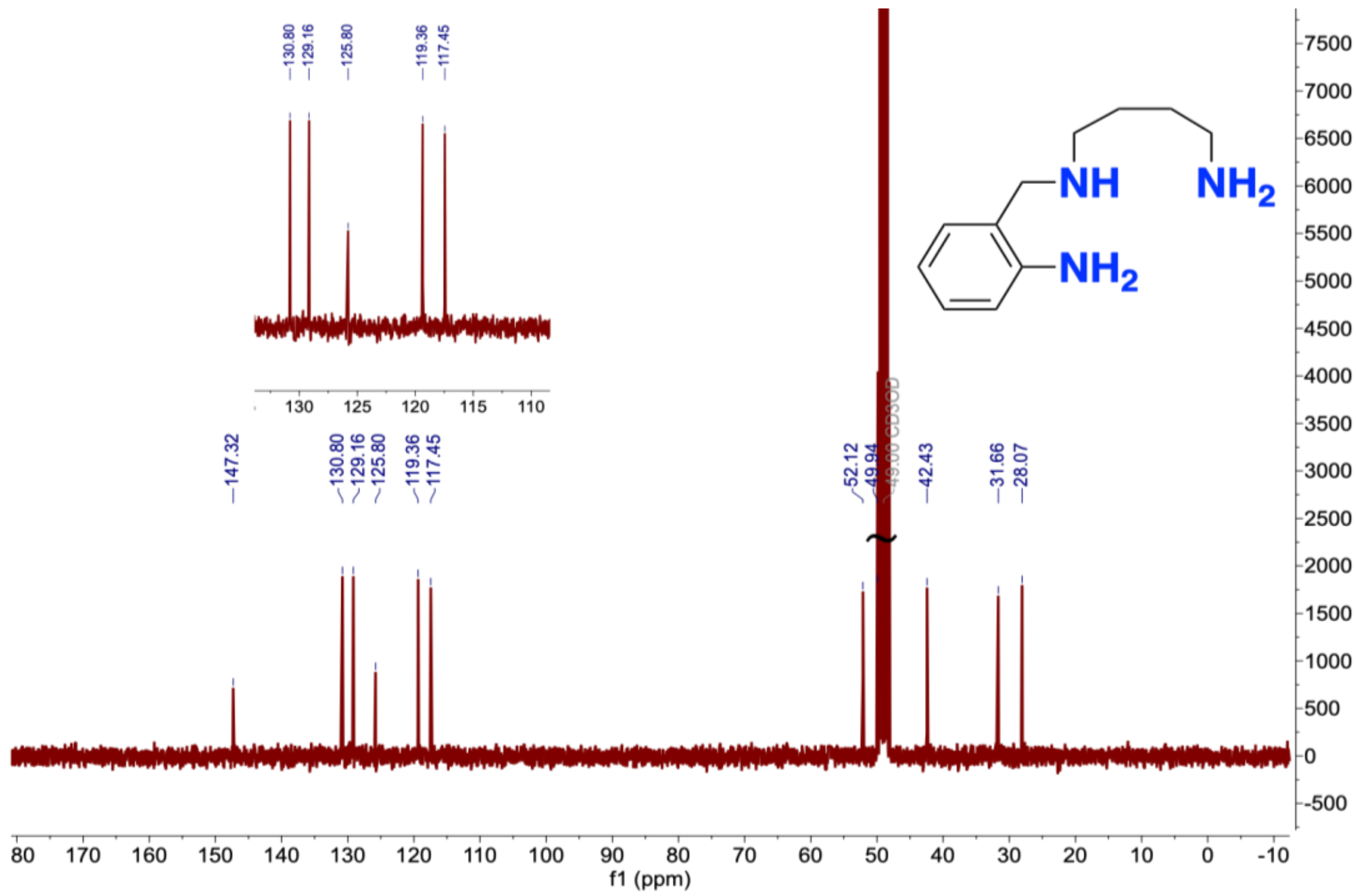

*Figure SI 17. ^{13}C NMR spectrum of **L⁴H** at 25 °C in MeOH-d₄.*

Additional spectra for complex PtCl(L[1]H)]Cl characterization

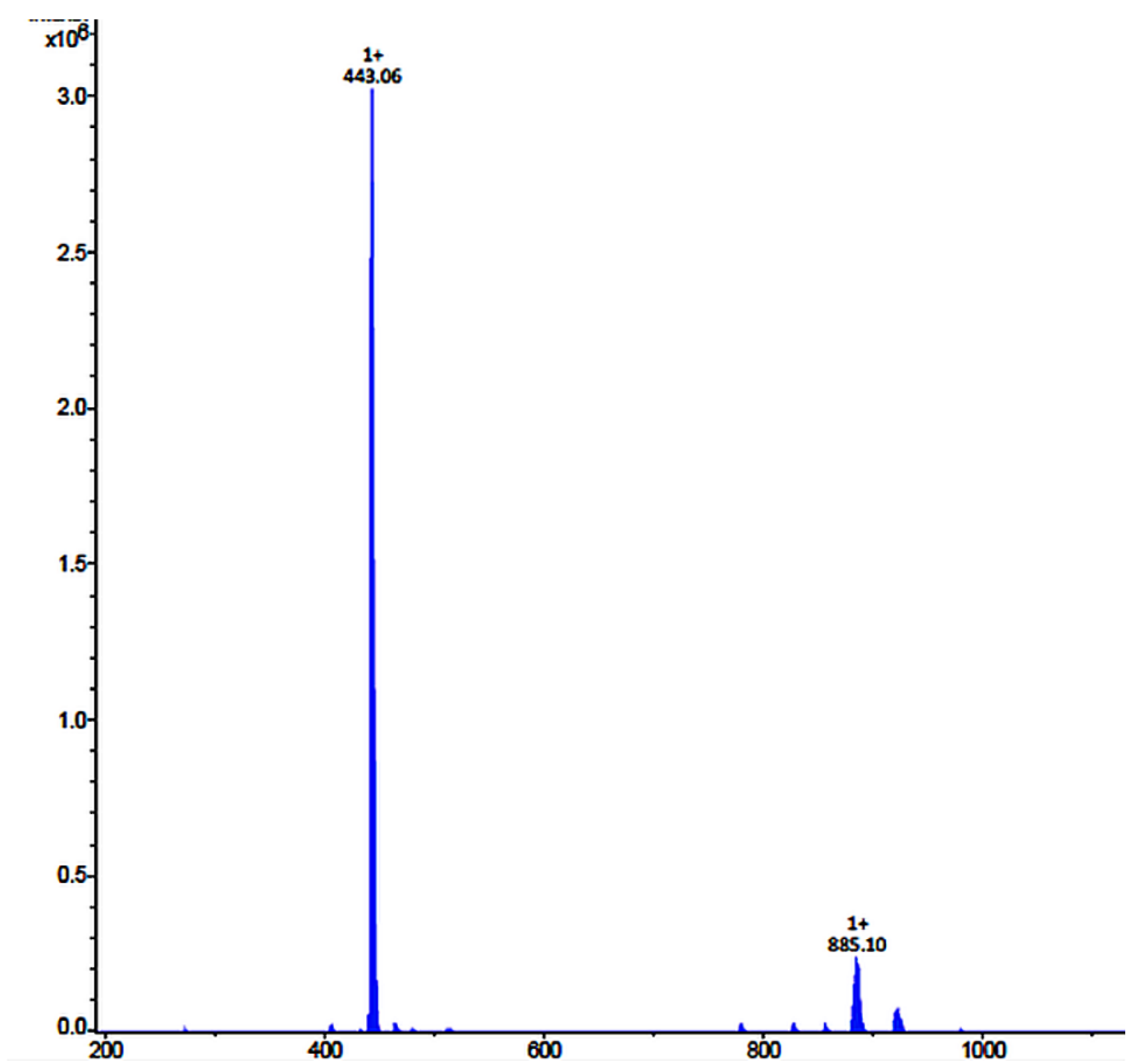

*Figure SI 18. ESI-MS+ of complex **[PtCl(L[1]H)]Cl**.*

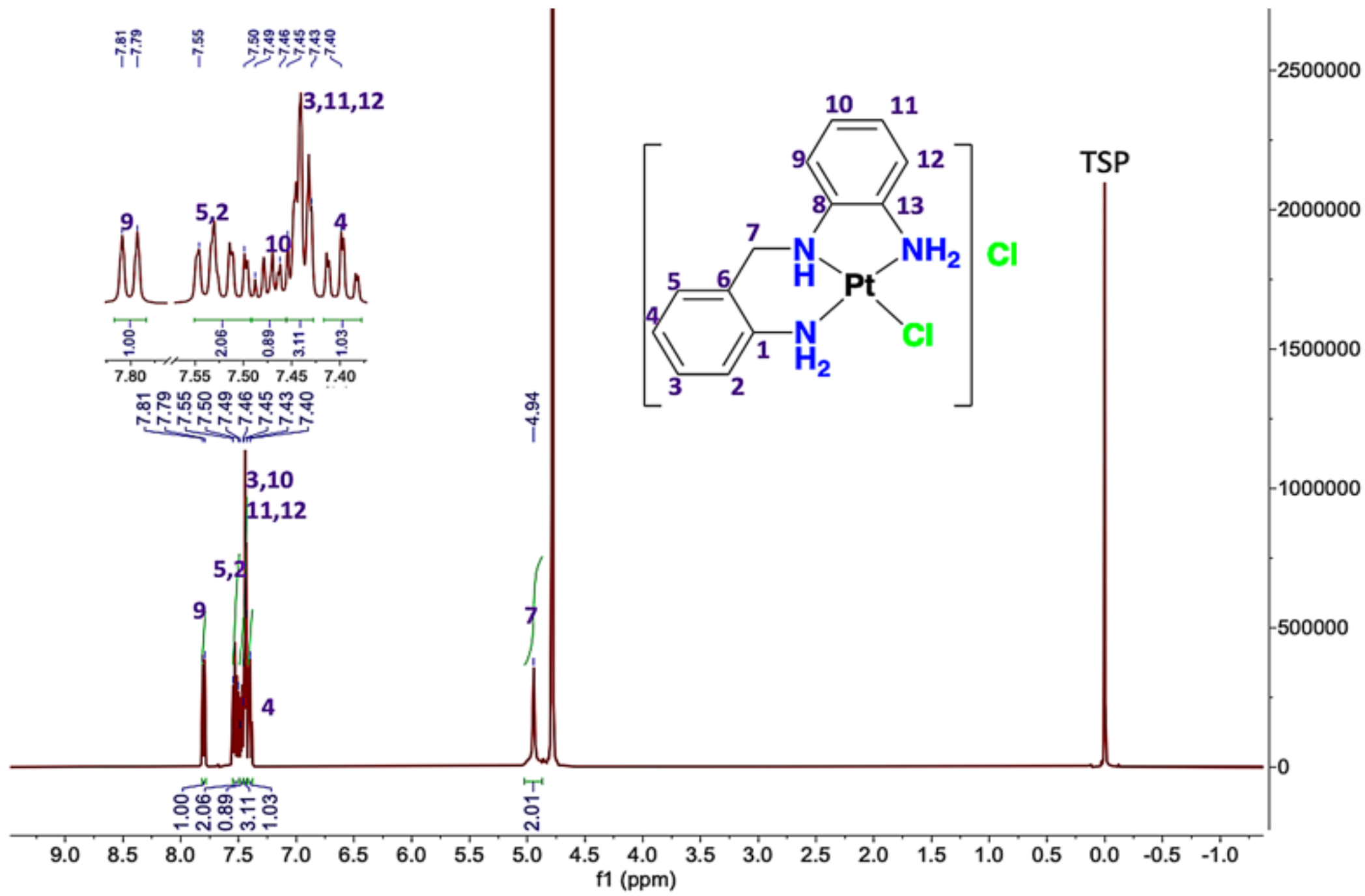

*Figure SI 19. ^{1}H NMR spectrum of complex **[PtCl(L^1H)]Cl** at 25 °C in D_2O+TSP.*

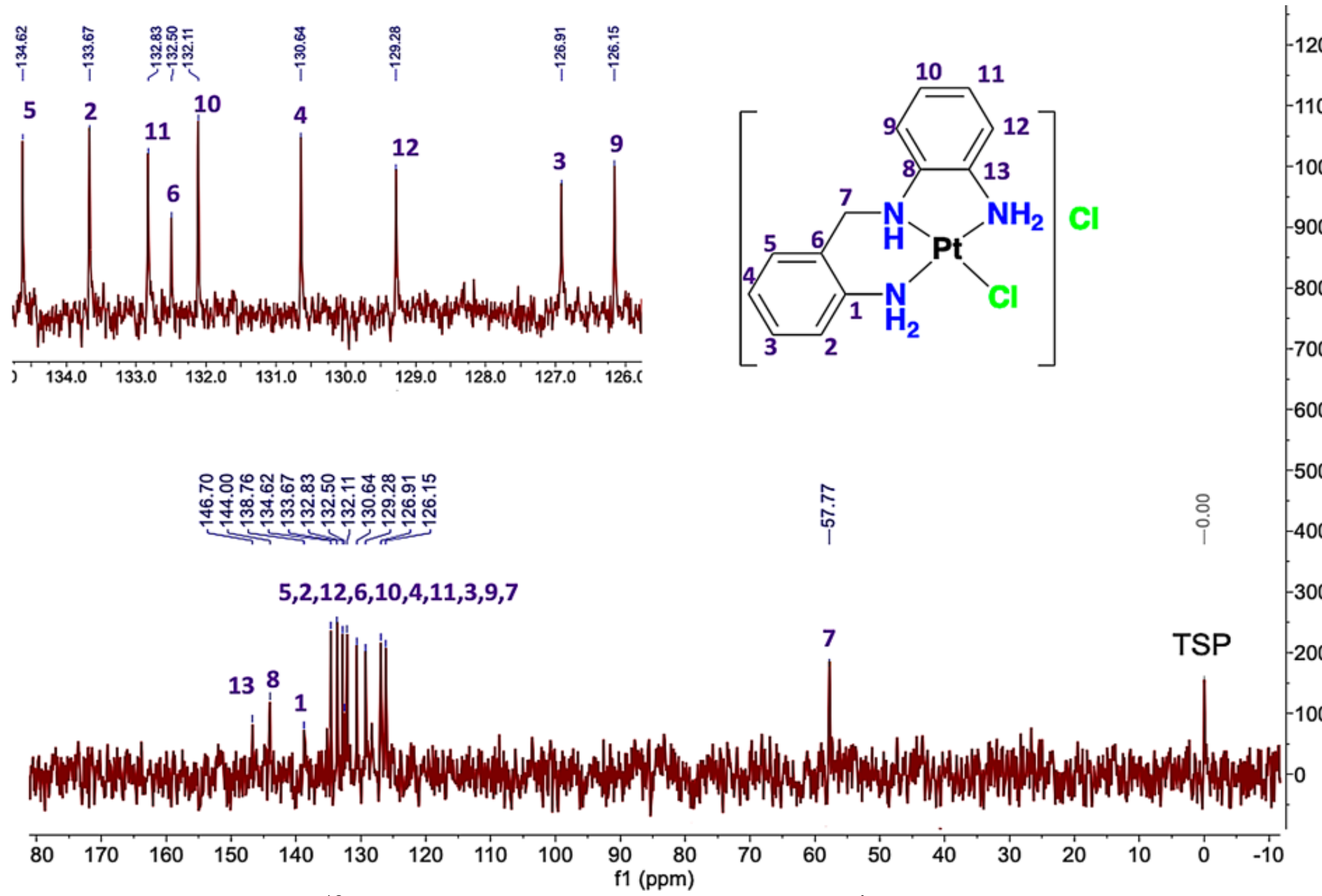

*Figure SI 20. ^{13}C NMR spectrum of complex **[PtCl(L^1H)]Cl** at 25 °C in D_2O +TSP.*

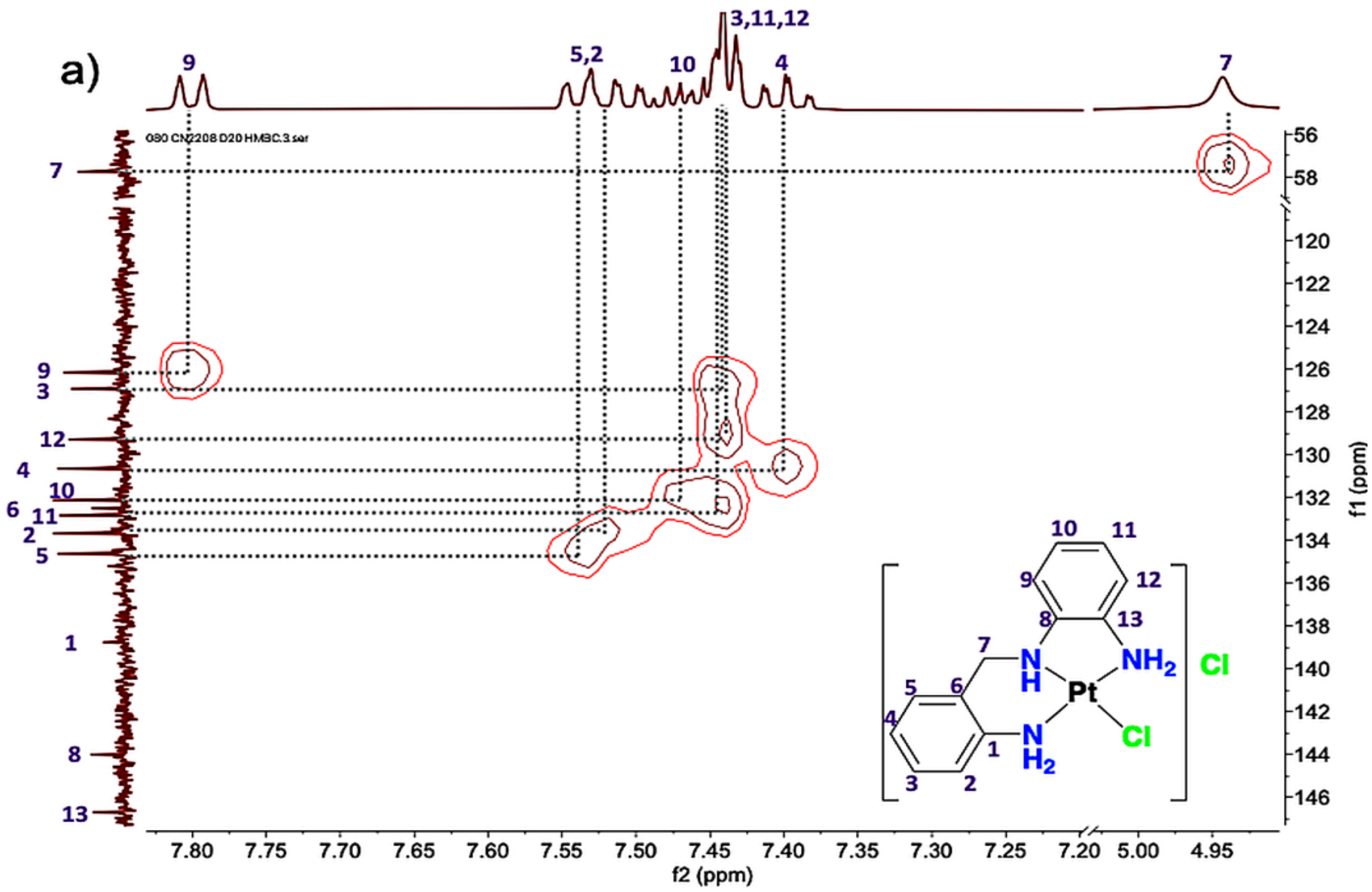

*Figure SI 21. 2D [^{1}H, ^{13}C] HSQC NMR spectrum of complex **[PtCl(L^{1}H)]Cl** at 25 °C in D_2O+TSP, showing only the signals corresponding to the observed correlations.*

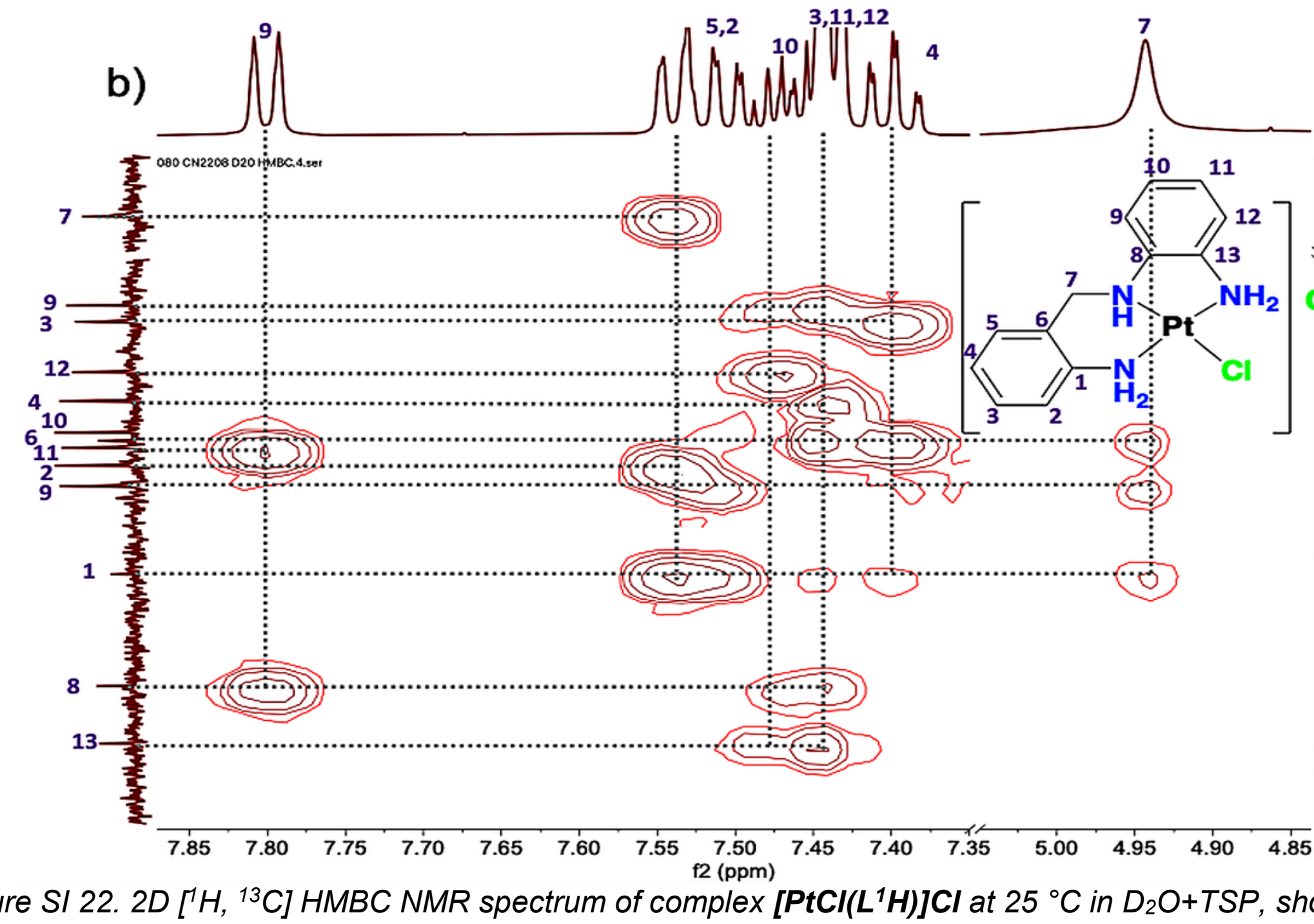

*Figure SI 22. 2D [^{1}H, ^{13}C] HMBC NMR spectrum of complex **[PtCl(L^{1}H)]Cl** at 25 °C in D_2O+TSP, showing only the signals corresponding to the observed correlations.*

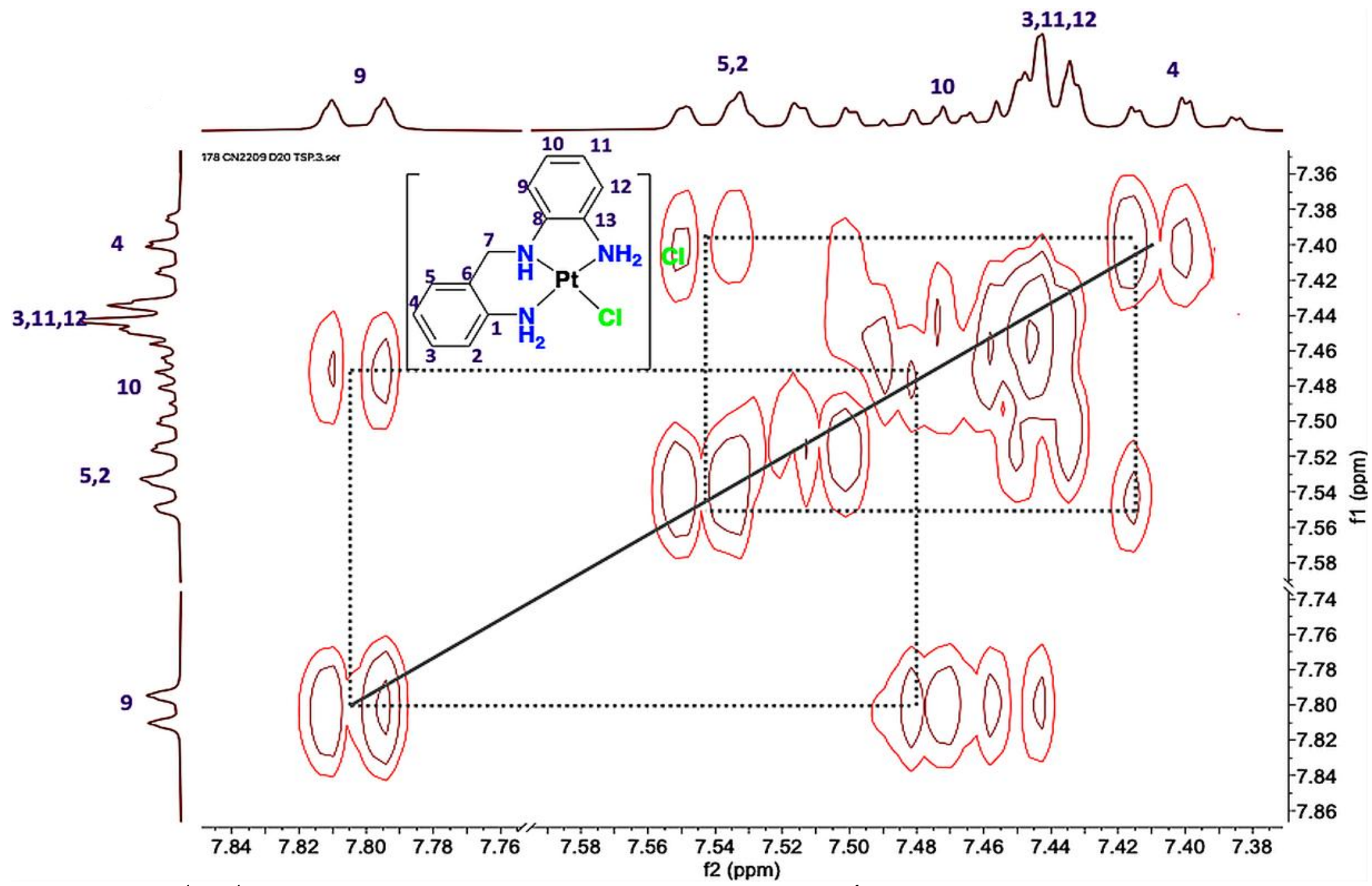

Figure SI 23. 2D [^{1}H, ^{1}H] COSY NMR spectrum of complex ***[PtCl(L^{1}H)]Cl****, at 25 °C in D_2O +TSP, showing only the signals corresponding to the observed correlations.*

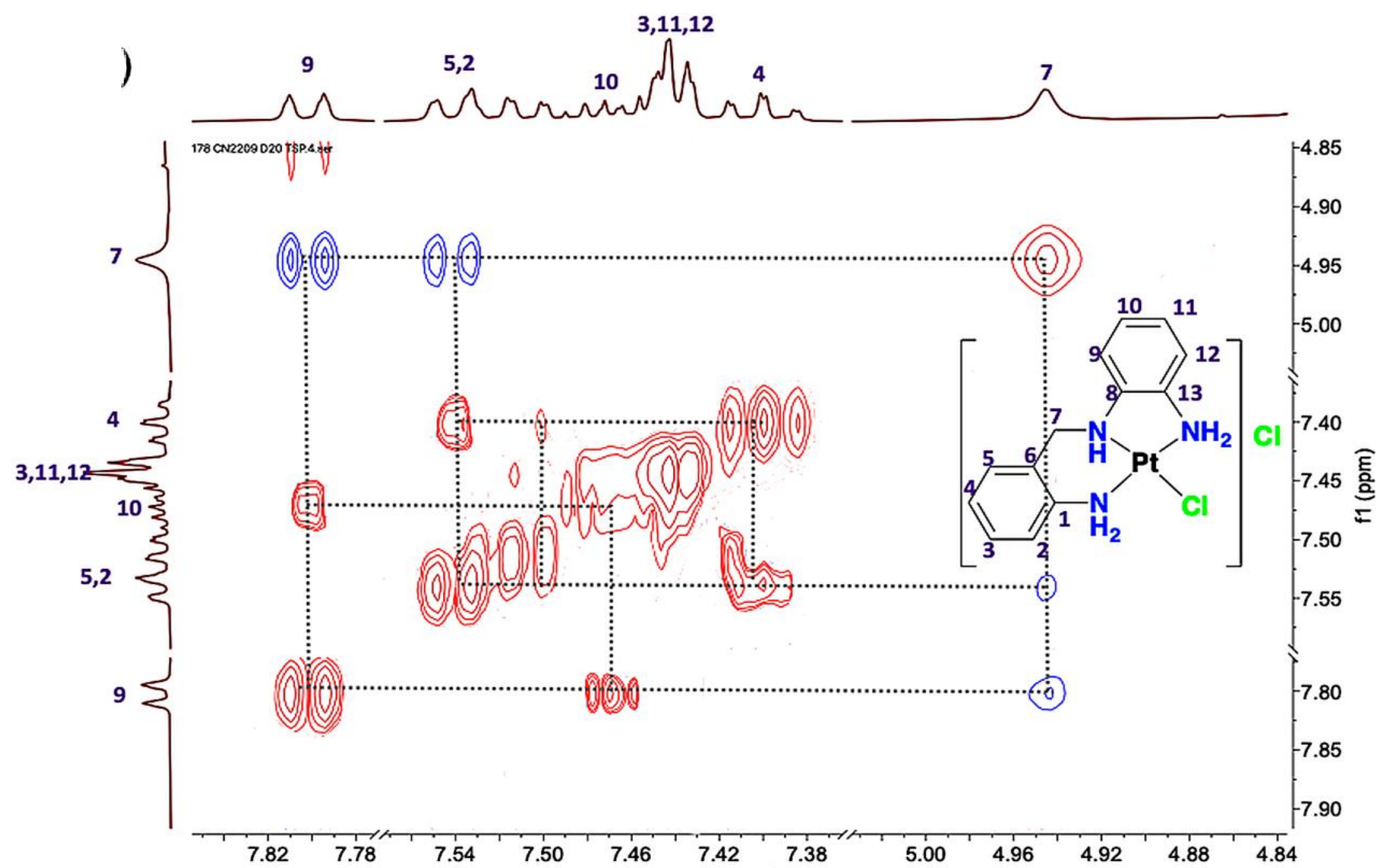

Figure SI 24. 2D [^{1}H, ^{1}H] NOESY NMR spectrum of complex ***[PtCl(L^{1}H)]Cl*** *at 25 °C in D_2O +TSP, showing only the signals corresponding to the observed correlations.*

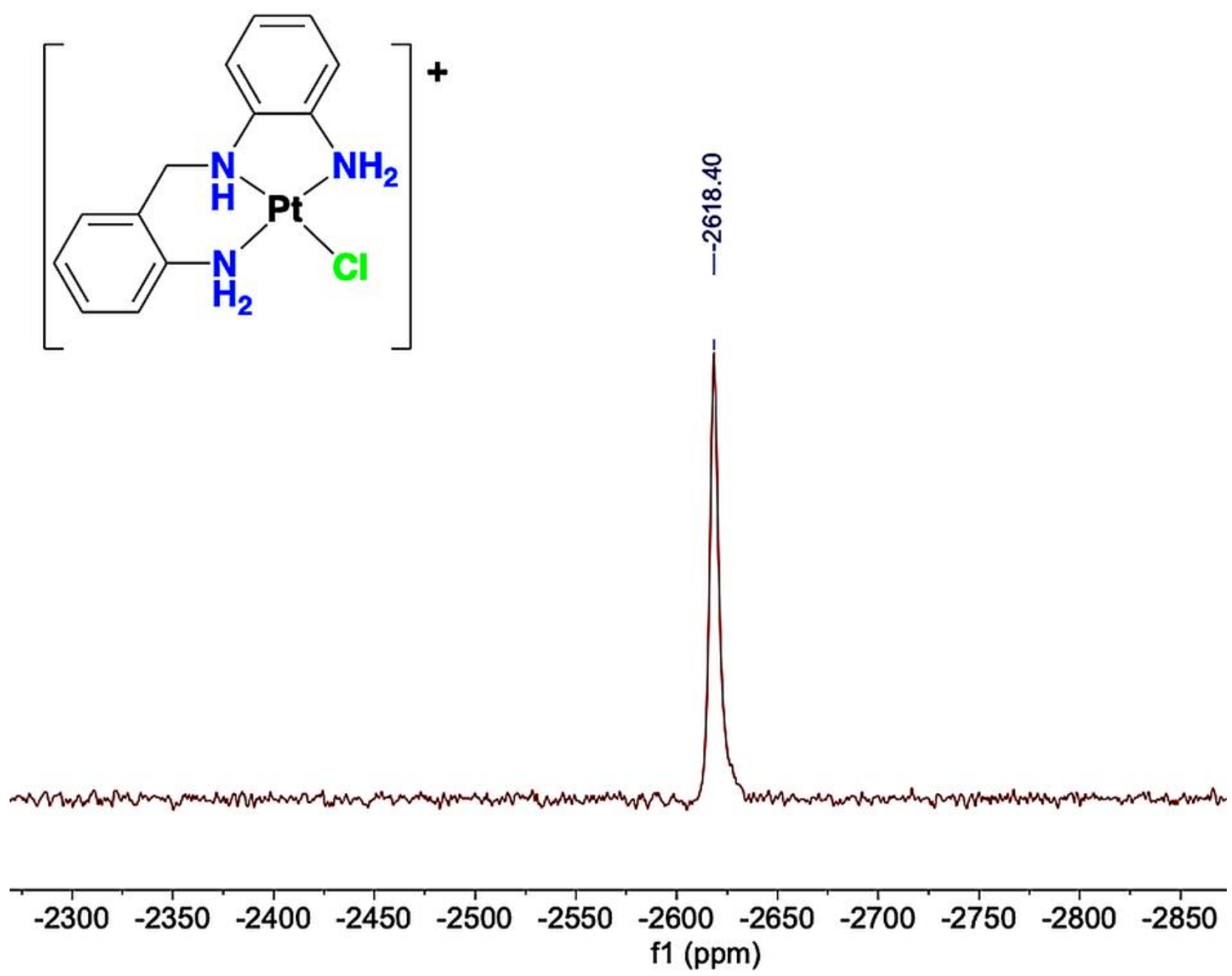

*Figure SI 25. ^{195}Pt NMR spectrum of complex **[PtCl(L^{1}H)]Cl** at 25 °C in D_2O.*

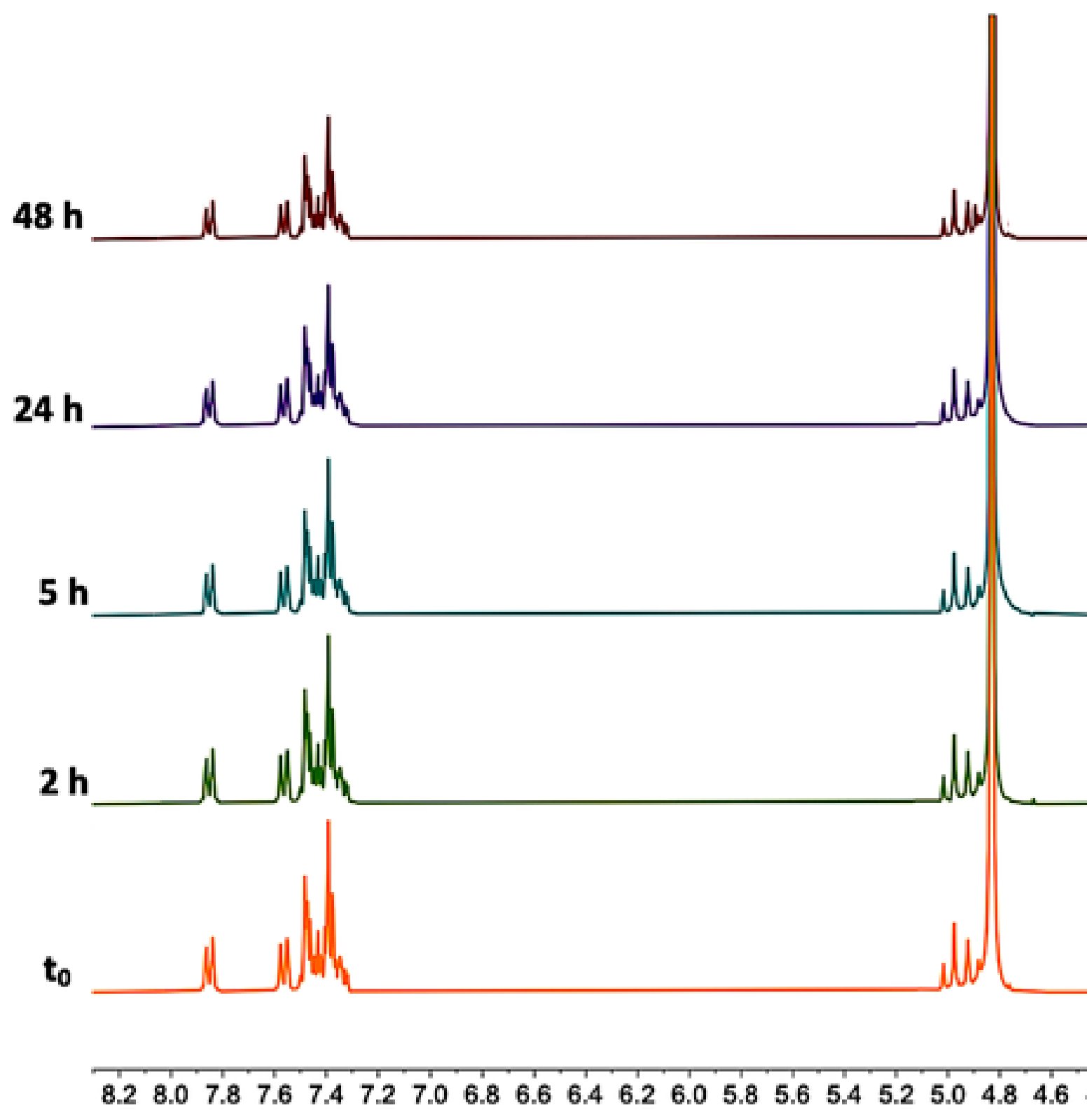

*Figure SI 26. ^{1}H NMR stability test spectrums of complex **[PtCl(L^{1}H)]Cl** at 25 °C in MeOH-d_4.*

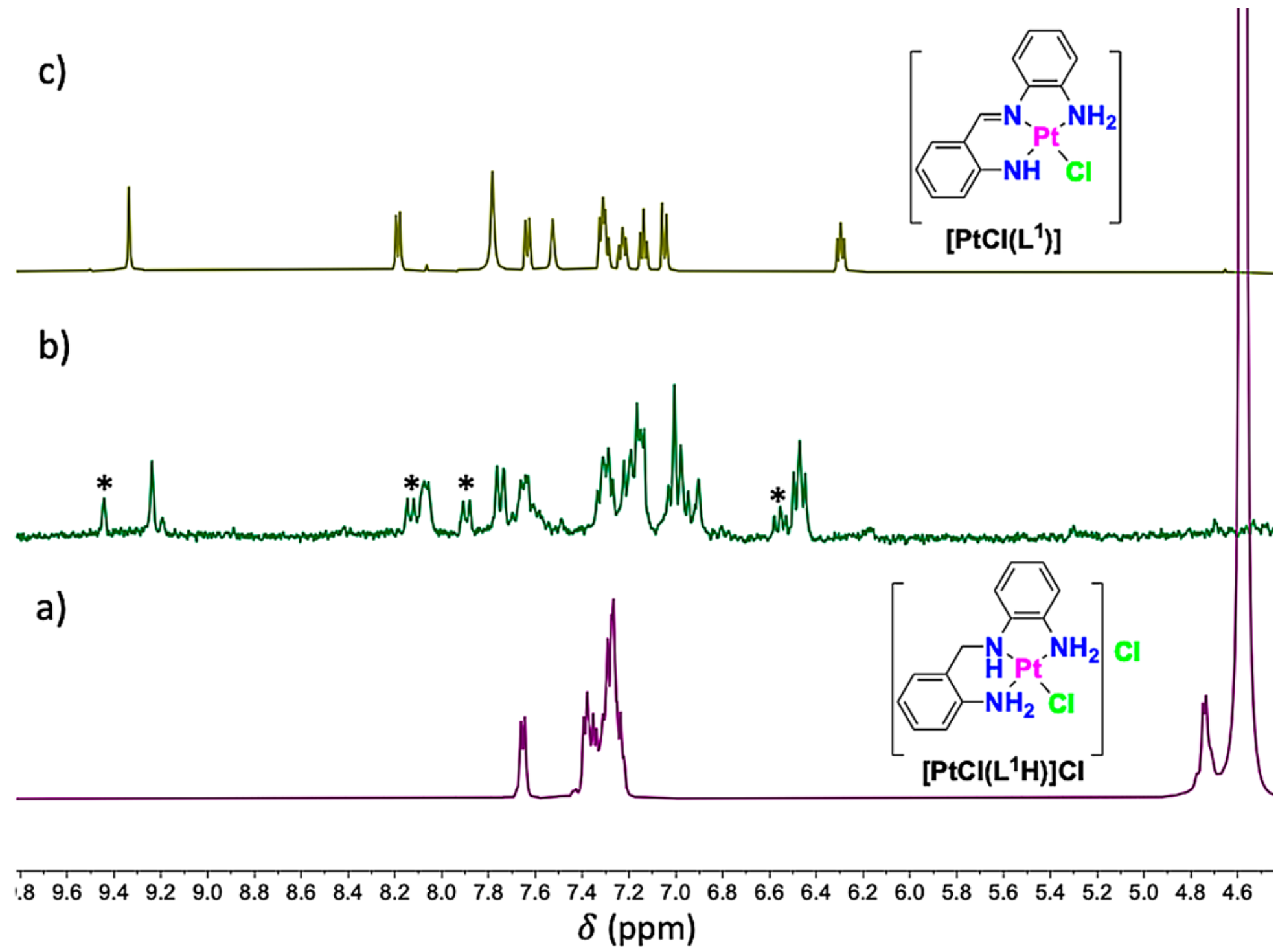

Figure SI 27. Monitoring of oxidative dehydrogenation reaction by ^{1}H NMR: a) initial spectrum of ***[PtCl(L¹H)]Cl,*** *b) spectrum of mixture of species of the oxidized ligand after 5 h under a stream of air and, c) spectrum of final product complex* ***[PtCl(L¹)]****, at 25 °C in DMSO-d_6.*

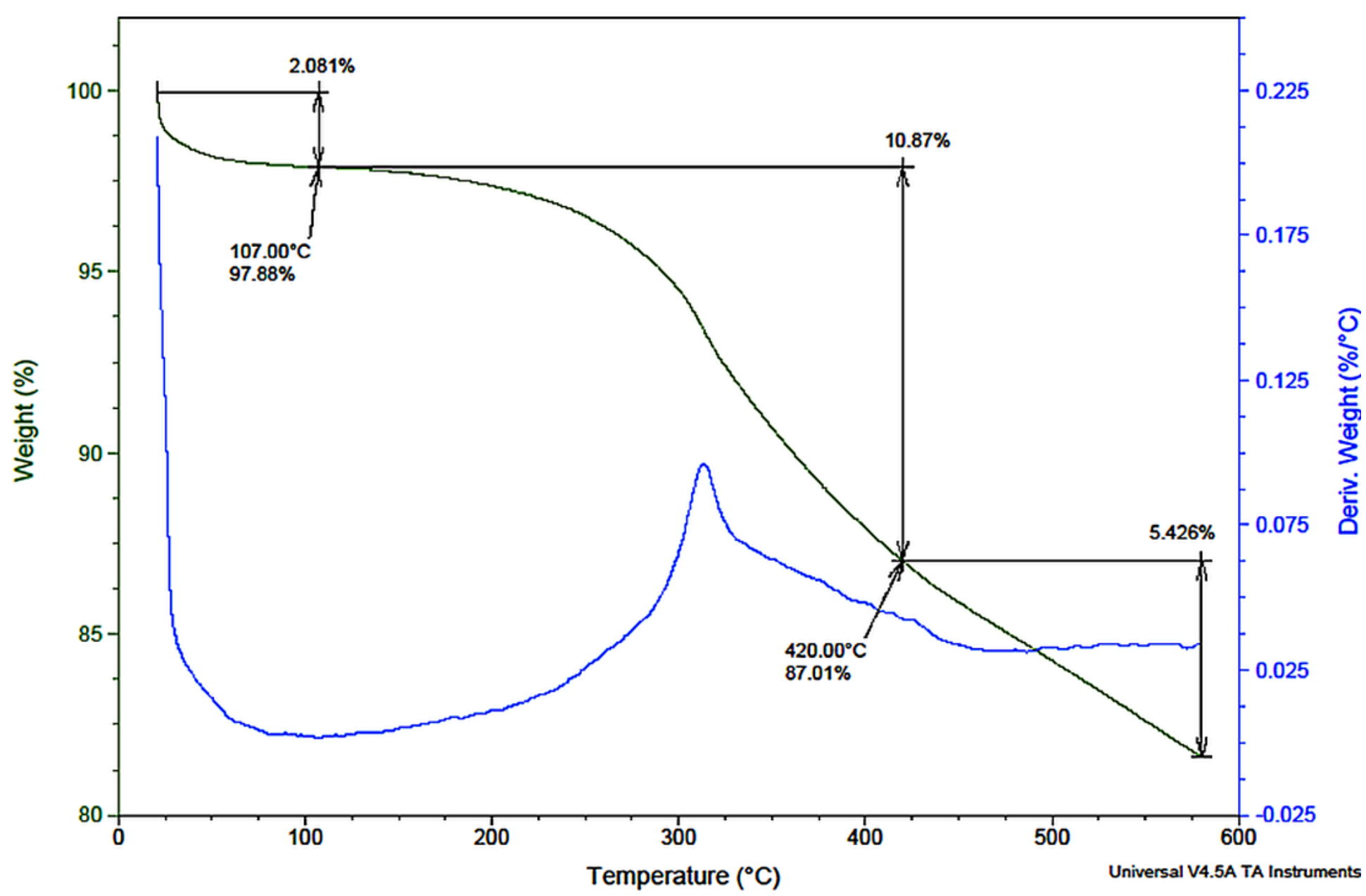

Figure SI 28. TGA of Complex ***[PtCl(L¹)].*** *The trace shows a small initial mass loss at low temperature (~20 °C, below of 17 °C), consistent with the release of adsorbed solvent, followed by a major decomposition step at higher temperature (centered at ~420 °C). Elemental analysis is best fitted by the formulation $[PtC_{13}H_{13}N_3Cl]\cdot(C_6H_{14})_{0.20}$ (calc for C, N, H: 37.15, 9.15, 3.47%), supporting the presence of a minor amount hexane of trapped in the isolated solid.*

Additional spectra for complex [PtCl(L[1])] characterization

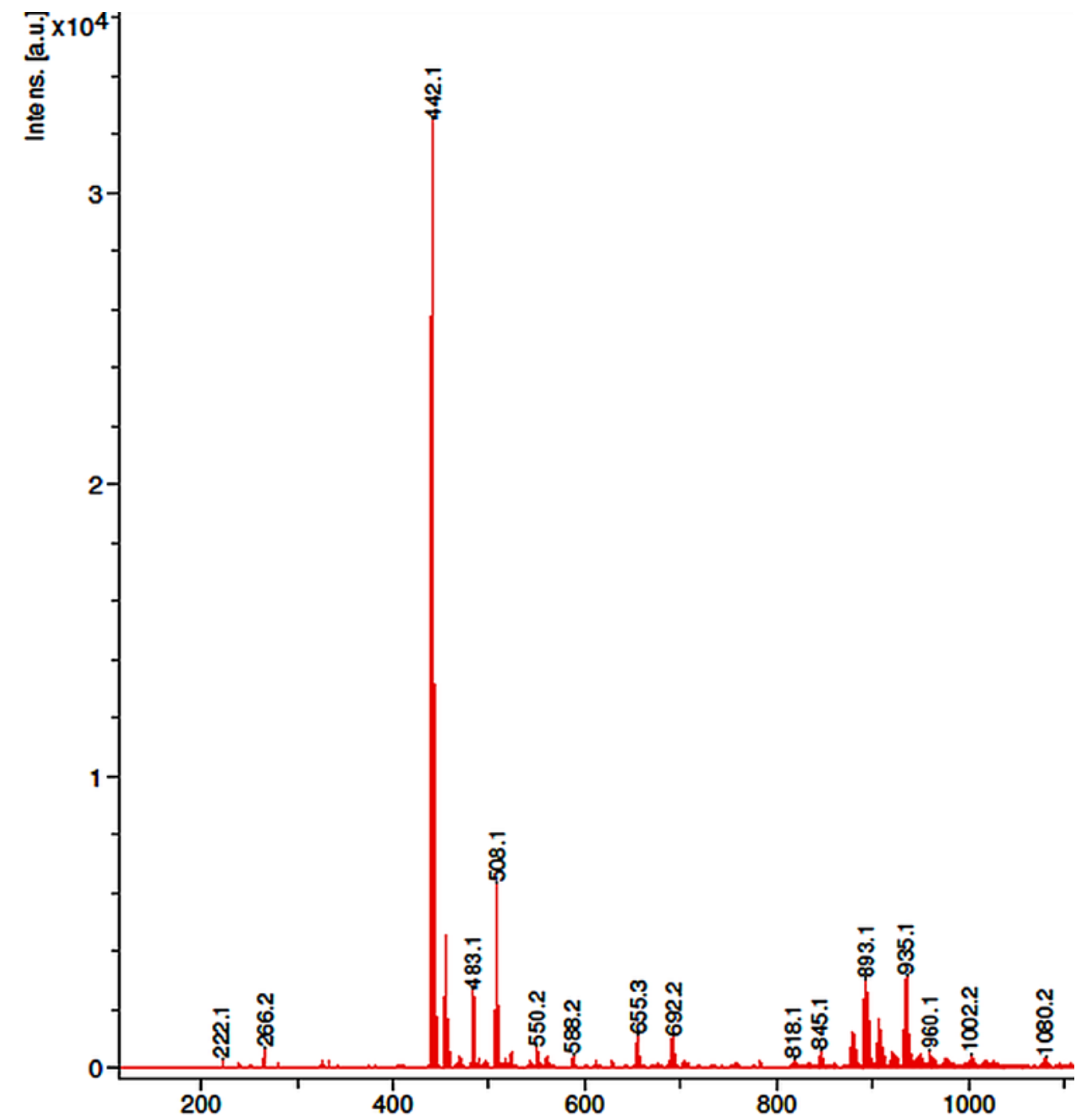

*Figure SI 29. MALDI-MS+ mass spectrum of Complex **[PtCl(L[1])]**.*

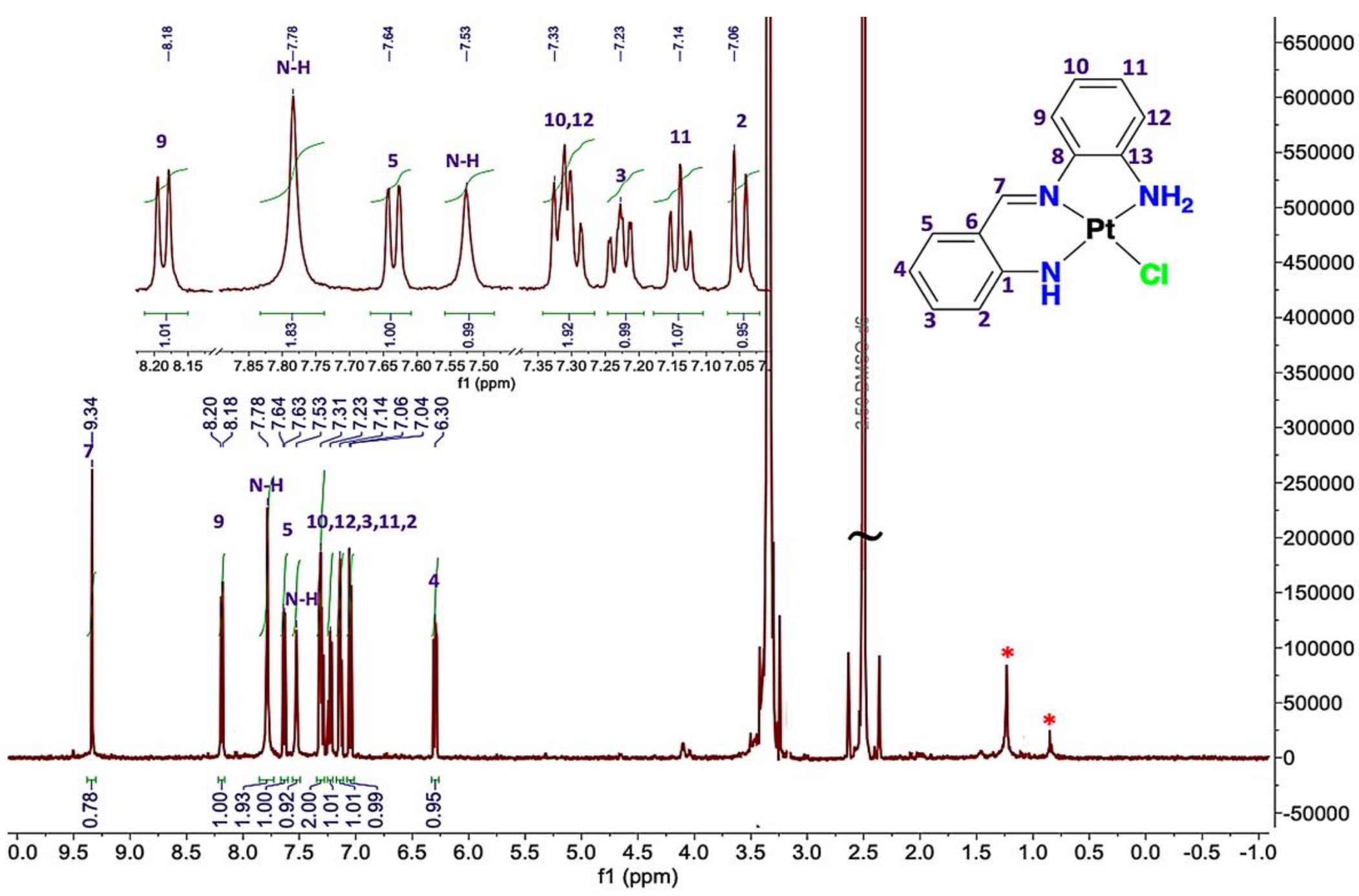

*Figure SI 30. ^{1}H NMR spectrum of complex **[PtCl(L[1])]** at 25 °C in DMSO-d6. *The spectrum displays the characteristic signals at δ 0.86 ppm and δ 1.25 ppm of n-hexane, confirms the incorporation of a minor amount of residual solvent in the isolated solid, in agreement with the elemental analysis and thermogravimetric data.*

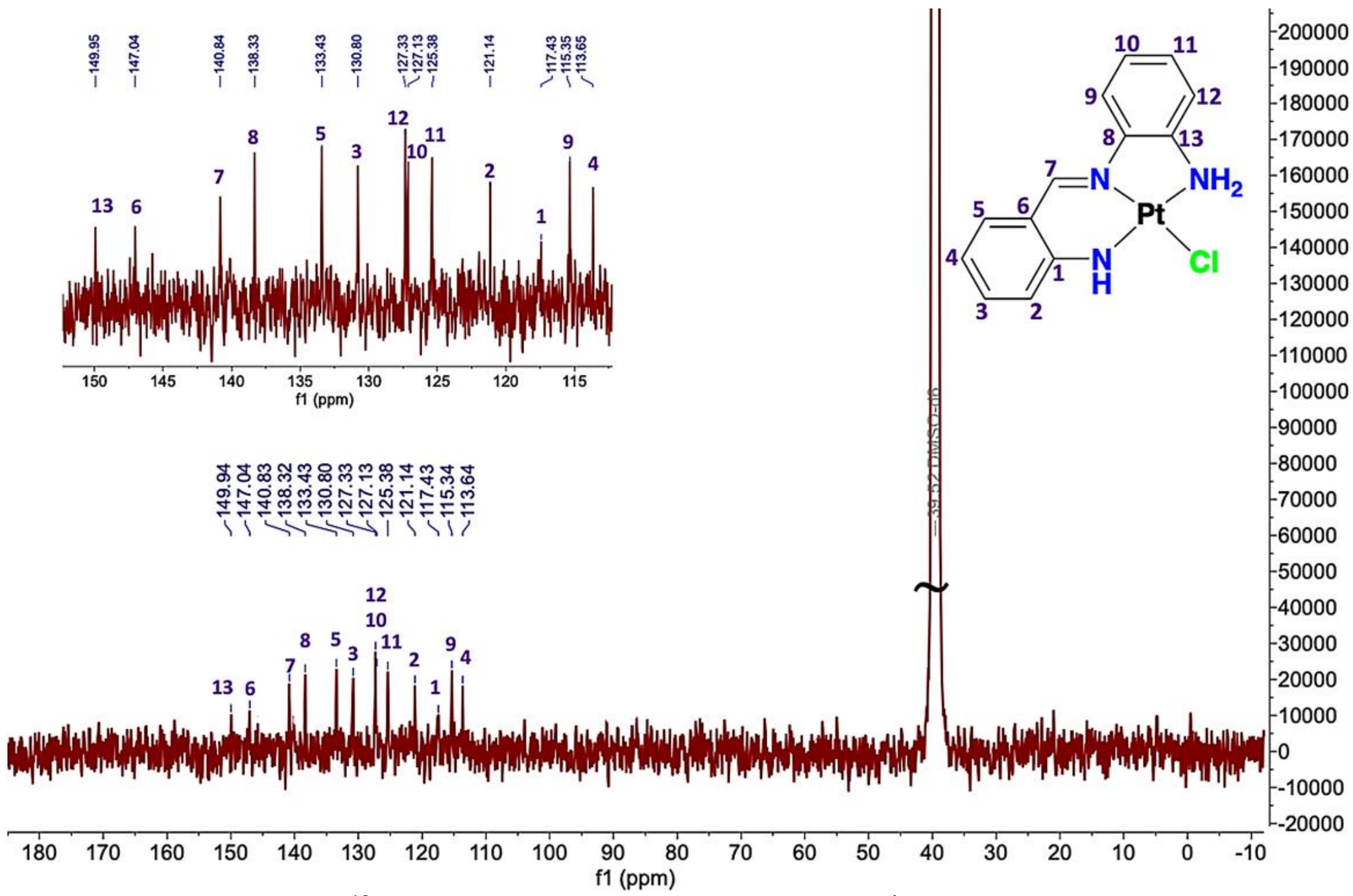

*Figure SI 31. ^{13}C NMR spectrum of Complex **[PtCl(L[1])]** at 25 °C in DMSO-d6.*

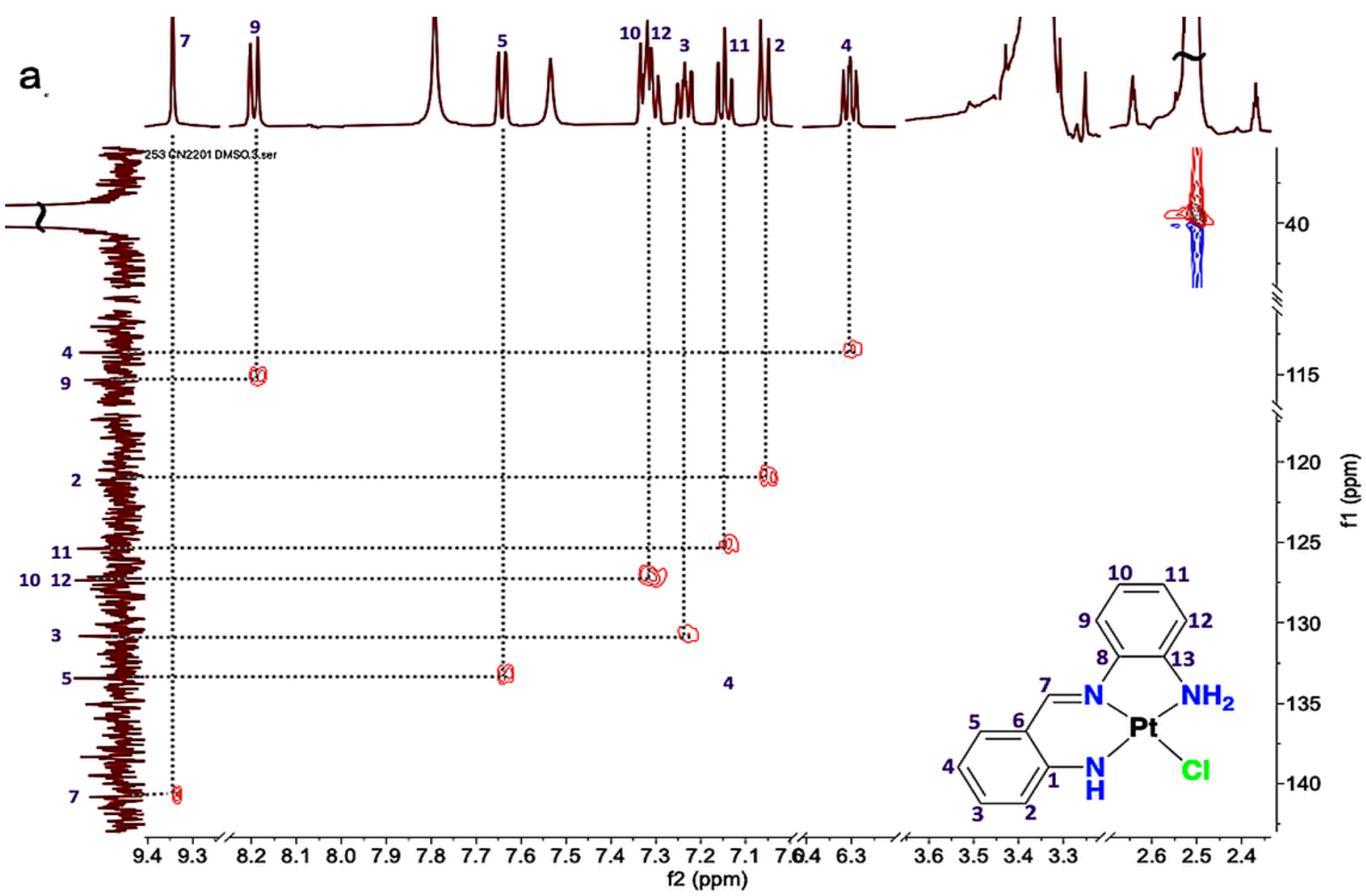

*Figure SI 32. 2D [^{1}H, ^{13}C] HSQC NMR spectrum of complex **[PtCl(L[1])]** at 25 °C in DMSO-d6, showing only the signals corresponding to the observed correlations.*

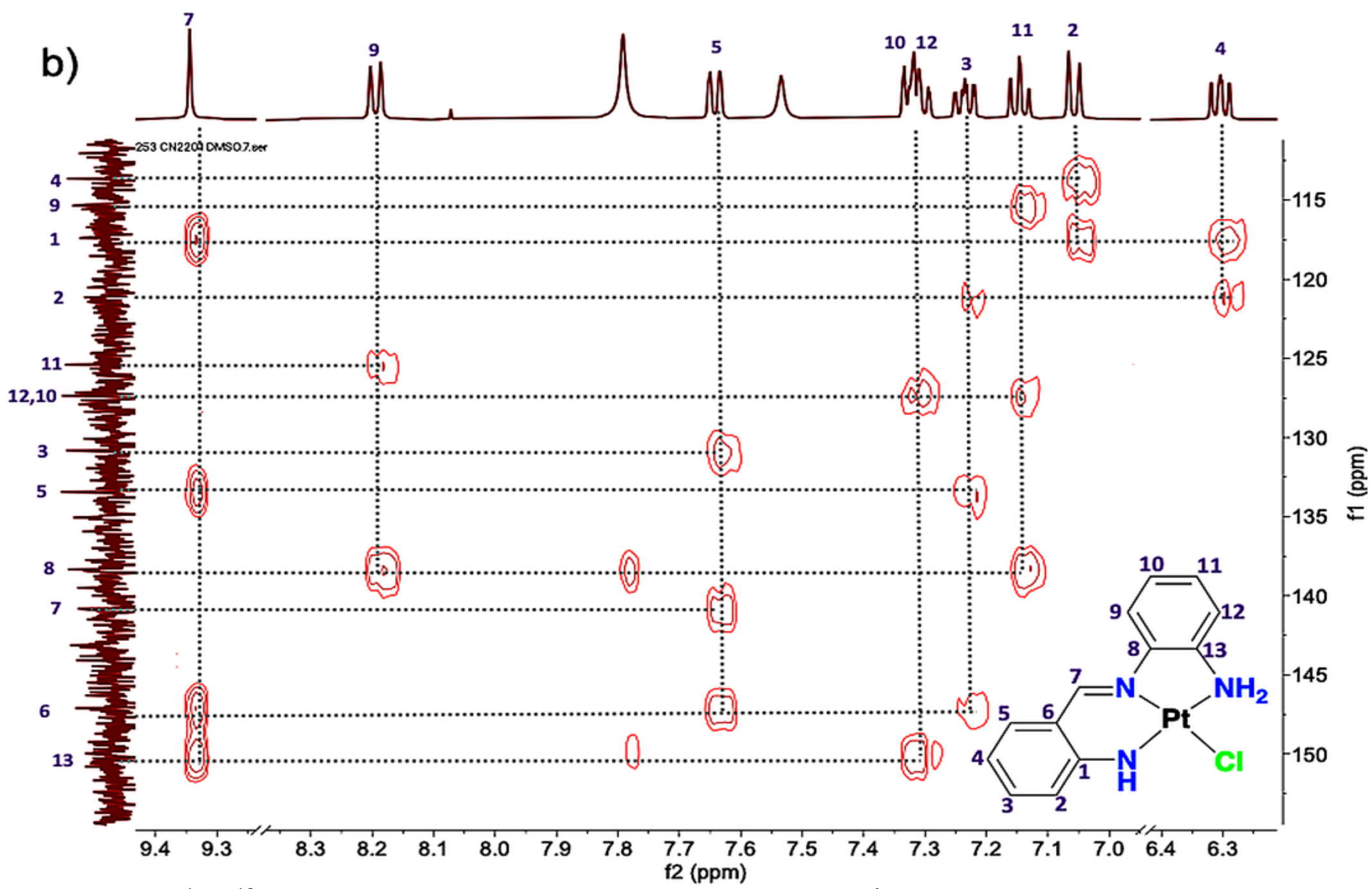

*Figure SI 33. 2D [^{1}H, ^{13}C] HMBC NMR spectrumof complex **[PtCl(L^1)]** at 25 °C in DMSO-d$_6$, showing only the signals corresponding to the observed correlations.*

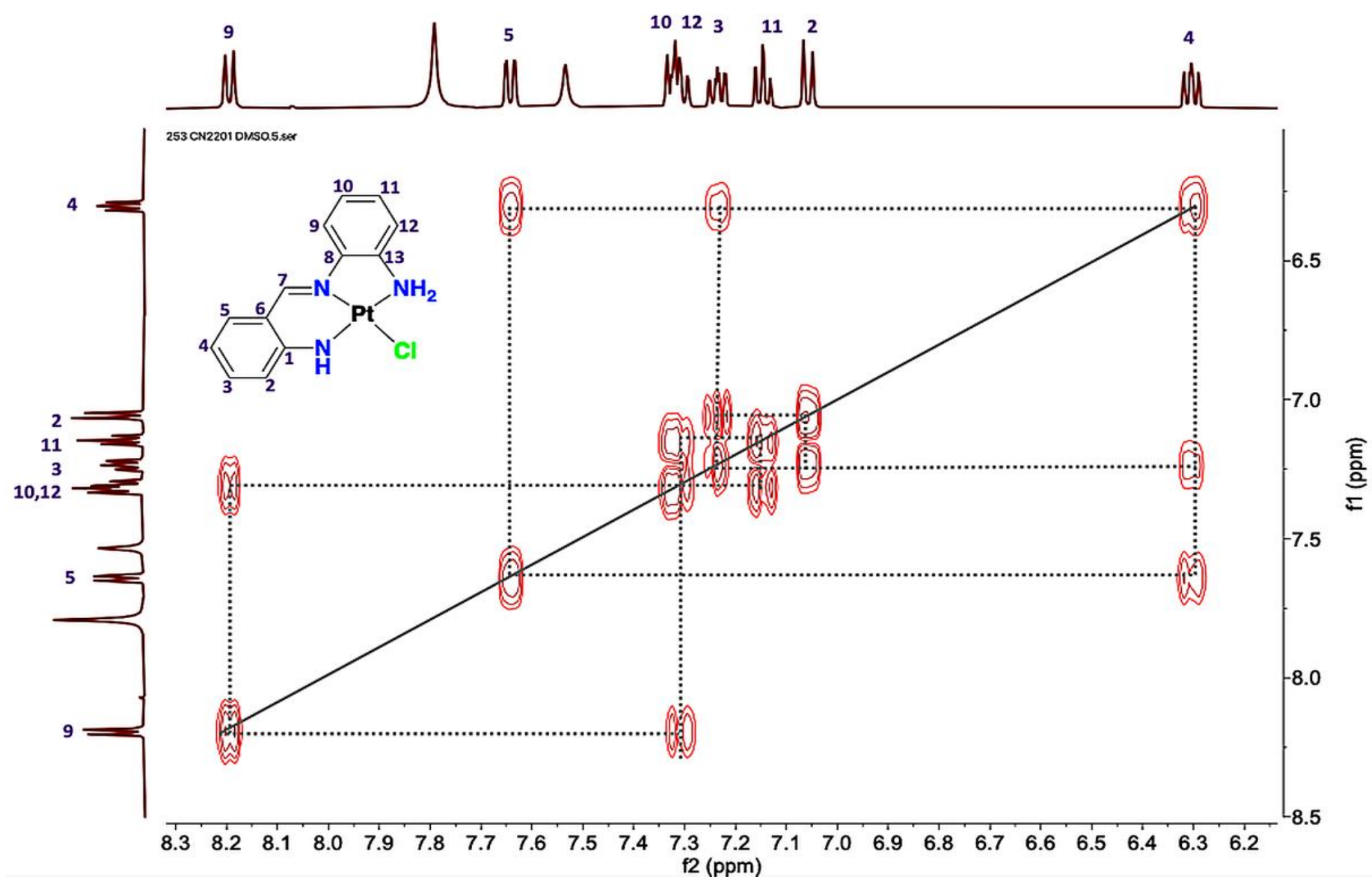

*Figure SI 34. 2D [^{1}H, ^{1}H] COSY NMR spectrum of complex **[PtCl(L^1)]** at 25 °C in DMSO-d$_6$, showing only the signals corresponding to the observed correlations.*

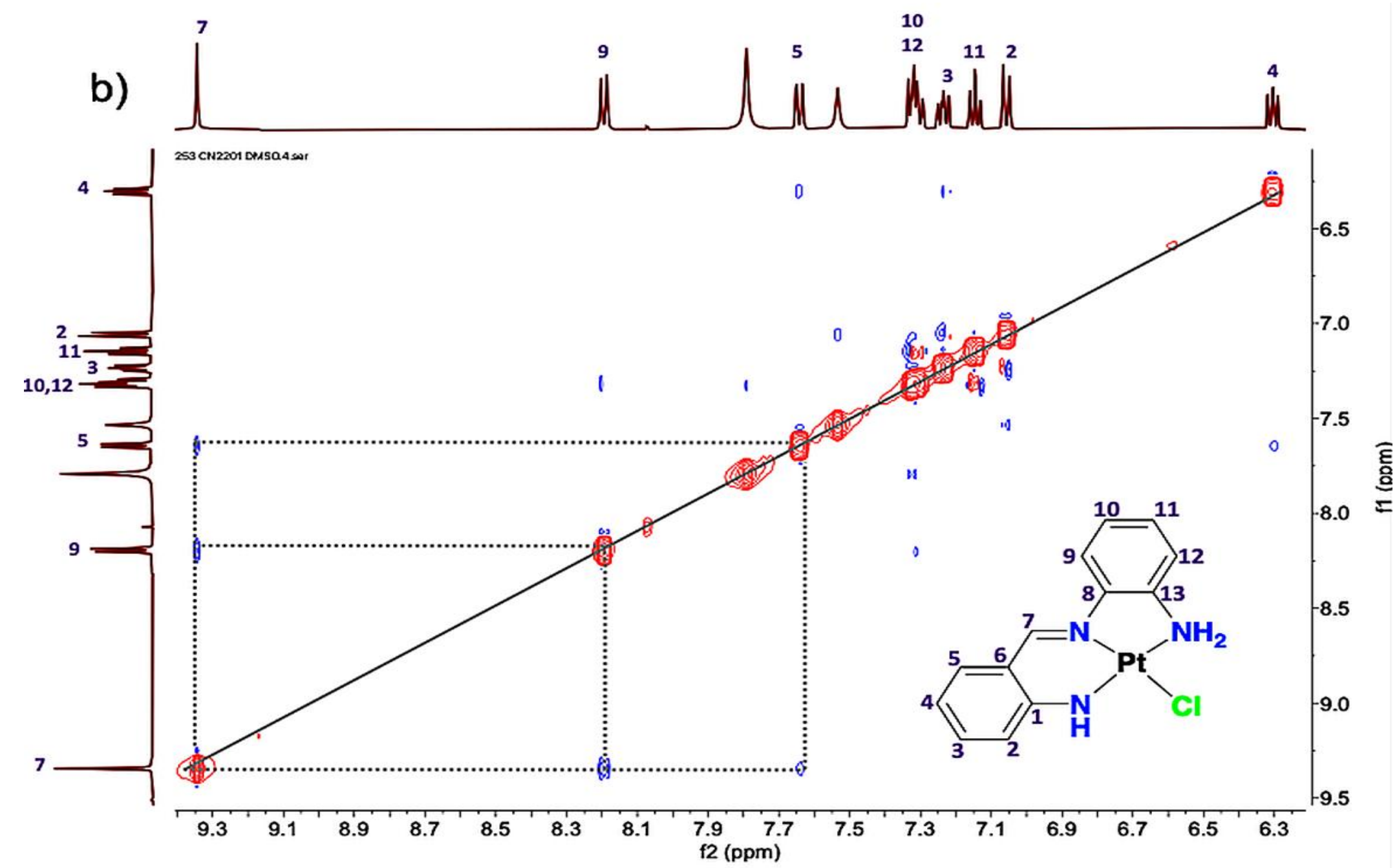

*Figure SI 35. 2D [^{1}H, ^{1}H] NOESY NMR spectrum of complex **[PtCl(L^1)]** at 25 °C in DMSO-d$_6$, showing only the signals corresponding to the observed correlations.*

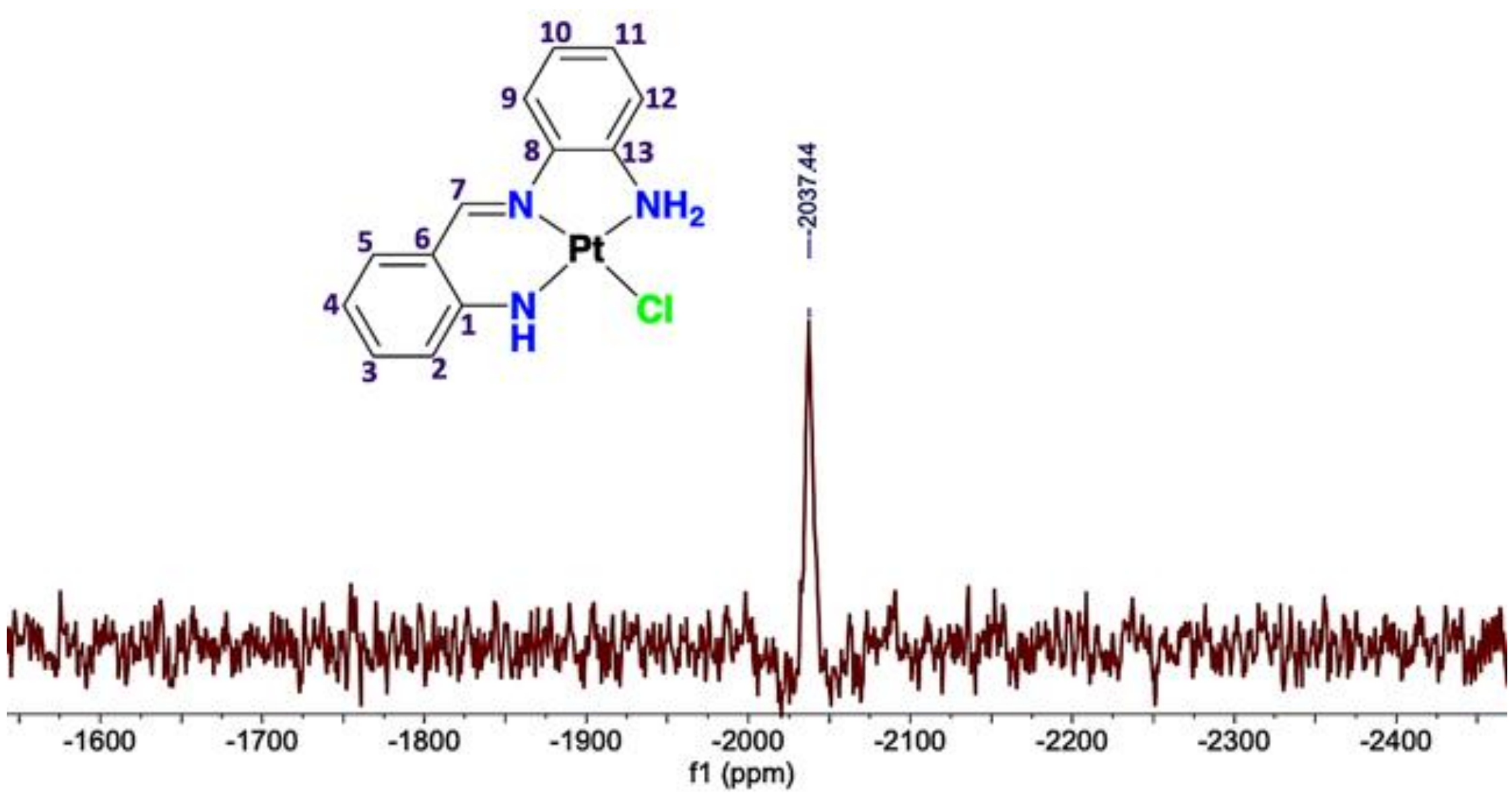

*Figure SI 36. ^{195}Pt NMR spectrum of complex **[PtCl(L^1)]** at 25 °C in DMSO-d$_6$.*

UV-Vis spectra for the stability studies of L^1H and L^1.

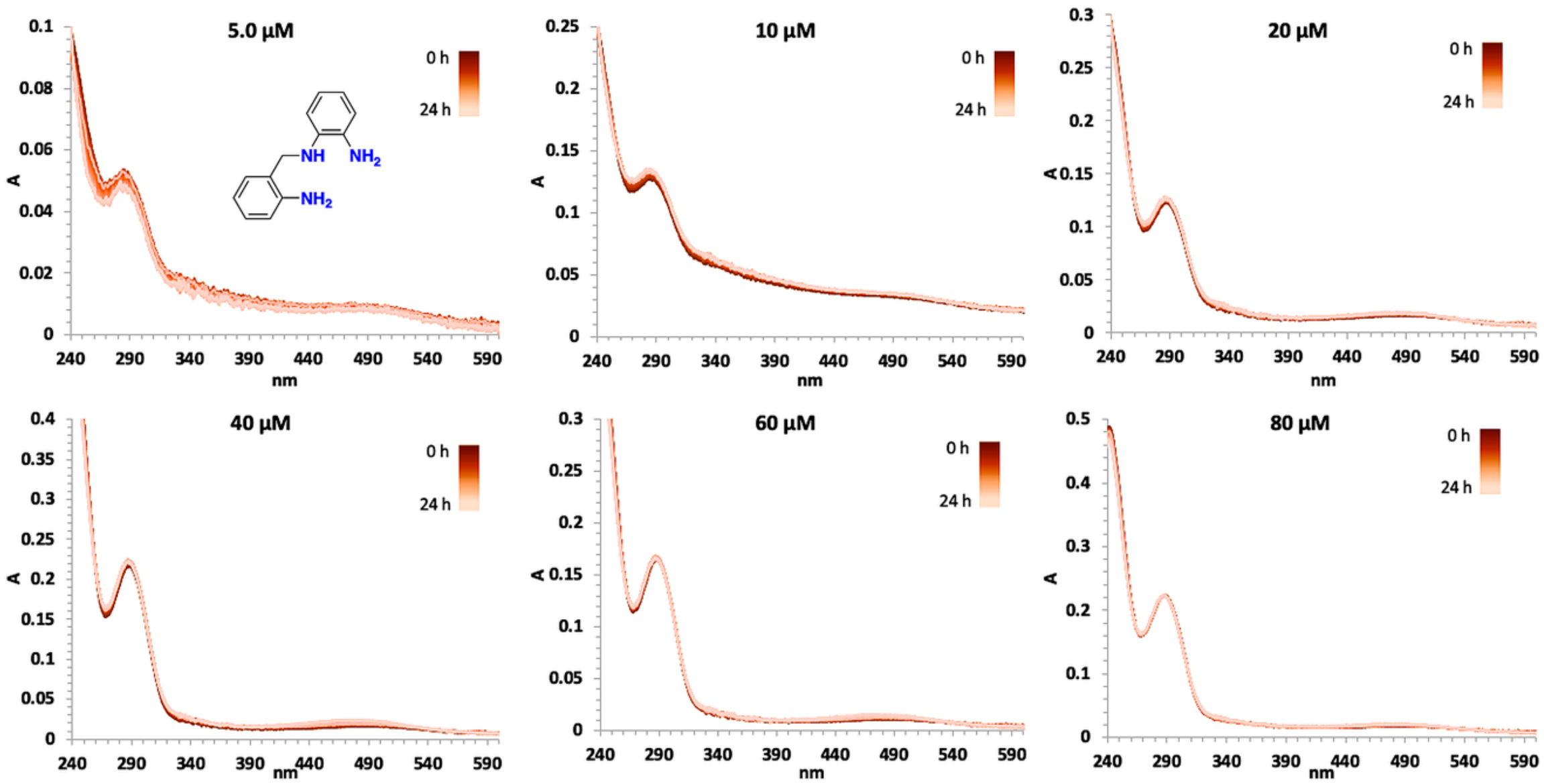

Figure SI 37. UV-Vis stability test of ***L^1H*** *in water 1% (v/v) DMSO and 37 °C for 24 h, at different concentrations.*

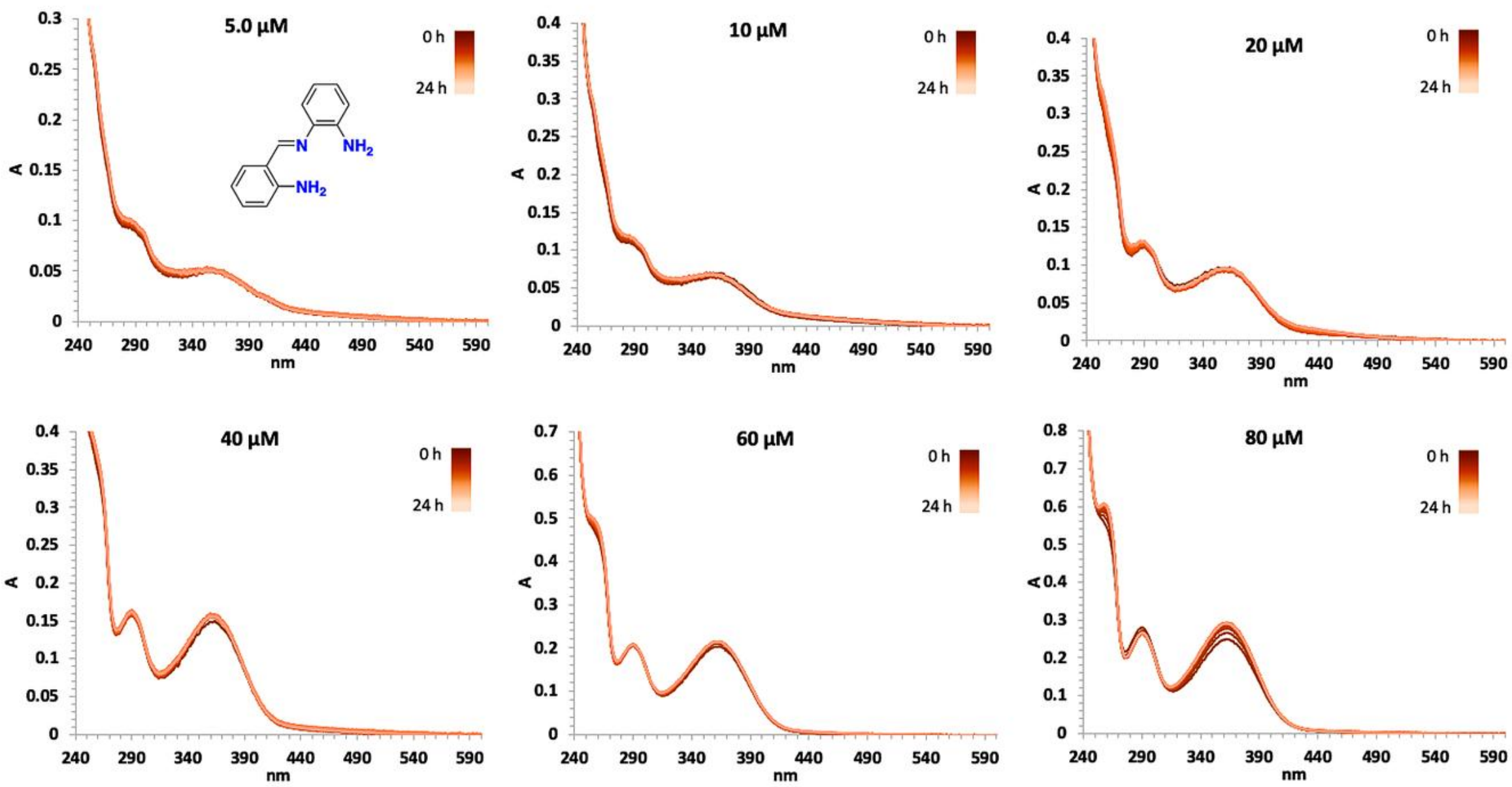

Figure SI 38. UV-Vis stability test of the ***L^1*** *in water 1 % (v/v) DMSO and 37 °C for 24 h, at different concentrations.*

UV-Vis spectra for the stability studies of PtCl(L[1]H)]Cl and [PtCl(L[1])]

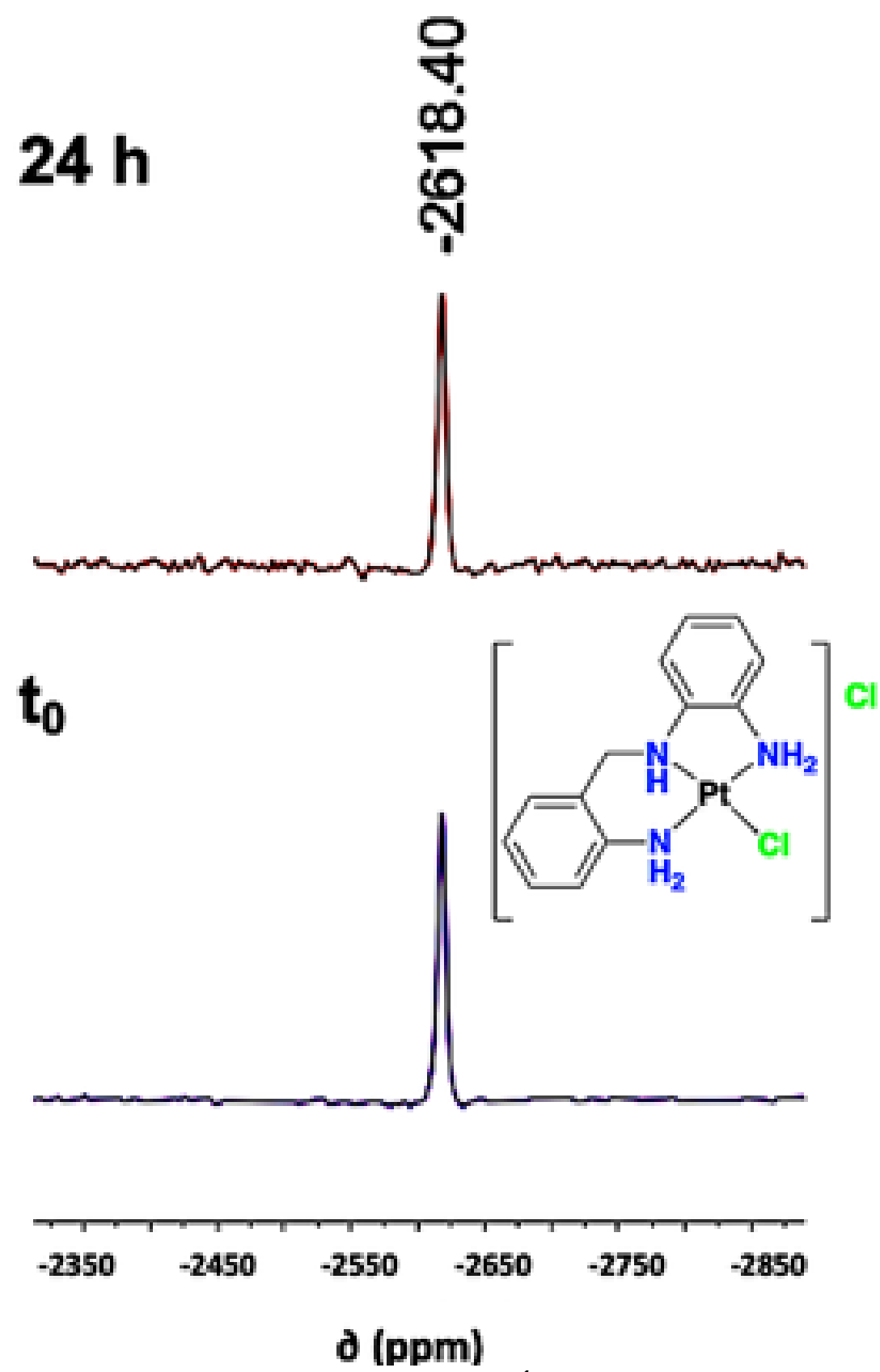

Figure SI 39. ^{195}Pt NMR stability test of ***[PtCl(L^{1}H)]Cl*** at 25 °C at t_0 and 24 h in D_2O.

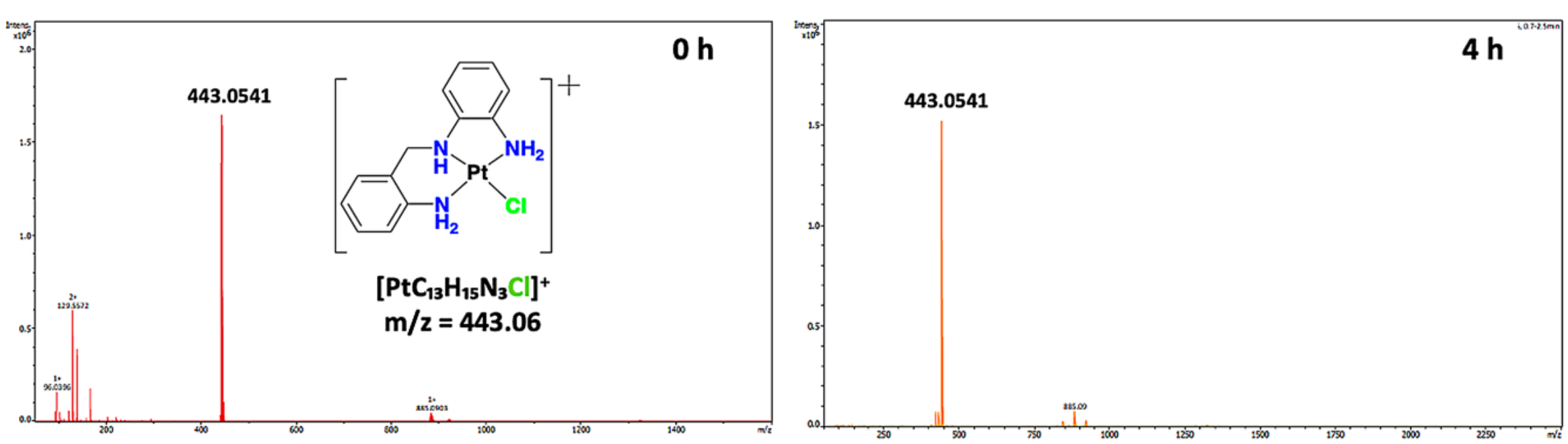

Figure SI 40. ESI-MS+ stability test of ***[PtCl(L^{1}H)]Cl*** at t_0 and 4 hours in water.

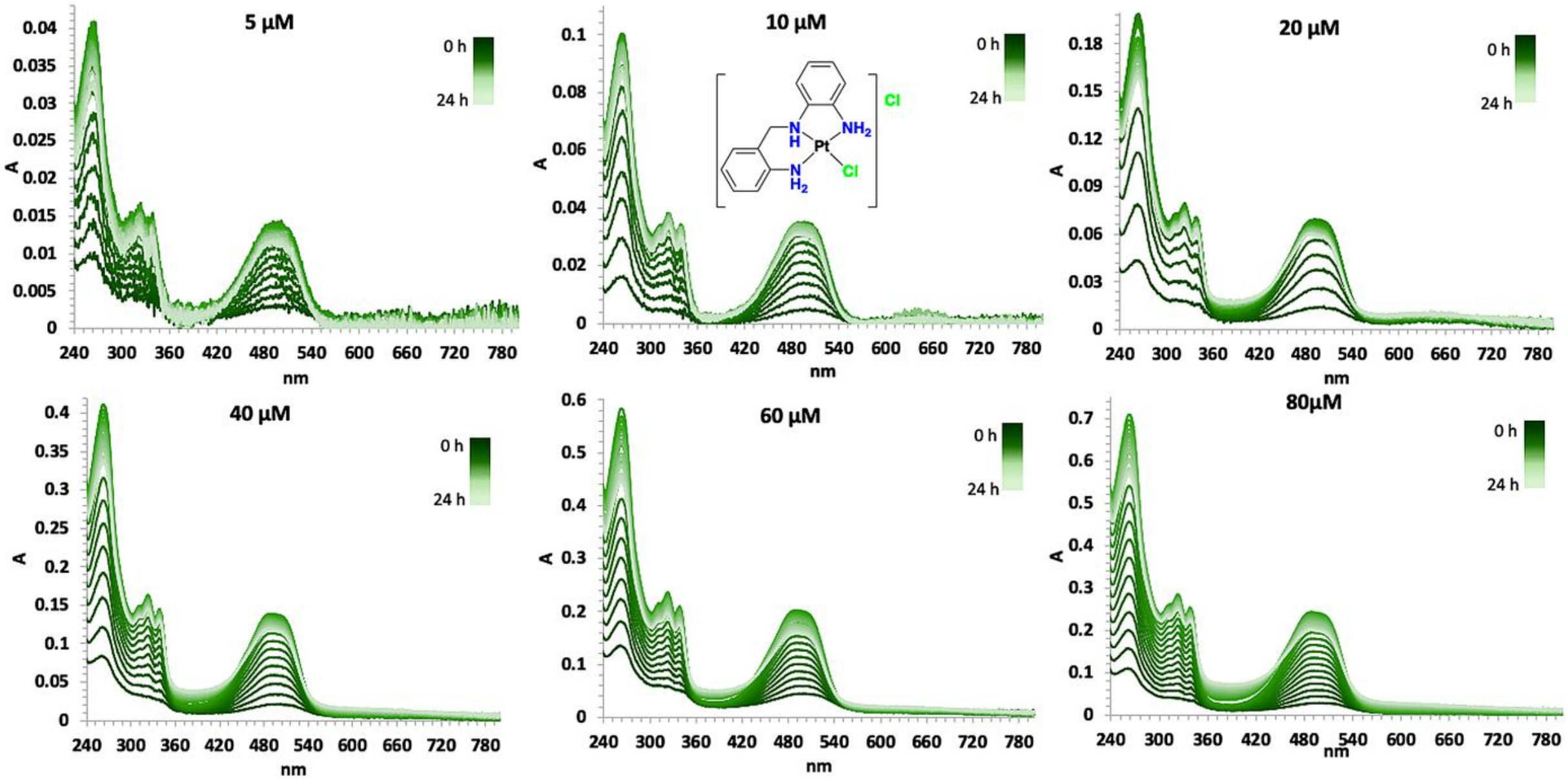

*Figure SI 41. UV-Vis stability test of complex **[PtCl(L1H)]Cl** at 37 °C in water for 24 h at different concentrations.*

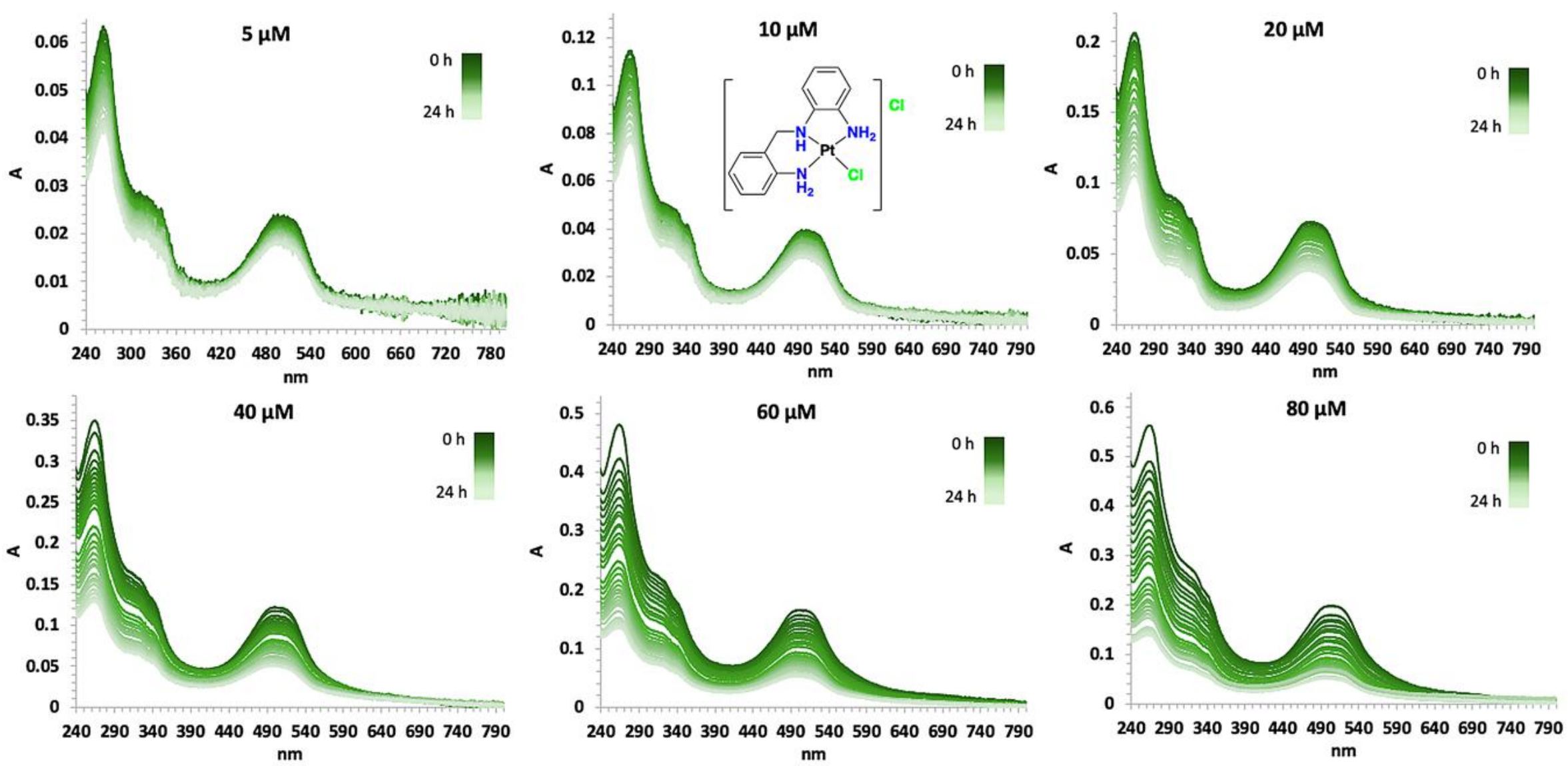

*Figure SI 42. UV-Vis stability of complex **[PtCl(L1H)]Cl** at 37 °C in Tris-HCl 5 mM pH = 7.4 for 24 h at different concentrations.*

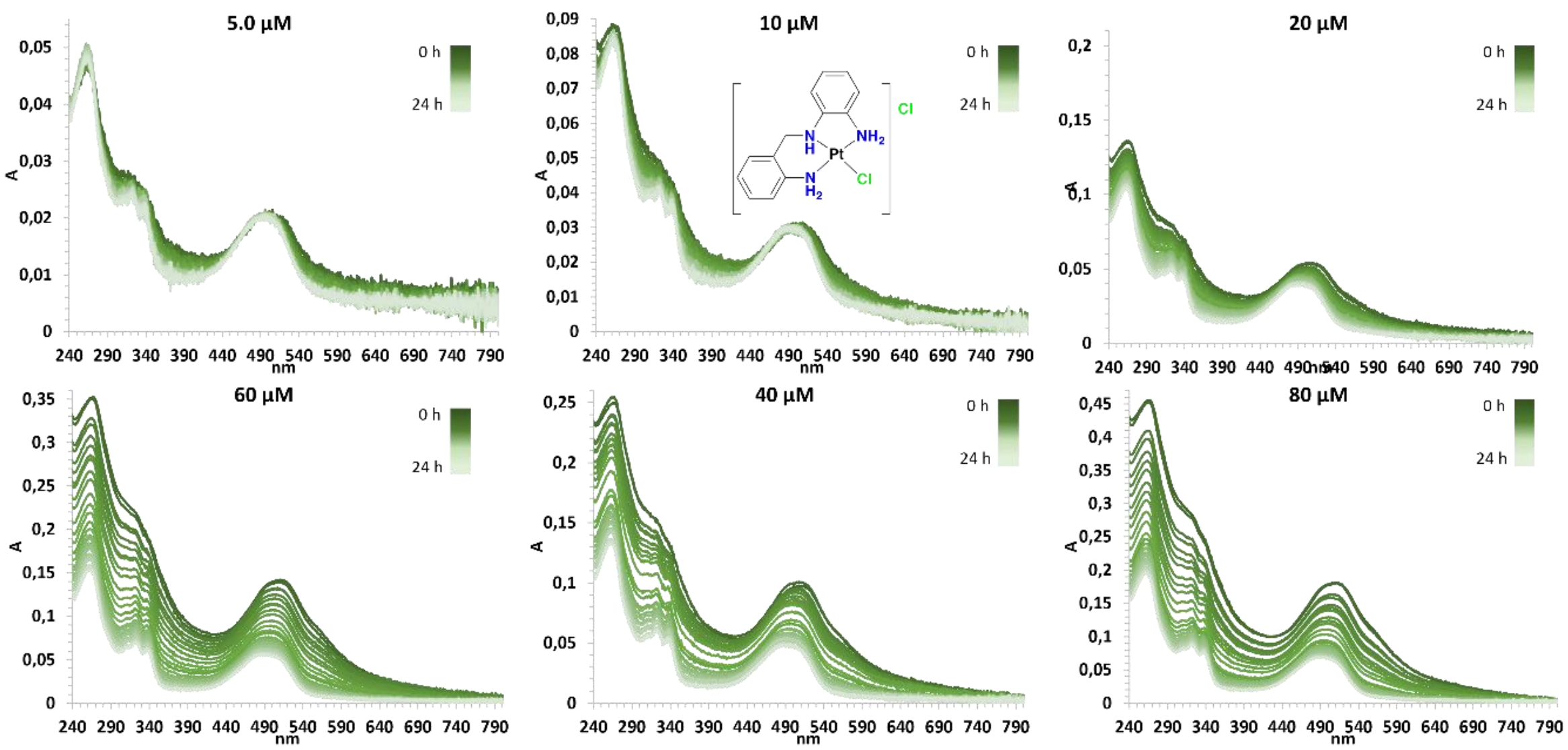

*Figure SI 43. UV-Vis stability of complex **[PtCl(L¹H)]Cl** at 37 °C in water $NaClO_4$ 5 mM for 24 h at different concentrations.*

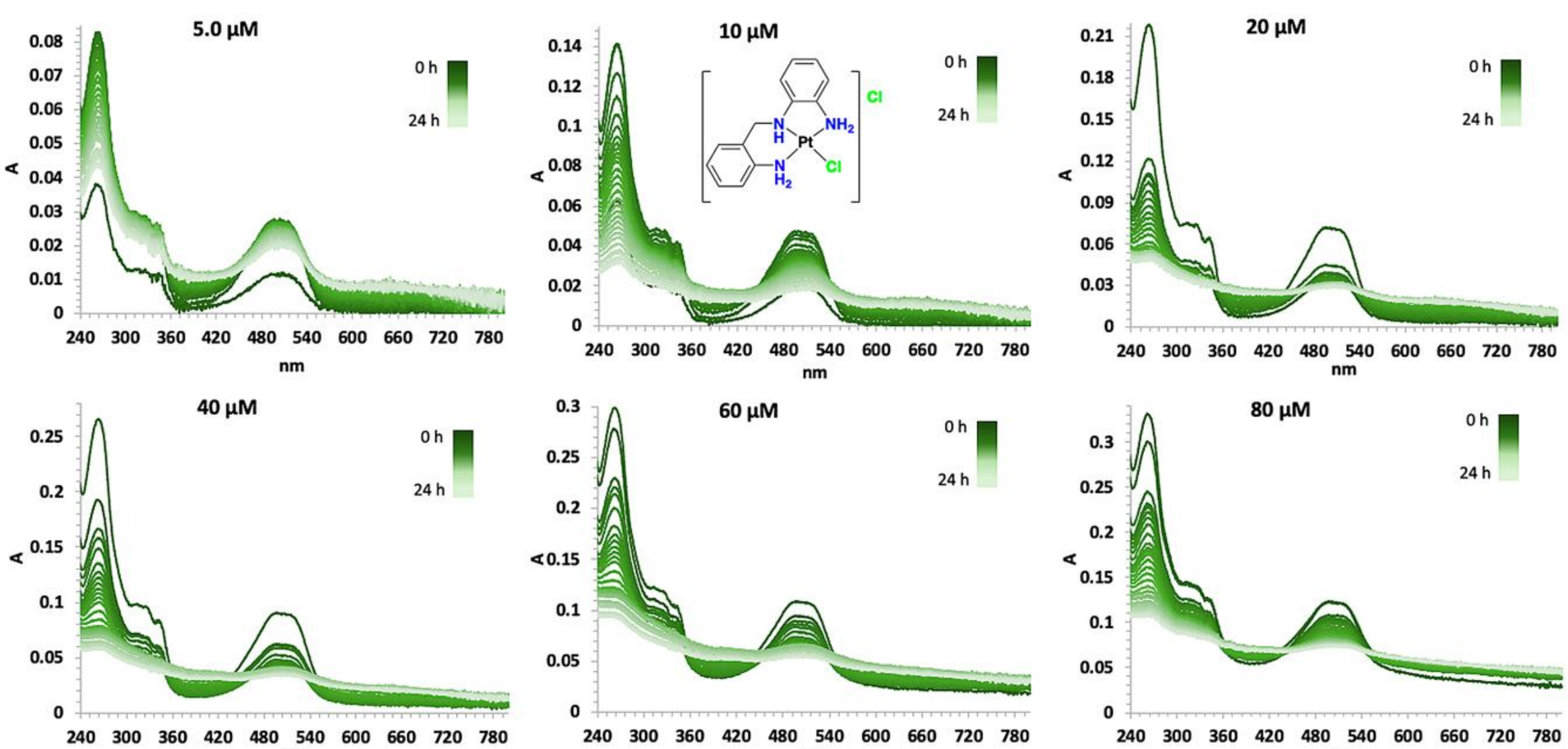

*Figure SI 44. UV-Vis stability of complex **[PtCl(L¹H)]Cl** at 37 °C in water 0.9% (w/v)NaCl for 24 h at different concentrations.*

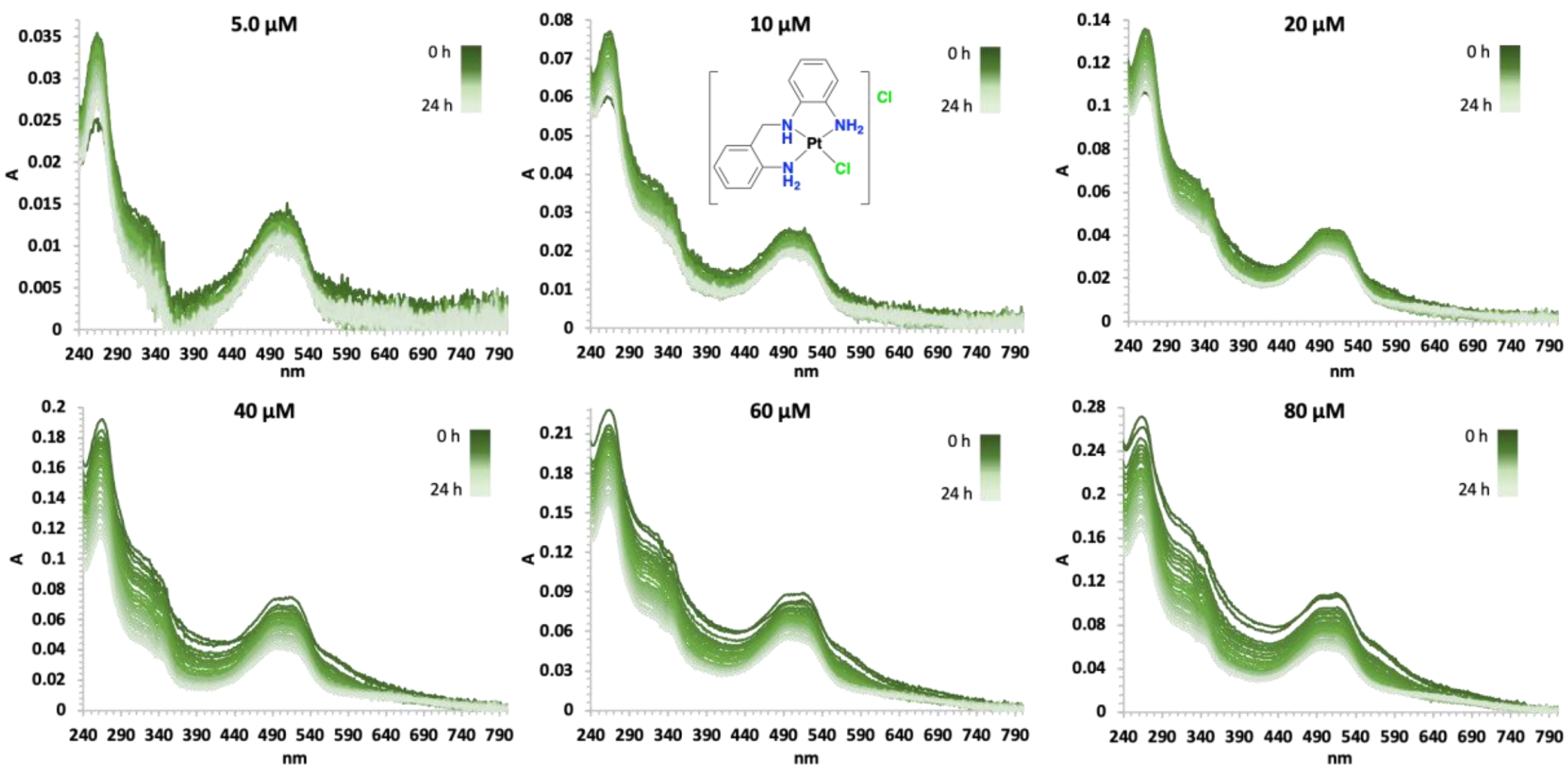

Figure SI 45. UV-Vis stability of complex ***[PtCl(L¹H)]Cl*** *at 37 °C in buffer phosphate 20 mM, pH 7.4, for 24 h at different concentrations.*

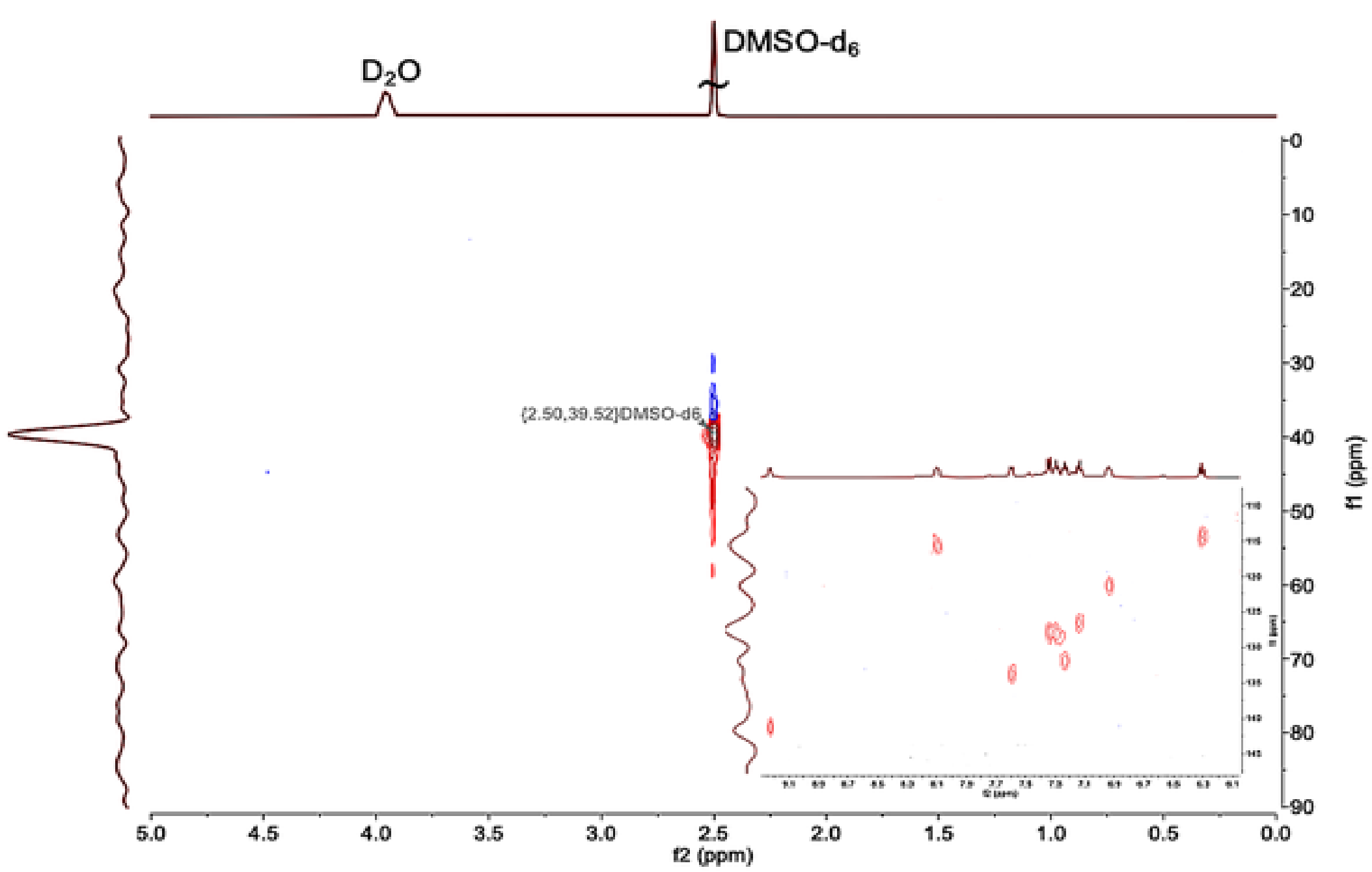

Figure SI 46. 2D NMR HSQC of complex ***[PtCl(L¹)]*** *at 25 °C after 24 hours in DMSO-d₆ + D₂O solution.*

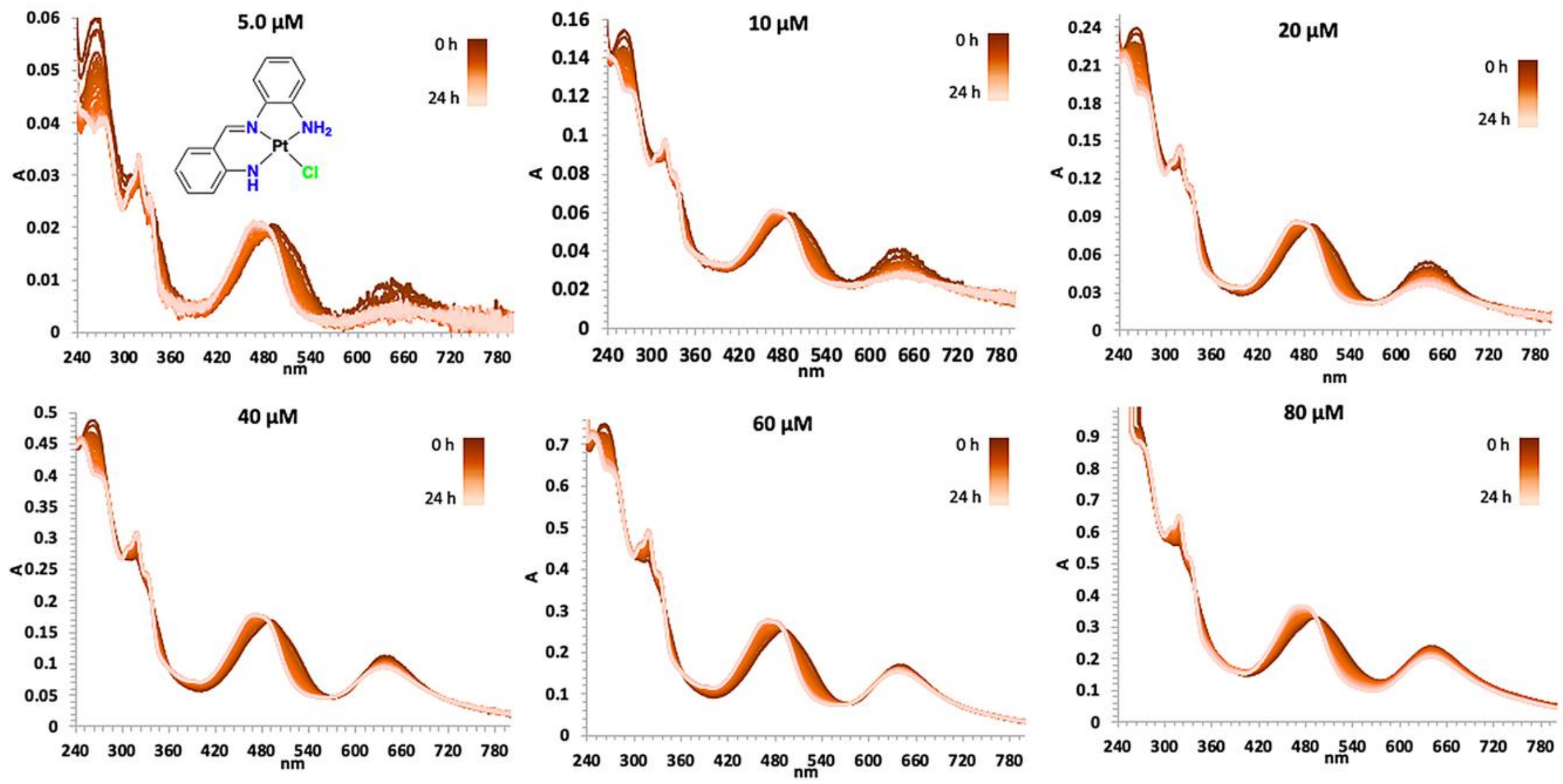

*Figure SI 47. UV-Vis stability of complex **[PtCl(L1)]** at 37 °C in water 1% (v/v) DMSO for 24 h at different concentrations.*

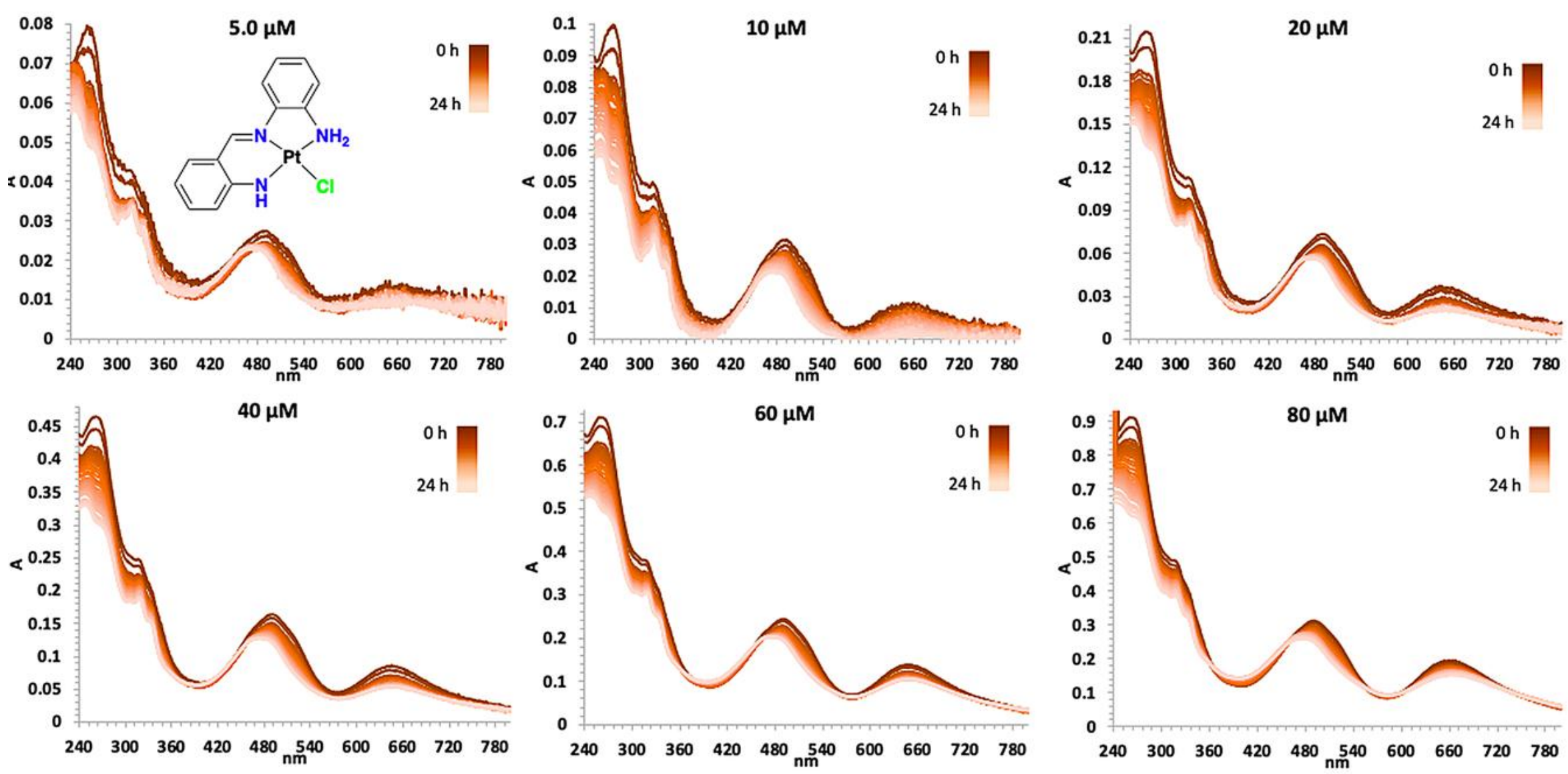

*Figure SI 48. UV-Vis stability of complex **[PtCl(L1)]** at 37 °C in Tris-HCl 5 mM, pH= 7.4 at 1% (v/v) DMSO for 24 h at different concentrations.*

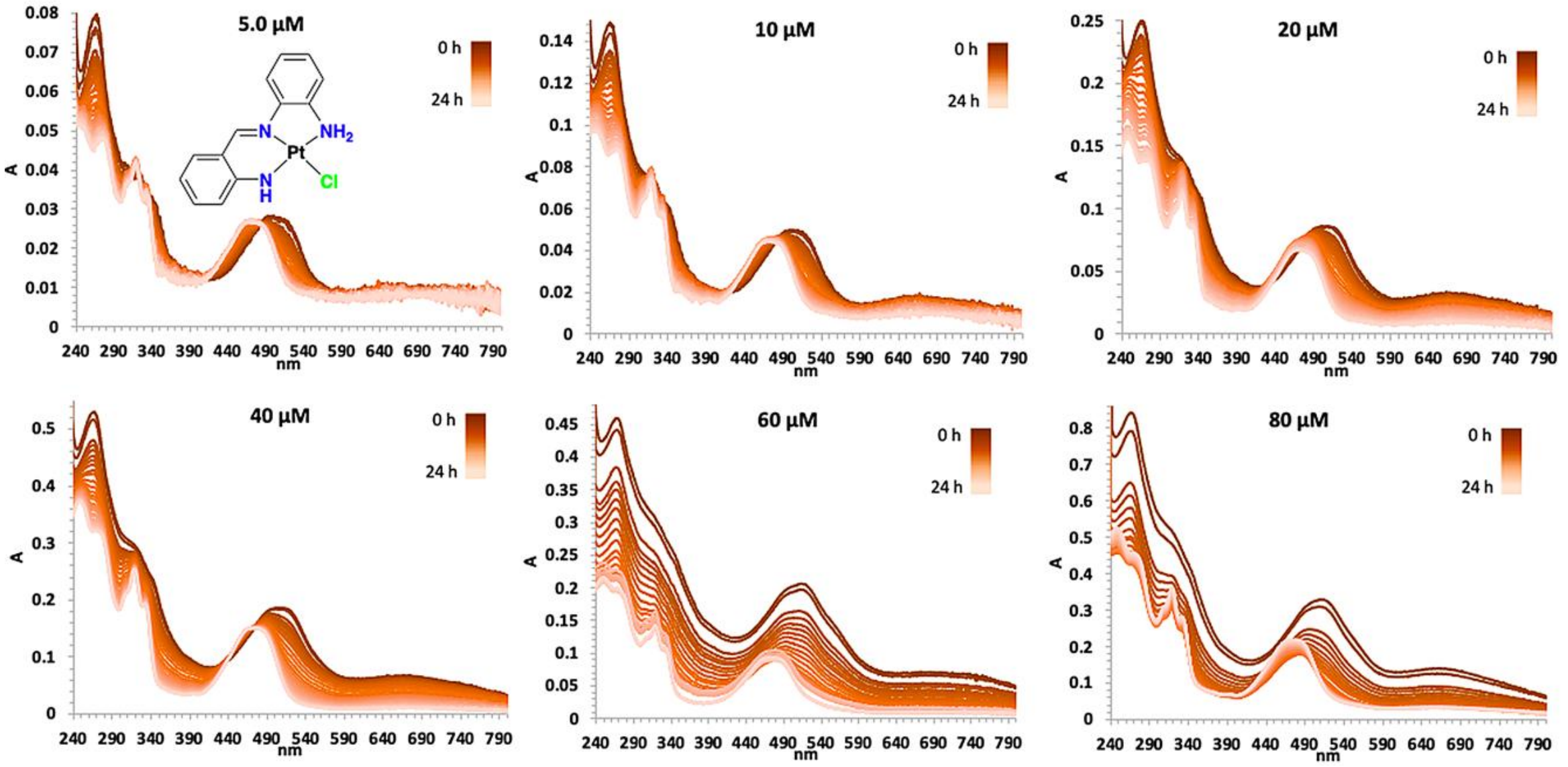

*Figure SI 49. UV-Vis stability of complex **[PtCl(L₁)]** at 37 °C in NaClO₄ 10 mM at 1% (v/v) DMSO for 24 h at different concentrations.*

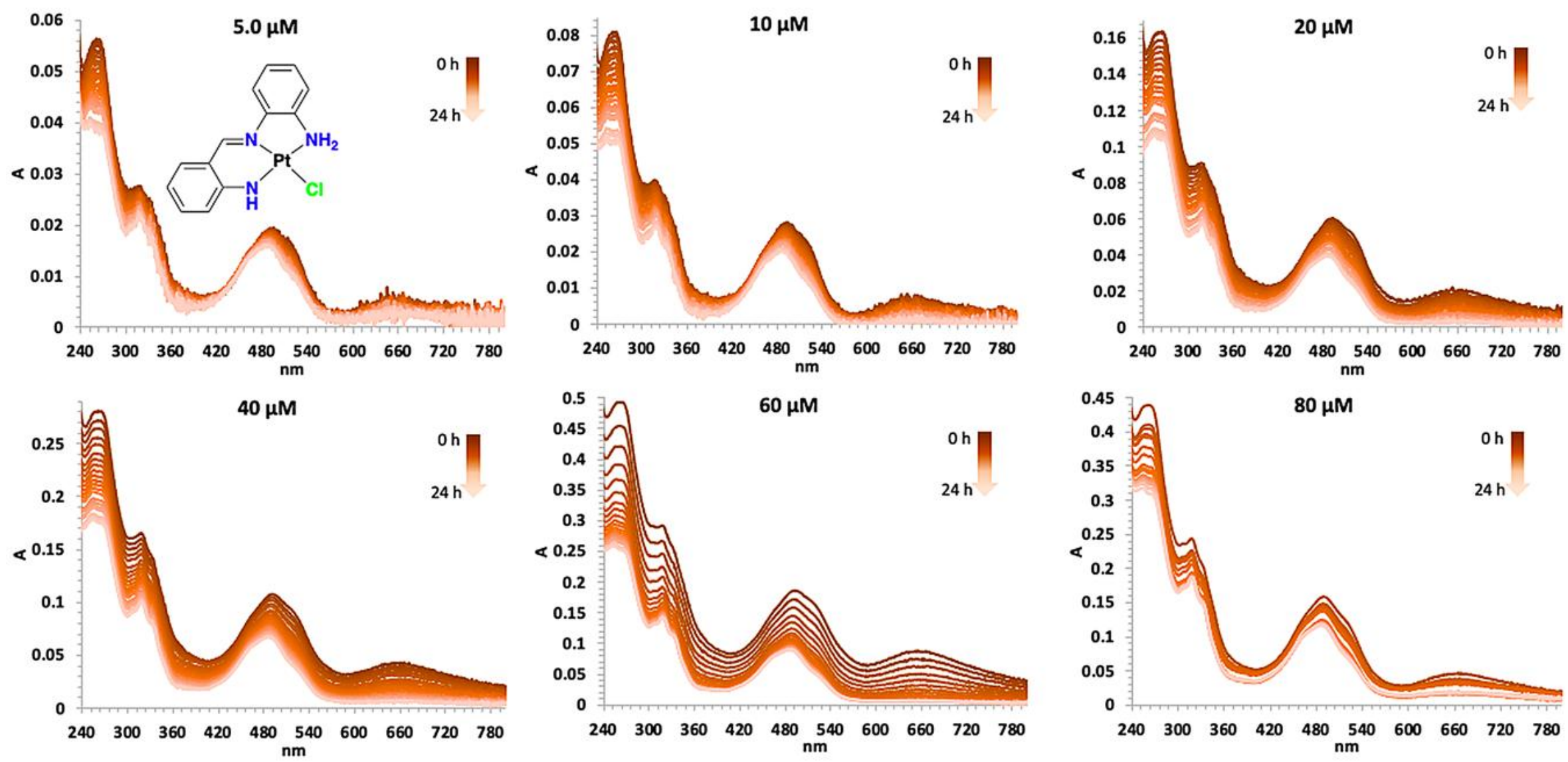

*Figure SI 50. UV-Vis stability of complex **[PtCl(L¹)]** at 37 °C in 0.9% (w/v) NaCl at 1% (v/v) DMSO for 24 h at different concentrations.*

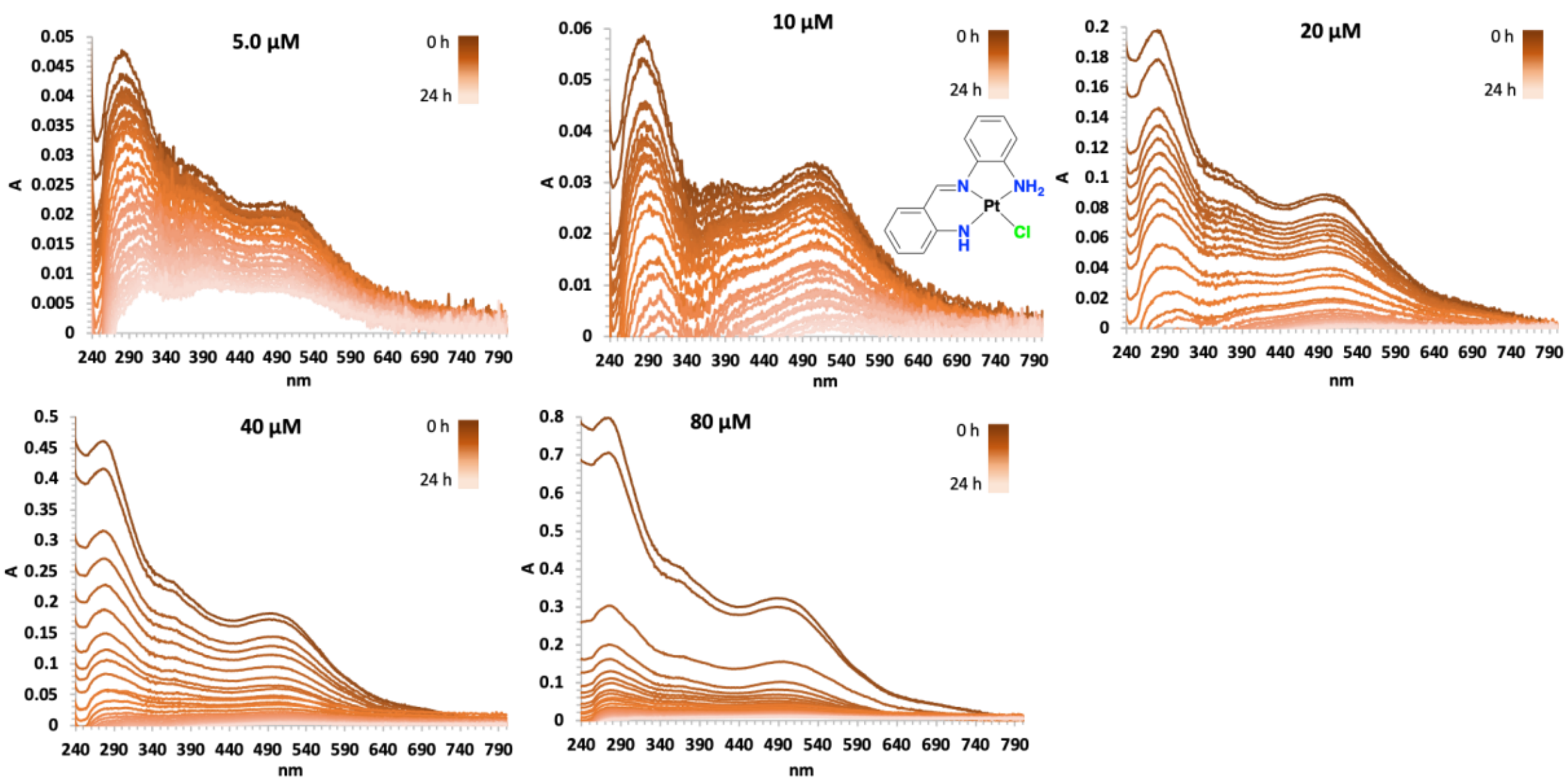

*Figure SI 51. UV-Vis stability of complex **[PtCl(L[1])]** at 37 °C in buffer phosphate 20 mM, pH 7.4, at 1% (v/v) DMSO for 24 h at different concentrations.*

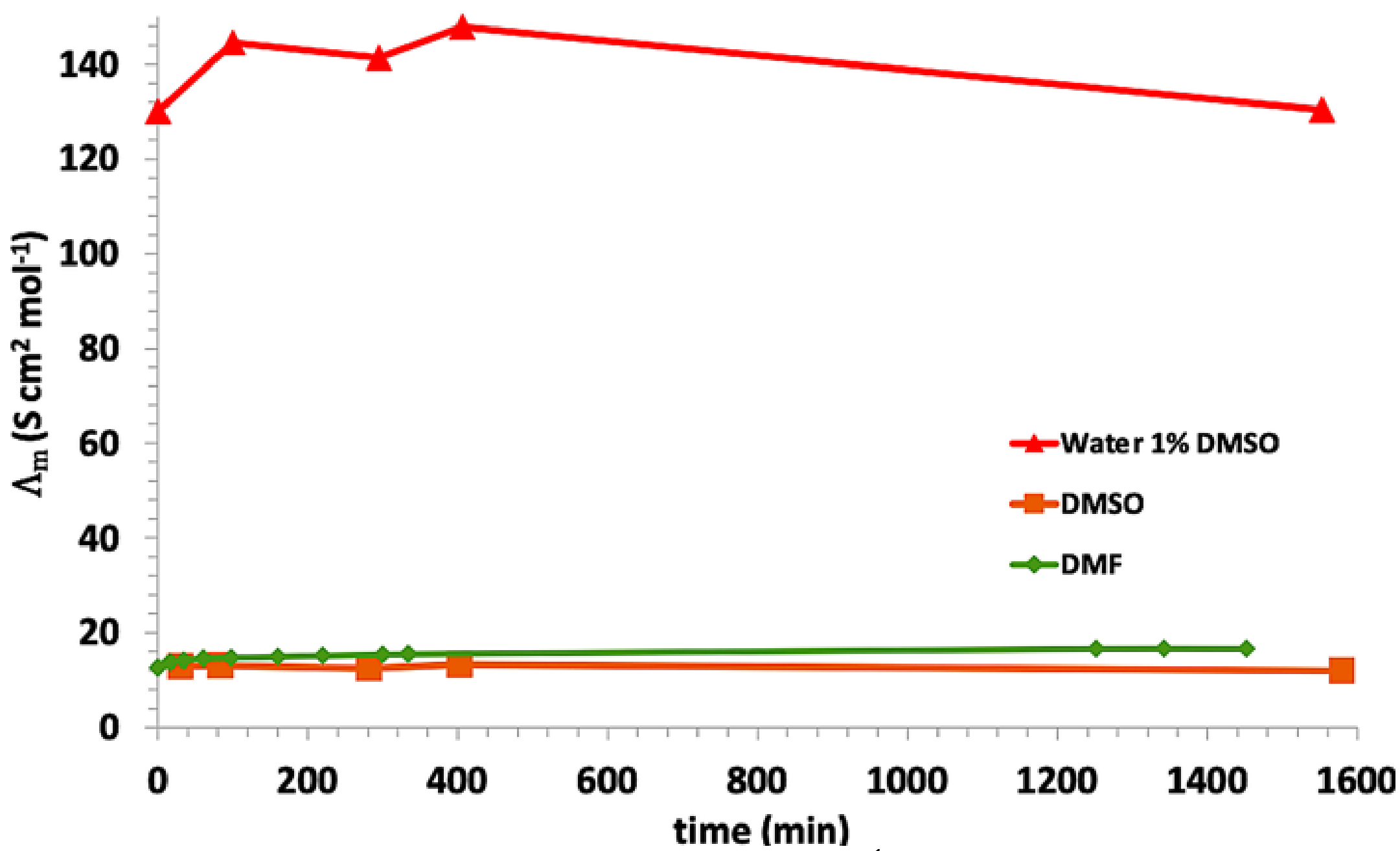

*Figure SI 52. Conductivity stability test of a 1 mM solution of **[PtCl(L[1])]** at 25 °C in water (1% (v/v) DMSO) (red) DMF (green) and DMSO (orange) for 24 hours.*

Supplementary graphs and data supporting biological studies performed with PtCl(L^1H)]Cl and [PtCl(L^1)].

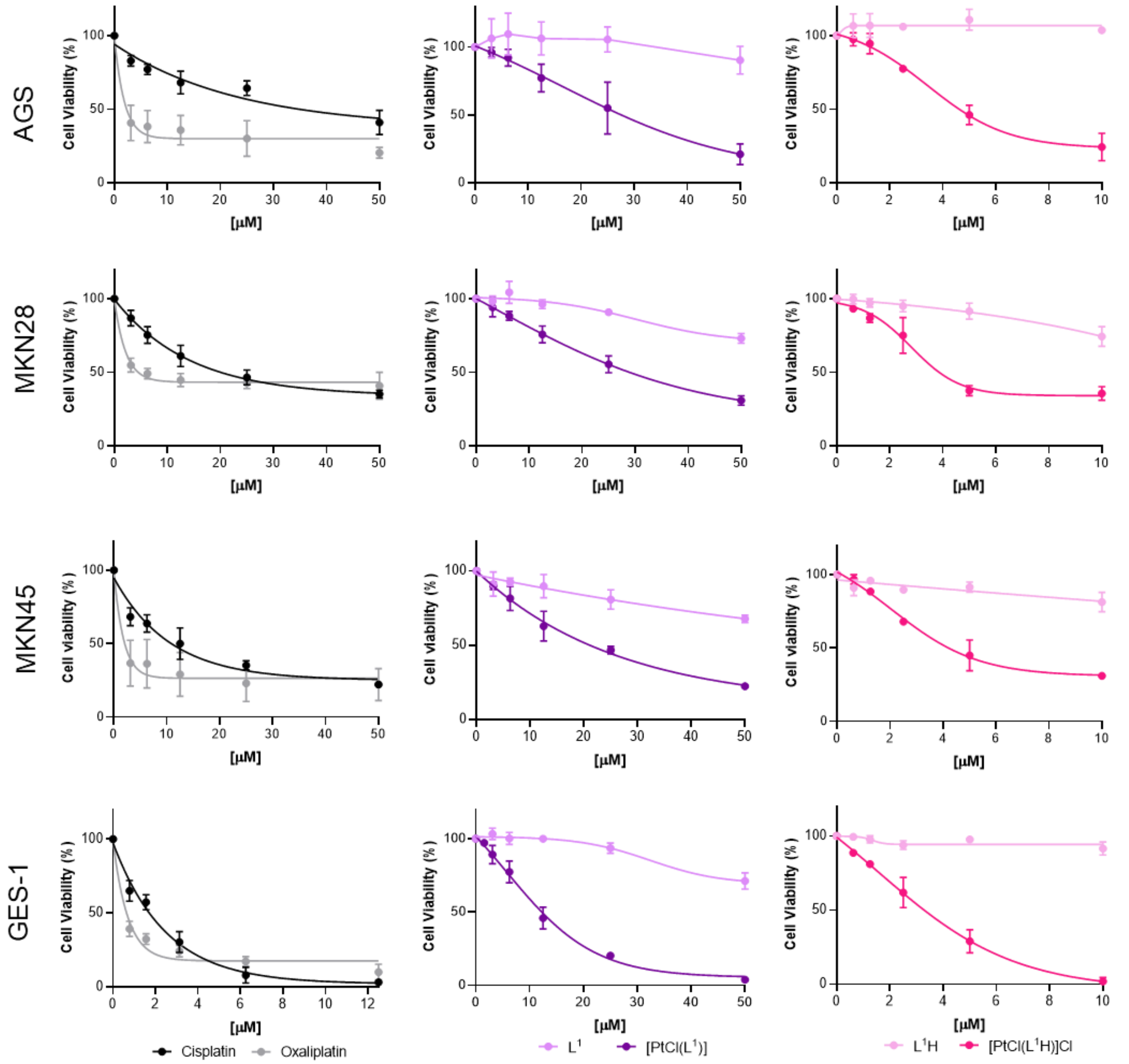

Figure SI 53. Cell viability studies with increasing concentrations (0 – 50 μM) of cisplatin (black), oxaliplatin (grey), ***L^1*** *(light purple),* ***[PtCl(L^1)]*** *(dark purple),* ***L^1H*** *(light pink)) and* ***[PtCl(L^1H)]Cl*** *(dark pink, 0-10 μM), in gastric cancer cell lines: AGS, MKN28, MKN45 and in non-tumoral HPDE cells after 48 h-treatment. The percentage of viable cells was quantified by the CV assay. Data represent the mean values obtained in three experiments performed in quadruplicate.*

a)

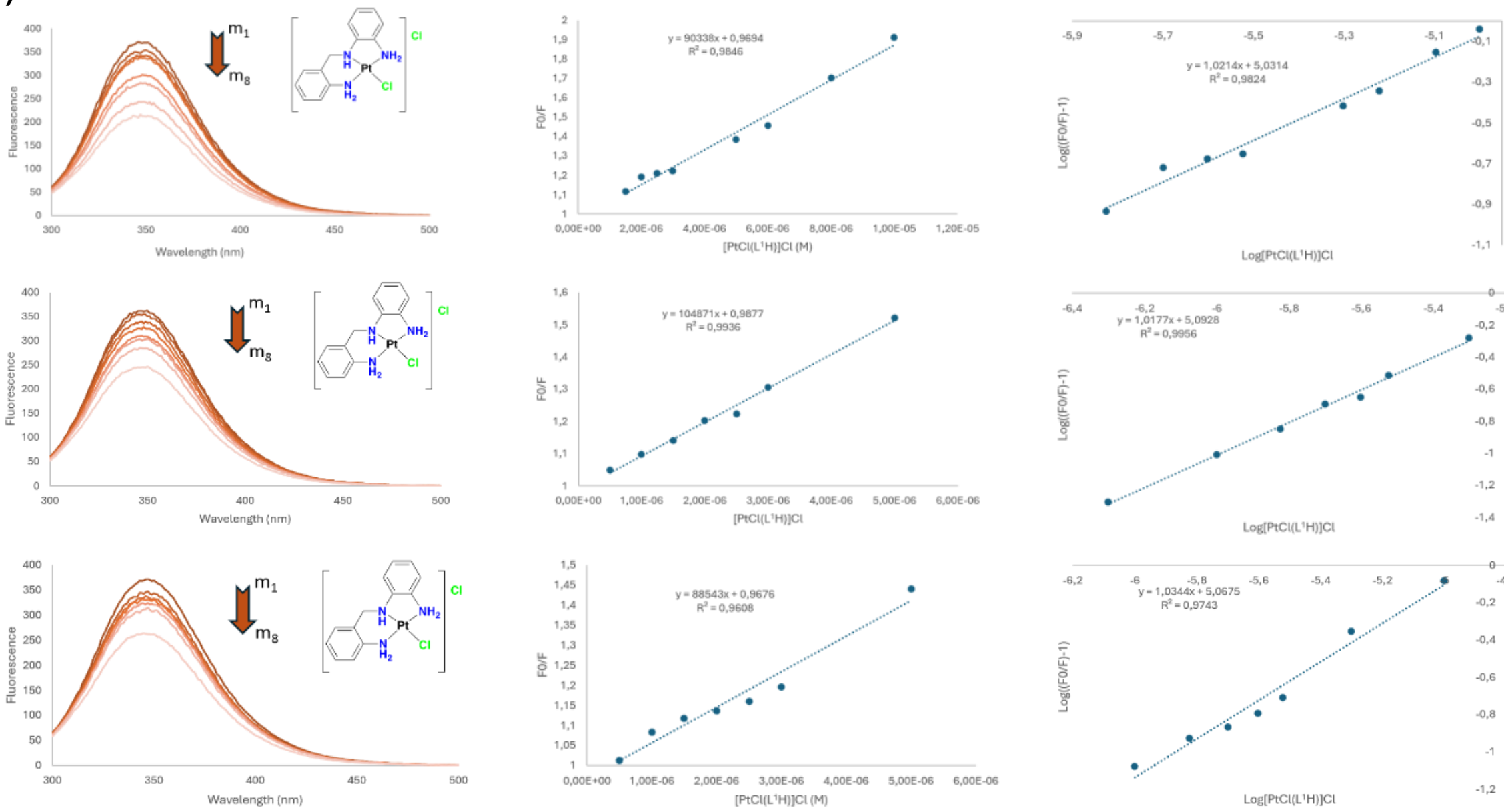

b)

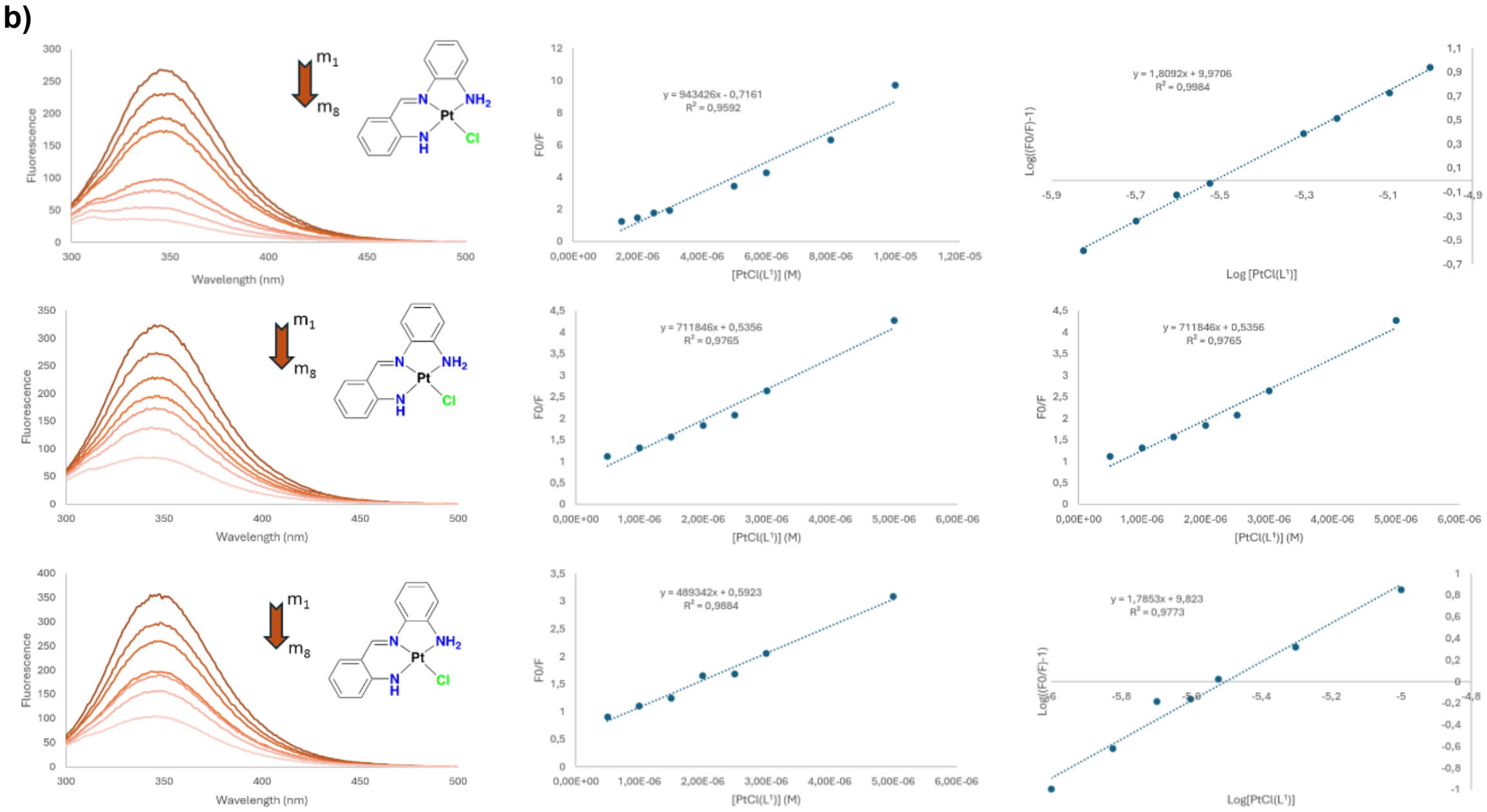

Figure SI 54. Emission spectrum of BSA as a function of concentration of complexes a) **[PtCl(L¹H)]Cl** and b) **[PtCl(L¹)]** ($0.15^{-1}x10^{-5}$ M) in Tris-HCl 5 mM (1% DMSO for **[PtCl(L¹)]**). The arrow indicates the effect of metal complexes on the fluorescence emission of BSA. Stern-Volmer plots employed to calculate the quenching capability (K_{SV}), the binding constant (K_{BIN}) and the number of binding sites. This figure contains the spectra and corresponding plots for the three independent experiments.

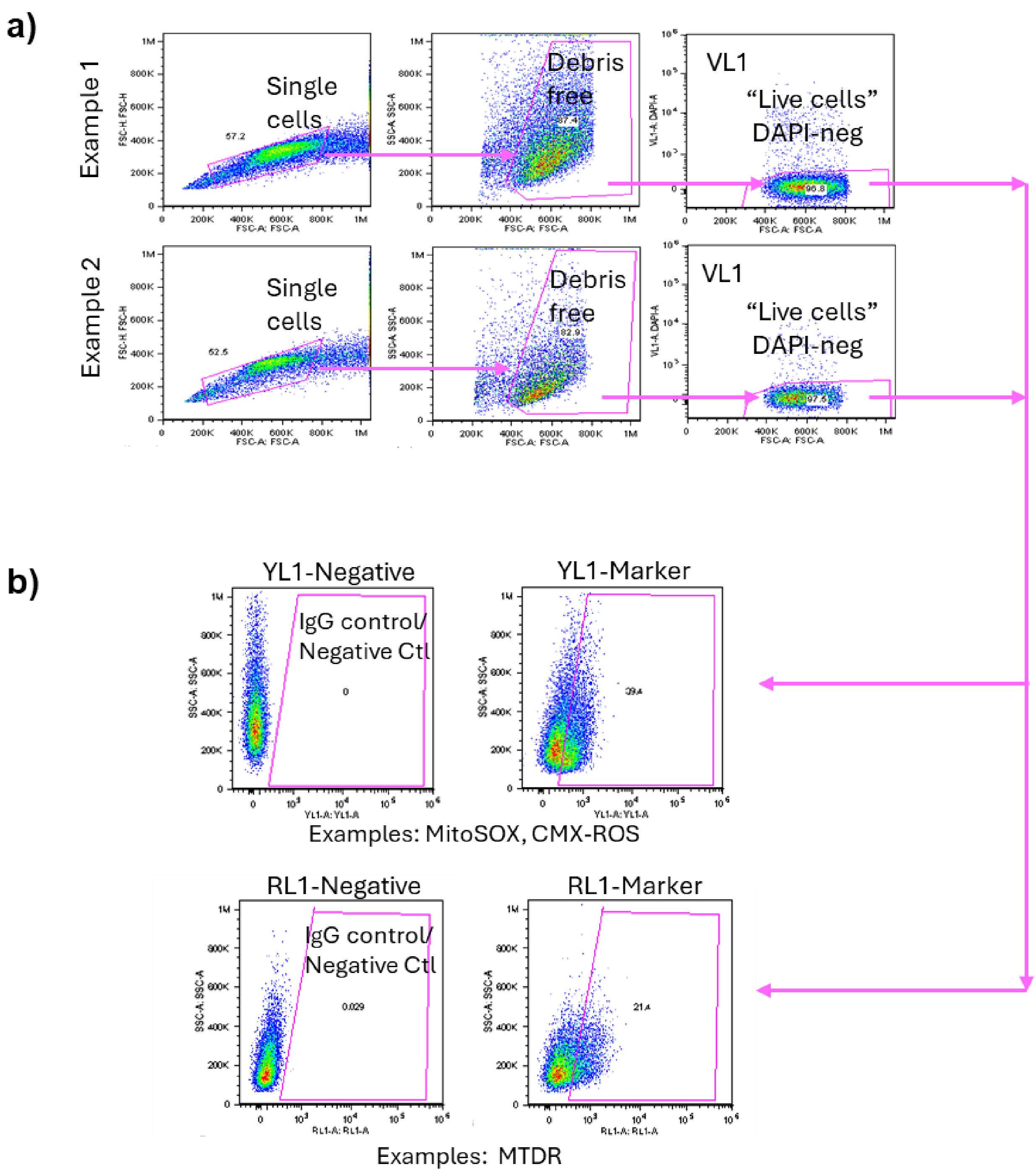

*Figure SI 55. **Flow cytometry gating strategies: a)** Initial gating strategy to achieve live cell population (two examples showing initial gating strategies to acquire a single cell, debris free and DAPI-free ("live cell") population, applied to all analyses). **b)** Gating strategies for different fluorescent channels detected within "live cells". Indicated below are the specific markers or probes for which the indicated gating strategy was applied. Additional details can be found in the Methods section.*

All uncropped blots data

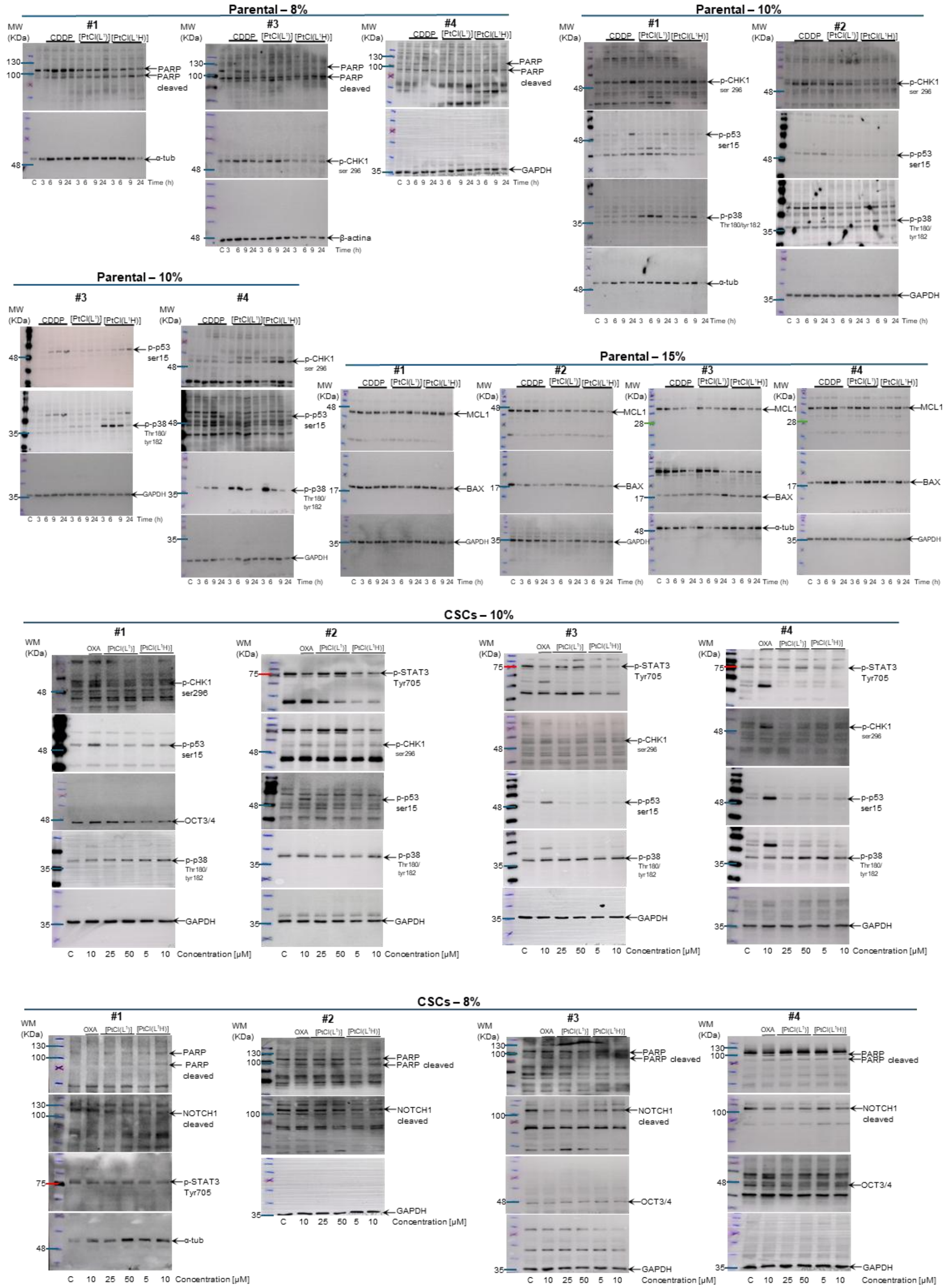

Figure SI 56. Uncropped western blotting images.
